# Mechanism of degrader-targeted protein ubiquitinability

**DOI:** 10.1126/sciadv.ado6492

**Published:** 2024-10-11

**Authors:** Charlotte Crowe, Mark A. Nakasone, Sarah Chandler, Conner Craigon, Gajanan Sathe, Michael H. Tatham, Nikolai Makukhin, Ronald T. Hay, Alessio Ciulli

**Affiliations:** ^1^ Centre for Targeted Protein Degradation, School of Life Sciences, University of Dundee, 1 James Lindsay Place, Dundee DD1 5JJ, UK.; ^2^ Division of Biological Chemistry and Drug Discovery, School of Life Sciences, University of Dundee, James Black Centre, Dow Street, Dundee DD1 5EH, UK.; ^3^ Division of Molecular, Cellular and Developmental Biology, School of Life Sciences, University of Dundee, Dow Street, Dundee DD1 5EH, UK.

## Abstract

Small-molecule degraders of disease-driving proteins offer a clinically proven modality with enhanced therapeutic efficacy and potential to tackle previously undrugged targets. Stable and long-lived degrader-mediated ternary complexes drive fast and profound target degradation; however, the mechanisms by which they affect target ubiquitination remain elusive. Here, we show cryo-EM structures of the VHL Cullin 2 RING E3 ligase with the degrader MZ1 directing target protein Brd4^BD2^ toward UBE2R1-ubiquitin, and Lys^456^ at optimal positioning for nucleophilic attack. In vitro ubiquitination and mass spectrometry illuminate a patch of favorably ubiquitinable lysines on one face of Brd4^BD2^, with cellular degradation and ubiquitinomics confirming the importance of Lys^456^ and nearby Lys^368^/Lys^445^, identifying the “ubiquitination zone.” Our results demonstrate the proficiency of MZ1 in positioning the substrate for catalysis, the favorability of Brd4^BD2^ for ubiquitination by UBE2R1, and the flexibility of CRL2 for capturing suboptimal lysines. We propose a model for ubiquitinability of degrader-recruited targets, providing a mechanistic blueprint for further rational drug design.

## Teaser

PROTAC-targeted protein ubiquitination specificity revealed by structures with cullin RING E3 ligase and E2-ubiquitin.

## INTRODUCTION

Targeted protein degradation has emerged as a powerful modality of chemical biology and therapeutic intervention against proteins that drive disease (*1*). The approach most developed to date involves the design or discovery of small molecules, so-called degraders, that harness the activity of the endogenous ubiquitin-proteasome system to induce ubiquitination and subsequent degradation of target proteins (*2*). This is most effectively achieved by recruiting a *neo*-substrate (i.e., a protein not normally processed as a native substrate) to the key enzymatic machineries that catalyze protein ubiquitination, namely, the ubiquitin E3 ligases, and predominantly the Cullin-RING ligases (CRLs) (*3*–*5*). Degraders are typically categorized as either PROteolysis Targeting Chimeras (PROTACs), i.e., bifunctional molecules composed of a binder to the E3 ligase and a binder to the protein target, joined by a linker unit (*6*); or monovalent for binding to either E3 ligase or target, referred to as molecular glues (*7*). PROTACs, since their development and validation as active in cells and in vivo (*8*–*10*), have emerged as a rapidly growing area of research in both academic research and drug discovery, as illustrated by the >25 drug candidates that are currently in clinical trials for various diseases (*6*, *11*). The vast majority of PROTAC degraders co-opt the activity of one of two CRLs: the von Hippel–Lindau Cullin 2 ligase complex (CRL2^VHL^) (*12*–*14*) and the cereblon Cullin 4 ligase complex (CRL4^CRBN^) (*15*–*17*), which is also the target of molecular glue degraders thalidomide and lenalidomide, among others (*18*, *19*). The repertoire of hijackable E3 ligases has recently expanded as small-molecule ligands are being developed for more Cullin RING E3s and used in PROTACs (*20*), e.g., DCAF1 (*21*) and KLHDC2 (*22*), and more E3s are being identified as co-opted by molecular glue degraders, including DCAF15 (*23*), DCAF16 (*24*), and the CRL4 adaptor DDB1 (*25*), among others. Despite their chemical distinction, PROTACs and molecular glues converge on the same mechanism of action that requires the formation of the ternary complex E3:degrader:target as the key species driving productive target ubiquitination and subsequent proteasomal degradation (*13*, *26*–*28*). However, most structural, biophysical, and mechanistic studies on PROTAC ternary complexes disclosed to date have been restricted to the substrate receptor/adaptor components of the CRL (*13*, *17*, *29*), thus lacking the fully assembled catalytically active CRL complex (*30*). Furthermore, mechanistic and structural investigations of PROTAC-mediated ubiquitination have remained sparse or limited in resolution (*31*–*33*). To fully enable and guide the design and optimization of degrader drugs, there is a growing need to understand how degraders recruit the whole native catalytic enzymatic machinery to illuminate their mechanism of action.

**Fig. 1.**
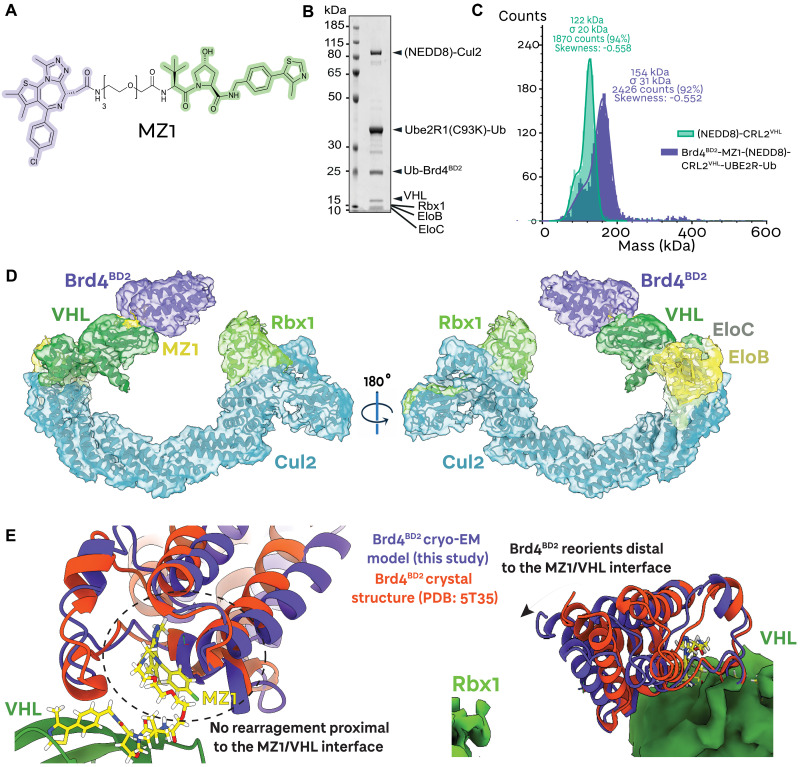
MZ1 orients the Brd4 bromodomain 2 *neo*-substrate toward Rbx1. (**A**) Chemical structure of the bivalent PROTAC MZ1 (in purple: the Brd4^BD2^ ligand; in green: the VHL ligand). (**B**) SDS-PAGE of the assembled Brd4^BD2^-MZ1-(NEDD8)-CRL2^VHL^-UBE2R1(C93K)-Ub complex applied to cryo-EM grids. (**C**) Mass photometry histogram of the fully assembled Brd4^BD2^-MZ1-(NEDD8)-CRL2^VHL^-UBE2R1(C93K)-Ub complex (purple) applied to cryo-EM grids, versus (NEDD8)-CRL2^VHL^ (green). The peaks have been fitted with Gaussian curves. (**D**) The ~4.0-Å-resolution cryo-EM reconstruction of Brd4^BD2^-MZ1-(NEDD8)-CRL2^VHL^. The E3 ligase NEDD8-CRL2^VHL^ (green) recruits MZ1 (yellow), which, in turn, binds the *neo*-substrate Brd4^BD2^ (purple). A ribbon representation of the complex is modeled into the displayed cryo-EM volume (same color scheme). (**E**) The cryo-EM model (this study) of the VHL-MZ1-Brd4^BD2^ interface overlaid with the same interface from the crystal structure by Gadd *et al.* (*13*) (PDB: 5T35). Brd4^BD2^ displays a conserved interface proximal to the MZ1-VHL binding site, and a re-orientation distal to the MZ1-VHL binding site, toward the E3 ligase RING box domain.

Structural and mechanistic features of degrader-mediated ternary complexes directly affect the pharmacological activity of degraders and thus represent an important optimization species for rational structure-based drug design (*26*). Multiple studies have revealed that thermodynamically cooperative, stable, and kinetically long-lived degrader-mediated ternary complexes, in most cases, underpin efficient and selective protein degradation profiles of degraders (*13*, *29*, *34*–*39*). Yet, how ternary complex formation influence productive and selective *neo*-substrate ubiquitination within the PROTAC-co-opted catalytic mechanism of the multiprotein CRL complex has to date remained largely elusive. To this end, we decided to investigate the structure and mechanisms of the PROTAC MZ1 (Fig. 1A), our potent and fast VHL-recruiting Bromodomain and Extra-Terminal Domain (BET) degrader, which exhibits preferential degradation of Brd4 over other BET proteins (*8*, *29*, *40*). MZ1 is a well-characterized and widely used PROTAC degrader whose binding and ternary complex formation has been probed by various biophysical and structural methods by us and others since our first PROTAC cocrystal structure published in 2017 (*13*, *29*, *39*, *41*–*43*). These studies have revealed that MZ1 recruits all BET bromodomains to VHL with positive cooperativity, yet despite binding all BET-BDs with comparable binding affinity at the binary level, it preferentially recruits the BD2s over the BD1s in ternary complexes. Of note, MZ1 forms the most cooperative, stable, and long-lived ternary complex with Brd4^BD2^, even over its close homolog Brd3^BD2^, explaining its preferential Brd4 degradation selectivity (*13*, *29*). Brd4^BD2^ is the BET bromodomain isoform that has been cocrystallized by far the most to date, not only with MZ1 but also with other VHL-based PROTACs including our structure-based designed macroPROTAC-1 (*41*) and AT7 (*44*), and other analogs (*45*). In all these cocrystal structures, the orientation of the bromodomain relative to VHL within the ternary complex is very similar, highlighting a highly conserved substrate binding mode.

## RESULTS

To gain a deeper understanding of degraders’ mechanism of action, we determined structures of multi-subunit catalytic complexes, composed of the full CRL2^VHL^, an E2 conjugating enzyme loaded with ubiquitin, and Brd4^BD2^ as the “*neo*-substrate,” all assembled together by MZ1 as a representative PROTAC degrader. CRL2^VHL^ is a 150-kDa E3 ligase composed of five subunits: the substrate receptor von Hippel–Lindau (short isoform VHL19, 18 kDa or long isoform VHL30, 24 kDa), the adaptor proteins Elongin C (EloC, 12 kDa) and Elongin B (EloB, 15 kDa), the Cullin 2 scaffold (Cul2, 87 kDa), and the RING box protein 1 (Rbx1, 12 kDa). VHL-EloC-EloB (VCB) is recruited to the N terminus of Cul2 at the interface between VHL and EloC (*12*, *46*). At the C-terminal domain of cullin 2, the E1 NEDD8-activating enzyme APPBP1-UBA3 and the E2 NEDD8-conjugating enzyme UBE2M work with the NEDD8 E3 ligase Rbx1 to neddylate Cul2, i.e., to covalently modify the K689 residue of the Cul2 WHB domain with NEDD8 (*47*, *48*). This modification has been shown to induce conformational rearrangements of cullin-RING complexes as well as facilitate engagement of E2 ubiquitin-conjugating enzymes (*49*–*51*). We therefore set out to determine the structures of fully active NEDD8-CRL2^VHL^ with Brd4^BD2^ using single-particle cryo–electron microscopy (cryo-EM). The initial focus was on the fully assembled Brd4^BD2^-MZ1-(NEDD8)-CRL2^VHL^-E2-Ub complex. We expressed and purified each CRL2^VHL^-complex component, namely, VCB and Cul2-Rbx1 separately, then in vitro neddylated the assembled VCB-Cul2-Rbx1 (CRL2^VHL^) complex, purified it by size-exclusion chromatography, and showed that our NEDD8-CRL2^VHL^ was catalytically competent (fig. S1). As for the E2 conjugating enzyme, we leveraged our previously reported strategy of engineering an active site Cys-to-Lys mutant suitable for loading donor-ubiquitin via a stable isopeptide bond (*52*), and designed, assembled, and purified in vitro a mono donor-ubiquitin adduct for the poly-ubiquitinating E2 enzyme UBE2R1 [also known as Cdc34 (*53*)], referred herein as UBE2R1(C93K)-Ub (fig. S2). We next reconstituted a full Brd4^BD2^-MZ1-(NEDD8)-CRL2^VHL^-UBE2R1(C93K)-Ub complex, and evidenced the mono-dispersed nature of its full assembly using mass photometry as deemed suitable for cryo-EM grid sample preparation (Fig. 1, B and C). Given the complex preference to “lie flat” in the cryogenic sample, the reconstructed cryo-EM volume presented anisotropic artifacts; nonetheless, the map was of sufficient quality to build atomic models.

Our ~4.0-Å-resolution cryo-EM structure presents the orientation of the *neo*-substrate Brd4^BD2^ in complex with a PROTAC and full-length active E3 ligase (Fig. 1D; see fig. S3 for cryo-EM data analysis). We could confidently build atomic models corresponding to Brd4^BD2^-MZ1-CRL2^VHL^ within the volume. Despite the E2~Ub conjugate being present in this sample, data processing at the 2D classification level showed a much lower population of substrate-PROTAC-E3-E2-Ub compared to just substrate-PROTAC-E3 (fig. S3), likely reflecting the rapid dynamics of assembly and disassembly of the E3-E2 complex (*54*, *55*). Distinct crystal structures and cryo-EM structures of non-neddylated Cul2-Rbx1 had previously revealed that the WHB domain of Cul2 and Rbx1 are closely packed (*30*, *56*, *57*) (fig. S4). Cul2 neddylation has been shown to abolish these contacts, allowing Rbx1 to be “freed” and recruit E2~Ub (*30*). This was indeed observed in our structure, where volume corresponding to Rbx1 is observed, albeit at lower resolution relative to the rest of the structure due to its mobility, pointing toward the substrate (fig. S4). We did not observe density for NEDD8 or the WHB domain of Cul2, also consistent with the highly dynamic neddylation state of the CRL2 (Fig. 1D).

Our previous models of full-length CRL2^VHL^ based on structural alignments and superpositions had suggested that the substrate-binding interface of VHL is located ~45 Å from the Rbx1 domain (fig. S5A) (*12*, *46*). In our cryo-EM structure, the *neo*-substrate Brd4^BD2^ is recruited to the substrate receptor VHL in the same overall orientation as observed in our previous cocrystal structure (*13*), with no notable rearrangement at the VHL-Brd4 protein-protein interface as “glued” by MZ1, consistent with the tight neo-interactions mediated by MZ1 within the ternary complex (Fig. 1E, left). Nonetheless, in our cryo-EM consensus volume, we observed a slight “tilt” of the bromodomain toward Rbx1 (Fig. 1E, right), bringing the gap between substrate receptor and the Rbx domain down to ~10 Å (fig. S5B). The “open ring”–like structure suggested that an E2-donorUb conjugate could potentially approach substrate lysines or an acceptor ubiquitin within this ~10-Å gap. Of note, while this shortened gap is consistent with what is observed with other PROTAC-bound cocrystal structures of VHL with Brd4^BD2^ (fig. S5, C and D), other ternary crystal structures with Brd4^BD1^ and FKBP51^FK1^ show the *neo*-substrate pointing in a different direction, away from Rbx1 (fig. S5, E to H). Together, our full-length CRL2^VHL^-MZ1-Brd4^BD2^ tertiary structure validates the crystallographic pose of the *neo*-substrate relative to VHL and reveals subtle flexibility within the system allowing bridging of the gap between the substrate-binding and the catalytic sites of the E3 ligase toward ubiquitination.

**Fig. 2.**
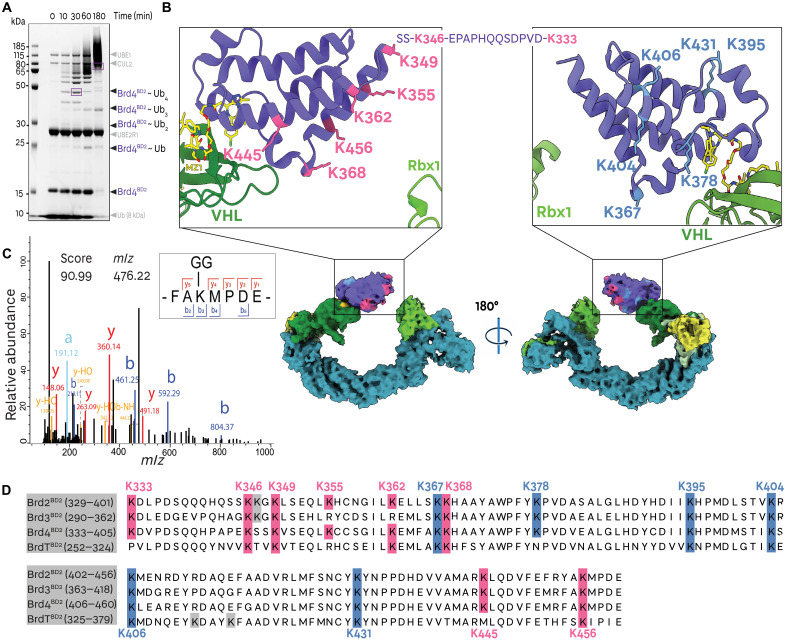
Brd4 bromodomains are ubiquitinated at lysines accessible to the E3 ligase RING box domain. (**A**) Coomassie-stained SDS-PAGE of in vitro Brd4^BD2^ ubiquitination in the presence of MZ1-CRL2^VHL^-UBE2R1-Ub (in purple: gel bands subjected to mass spectrometry analysis). (**B**) Cryo-EM volume of Brd4^BD2^ in complex with MZ1-(NEDD8)-CRL2^VHL^ with ubiquitinated lysines on Brd4^BD2^ highlighted in pink and unmodified lysines highlighted in light blue as mapped by mass spectrometry following in vitro ubiquitination. (**C**) Identified mass spectrum for the Brd4^BD2^ K-GG–modified peptide at K456. “Score” refers to the Andromeda score. (**D**) Sequence alignments of the bromodomain 2 of Brd2, Brd3, Brd4, and BrdT. Ubiquitinated lysines on Brd4^BD2^ (as shown by mass spectrometry) are highlighted in pink and unmodified lysines are highlighted in light blue, with sequence-aligned lysines of other BD2s represented in the same color, suggesting which lysines may be modified in other BET BD2s.

We next aimed to identify which lysine residues on the Brd4 bromodomain are most accessible to and targeted for ubiquitination. In vitro ubiquitination products with both UBE2R1 and UBE2D2 [also known as UbcH5b, which is thought to preferentially prime a substrate with a first ubiquitin (*58*)] were resolved by SDS–polyacrylamide gel electrophoresis (SDS-PAGE), bands of Brd4-ubiquitinated products were excised and digested with trypsin, and K-GG-modified peptides were identified by mass spectrometry (MS; Fig. 2A and fig. S6). We consistently identified eight ubiquitination sites on Brd4^BD2^ (K333, K346, K349, K355, K362, K368, K445, and K456), each from at least one peptide identified in the MS with Andromeda score greater than 100 (see Materials and Methods). This overall cluster of ubiquitination sites is seen at 3 hours both in the excised UBE2R1-catalyzed octa-Ub-Brd4^BD2^ product (79 kDa, Fig. 2A and fig. S7) and in the products analyzed from the UBE2D2-catalyzed reaction (39, 47, and 79 kDa, for tri-, tetra-, and octa-Ub, respectively, fig. S7). All the modified lysines cluster on the face of Brd4^BD2^ closest to Rbx1, as well as on the unstructured N-terminal tail of the bromodomain (Fig. 2B, lysine residues highlighted in pink). In contrast, lysines on the opposing face were not identified as being ubiquitinated in any of our samples (Fig. 2B, lysine residues highlighted in blue). In an attempt to identify some preferential Lys and hint to ubiquitination specificity, we also excised bands at shorter time points and identified Lys-modified peptides for a mono-Ub-Brd4 ^BD2^ product of the UBE2D2 reaction at 1 hour (figs. S6 and S7) and for a tetra-Ub-Brd4^BD2^ product of the UBE2R1 reaction at 30 min (Fig. 2A and figs. S6 and S7). While seven of eight lysines were found to be modified in the product of the UBE2D2-catalyzed reaction, suggesting a mixture of different monoubiquitinated Brd4^BD2^ products, we found only one peptide corresponding to K456 ubiquitination in the UBE2R1-catalyzed tetra-Ub-Brd4^BD2^ product (Fig. 2C and fig. S7). It is likely that this difference reflects the relatively slower intrinsic catalytic activity of the elongating E2 UBE2R1 at placing the first ubiquitin on a substrate, as compared to UBE2D2, such that it is much more discriminatory for Lys residues at the short time points, thus suggesting that K456 may be one of the most preferentially ubiquitinated residues in the MZ1-bound Brd4^BD2^ mechanism. Although the only K-GG–modified peptide identified in this sample was K456 (Fig. 2C and fig. S7), it cannot be excluded that other sites of ubiquitination were present but were not detected by this analysis. Nonetheless, K456 also had the highest Andromeda score and intensity among all modified lysines in the UBE2D2-catalyzed mono-Ub-Brd4 ^BD2^ product and featured the highest intensity in all the other analyzed samples. Analysis of the ubiquitin peptides in the UBE2R1-produced tetra-Ub-Brd4^BD2^ sample exhibited a mixture of ubiquitin modification sites with a predominant intensity of K48 linkages, consistent with the proposition that UBE2R family E2s preferentially extend K48-linked chains (*58*), and suggesting that at least in part the sample contains multiply ubiquitinated K456 modified Ub-Brd4 ^BD2^ species (fig. S7). With both UBE2R1 and UBE2D2, K48 and K11 ubiquitin linkage intensities were dominant (in addition to K63 for UBE2D2), consistent with the building of degradative poly-ubiquitin chains on Brd4^BD2^ by these E2s.

As MZ1 exhibits ubiquitination and degradation selectivity for Brd4 and Brd2 over Brd3 (*13*, *29*, *40*), we inspected the conservation of the identified ubiquitinated lysine residues from sequence alignments of the BD2 in the different BET proteins (Fig. 2D and fig. S8). The K456 position is found to be strictly conserved among the BET BD2s, and so are K346, K349, and K368, while other two residues (K333 and K445) are strictly conserved among the ubiquitously expressed Brd2/3/4 but not on BrdT (Fig. 2D). There are, however, two residue positions (K355 and K362 in Brd4^BD2^) that bear a ubiquitinable Lys in Brd4^BD2^ and Brd2^BD2^ but a nonubiquitinable Arg residue in Brd3^BD2^. While the main contributor and driver of the mechanistic selectivity for Brd4^BD2^ and Brd2^BD2^ over Brd3^BD2^ resides largely on the preferential neo-substrate recognition within the ternary complex (*13*, *29*), this analysis suggests that it is possible at least partially that the presence of these two ubiquitinable Lys residues in Brd2/4 but not Brd3 could also contribute to Brd4^BD2^ selectivity. Together, our ubiquitination data identify a cluster of ubiquitinable lysines that reside on the face of the target protein closest to Rbx1 (herein called “light face”), clearly distinct from those present in the opposite face, which are nonubiquitinable (named “dark face”). We also identify a single Lys residue, which appears to be preferentially ubiquitinable, while overall lysine conservation and positioning cannot clearly explain the mechanistic selectivity of MZ1 for the different BET BD2s.

**Fig. 3.**
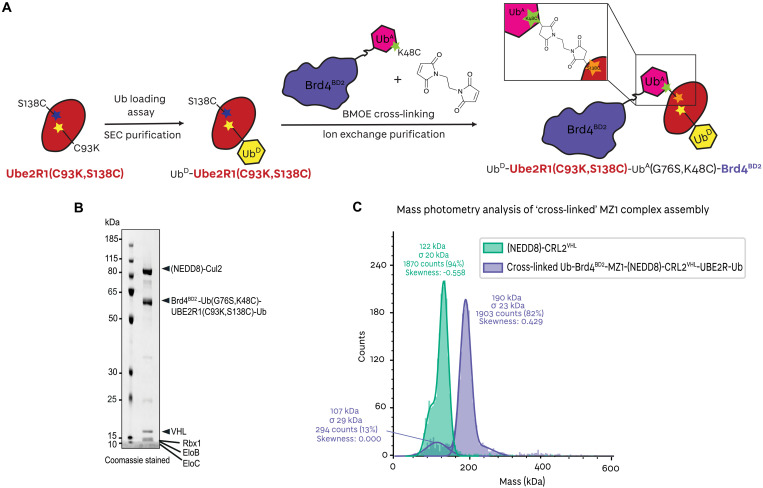
Assembly of a Brd4^BD2^ ubiquitination structural mimetic. (**A**) Schematic of the biochemical strategy used to trap MZ1-Brd4^BD2^-Ub(G76S, K48C)-UBE2R1(C93K, S138C, C191S, C223S)-Ub in complex with (NEDD8)-CRL2^VHL^. (**B**) SDS-PAGE of the assembled (NEDD8)-CRL2^VHL^-MZ1-Brd4^BD2^-Ub(G76S, K48C)-UBE2R1(C93K, S138C, C191S, C223S)-Ub complex applied to cryo-EM grids. (**C**) Mass photometry histogram of the fully assembled (NEDD8)-CRL2^VHL^-MZ1-Brd4^BD2^-Ub(G76S, K48C)-UBE2R1(C93K, S138C, C191S, C223S)-Ub complex (purple) applied to cryo-EM grids, versus (NEDD8)-CRL2^VHL^ (green). The peaks have been fitted with Gaussian curves.

To better understand how cullin RING ligases mediate ubiquitination of *neo*-substrates, we developed a method to increase the occupancy of the E2 on the E3 by capturing transition-state analog species corresponding to the active ubiquitin chain extension on Ub-BRD4^BD2^ by UBE2R1 (Fig. 3A and fig. S9). In this ubiquitination structural mimetic, wild-type Brd4^BD2^ was first N-terminally fused to the C terminus of ubiquitin (G76S and K48C), mimicking an acceptor ubiquitin (Brd4^BD2^-Ub^A^). A UBE2R1(C93K, S138C, C191S, C223S) mutant was generated, as S138 has a role in positioning the nucleophilic substrate near the active C93 site; we therefore mutated Ser^138^ to Cys to allow for maleimide reactivity and mutated C191S and C223S to avoid bismaleimidoethane (BMOE) cross-reactivity. The UBE2R1(C93K, S138C, C191S, C223S) mutant was loaded irreversibly with wild-type ubiquitin via a stable isopeptide bond at the C93K residue (UBE2R1-Ub^D^). The resulting Ub(G76S, K48C)-Brd4^BD2^ was biochemically cross-linked with a small BMOE cross-linker to the S138C residue near the E2 acceptor site of UBE2R1(C93K, S138C, C191S, C223S)-Ub and purified (Brd4^BD2^-Ub^A^-BMOE-Ube2R1-Ub^D^) (Fig. 3A and fig. S9). Upon incubation with MZ1 and (NEDD8)-CRL^VHL^, full complex formation was observed by gel electrophoresis and mass photometry analyses (Fig. 3, B and C). Within the (NEDD8)-CRL2^VHL^-MZ1-Brd4^BD2^-Ub^A^-BMOE-UBE2R1-Ub^D^ complex, MZ1 and the cross-linked substrate-E2 conjugate serve as a stable bridge between the N- and C-terminal regions of CRL2^VHL^, with the goal of encircling the full complex into a closed “ring-like” structure.

To solve the structure of a closed fully assembled complex, we subjected the sample to structural studies by cryo-EM (see fig. S10 for data analysis). Whereas the non–cross-linked “open” cryo-EM structure suffered from anisotropy (Fig. 1 and figs. S3 and S17), these anisotropic effects were reduced with this sample due to imaging in thicker ice of the cryogenic sample at 300 kV, allowing the particles to adopt a wider range of orientation distribution and achieve a higher overall resolution (figs. S10 and S18).

**Fig. 4.**
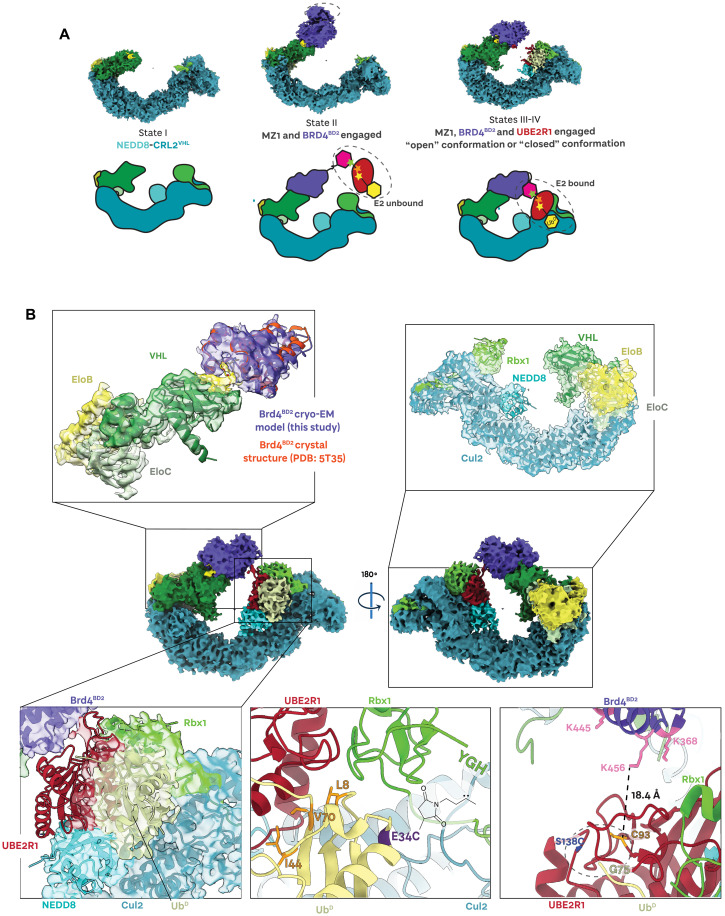
Structural snapshots of Brd4^BD2^ ubiquitination complex assembly. (**A**) Fixed discrete states and continuous states are captured by cryo-EM corresponding to the dynamics of the cross-linked (NEDD8)-CRL2^VHL^-MZ1-Brd4^BD2^-Ub(G76S, K48C)-UBE2R1(C93K, S138C, C191S, C223S)-Ub polyubiquitination species. (**B**) The ~3.7-Å-resolution cryo-EM reconstruction of closed cross-linked (NEDD8)-CRL2^VHL^-MZ1-Brd4^BD2^-Ub(G76S, K48C)-UBE2R1(C93K, S138C, C191S, C223S)-Ub. The E3 ligase (NEDD8)-CRL2^VHL^ (shades of green) recruits MZ1 (yellow), which, in turn, binds the *neo*-substrate Brd4^BD2^ (purple). UBE2R1 (red) is engaged by Rbx1, with donor ubiquitin (pale yellow) covalently bound to UBE2R1(C93K).

With this sample, we solved a number of discrete cryo-EM structures corresponding to fixed states, from a single multiclass ab initio classification. Although mass photometry suggested complete complex formation (Fig. 3C), “state I” corresponding to the (NEDD8)-CRL2^VHL^ was still present, possibly due to the complex partially dissociating in the cryogenic sample (Fig. 4A, left). “State II” corresponding to an open form of (NEDD8)-CRL2^VHL^-MZ1-Brd4^BD2^-Ub^A^-BMOE-UBE2R1-Ub^D^ with MZ1-Brd4^BD2^ visibly engaged with VHL and UBE2R1(C93K, S138C, C191S, C223S)-Ub tethered to Brd4^BD2^-Ub(G76S, K48C) but not engaging with Rbx1 at the C terminus of the CRL2, and likely sampling many conformations around the complex (Fig. 4A, middle). Notably, state II highlights and is consistent with the stability and longevity of the VHL-MZ1-Brd4^BD2^ ternary complex and the rapid association/dissociation kinetics of UBE2R1-CRL2 interaction (*54*). We also obtained a population of species corresponding to a continuum between states III and IV, where the MZ1-Brd4^BD2^-Ub^A^-BMOE-UBE2R1-Ub^D^ central component appears to have successfully bridged the two sides of the (NEDD8)-CRL2^VHL^ by engaging the substrate receptor VHL at one end and the Rbx1-Cul2 C-terminal region at the other end. Owing to the highly dynamic states, the local resolution for the flexible components was reduced (fig. S10F).

In recent years, a number of deep learning neural network models and algorithms have been developed to explore hetereogeneity in cryo-EM samples, including 3DFlex (*59*), cryoDRGN (*60*), 3D variability analysis (3DVA) (*61*), and e2gmm (*62*), among others. To model the conformational landscape of the (NEDD8)-CRL2^VHL^-MZ1-Brd4^BD2^-Ub(G76S, K48C)-UBE2R1(C93K, S138C, C191S, C223S)-Ub complex, we used 3D variability analysis (*61*) to resolve continuous flexibility (movies S1 and S2). For the population of species corresponding to states III and IV, we obtained a consensus volume corresponding to an average state. In this overall ~3.7-Å reconstruction, (NEDD8)-CRL2^VHL^ is locally resolved to ~3.5 Å, whereas Brd4^BD2^-Ub(G76S, K48C) and UBE2R1(C93K, S138C, C191S, C223S)-Ub components display resolutions in a range of 4 to 8 Å (fig. S10F). From this consensus volume, we were able to build an atomic model supported by in-solution NMR and structural cross-linking techniques and knowledge from previously published CRL-E2 structures and our previous open structure.

In this “closed” structure, the VHL-MZ1-Brd4^BD2^ portion conserves its overall geometry (Fig. 4B, top left, and fig. S11) with no major rearrangements seen. The same tilt relative to the cocrystal structure (*13*) is also seen in this closed state, albeit to a lesser extent than observed in the open structure (fig. S11, C and D). As the resolution of this region was higher compared to the open structure, we can now observe density volume for the PROTAC MZ1 itself (yellow in fig. S11, A and B). However, the resolution diminishes as the bromodomain moves away from VHL-MZ1, suggesting the high flexibility of the terminal regions, and no volume is observed corresponding to the acceptor ubiquitin bound to Brd4. The core of the CRL2^VHL^ ligase complex is extremely well resolved as the highest-resolution region in the structure (Fig. 4B, top right). Overlays with our open structure and with our pentameric crystal structure across the whole of Cul2 shows that the overall architecture is strictly conserved, with some minor bending over the first 160 residues of the Cul2 NTD (fig. S12). Volume corresponding to NEDD8 is visible and could be modeled (cyan in Fig. 4B, top right), whereas the WHB domain is not. Pleasingly, we observed enough volume corresponding to the UBE2R1-Ub^D^ conjugate that allowed us to model it bound to Rbx1 (red, yellow, and green, respectively, in Fig. 4B, bottom left). Although the volume corresponding to the E2 did not cover its entire structure, we could model this based on the good-quality density for Ub^D^ and the available cocrystal structure of RING-E2~Ub^D^ (*52*) among others, as well as the cryo-EM structure of E2 bound to full CRL, which guided positioning relative to NEDD8 and the C-terminal region of the Cullin (*30*), giving us confidence on the positioning of the key catalytic E2~Ub^D^ unit in the model.

As no structure has been determined for UBE2R1/2 E2 enzymes bound to any E3 ligase, we used protein-observed NMR spectroscopy (*63*) to determine how UBE2R1 interacts with Ub^D^ in solution in the context of the UBE2R1~Ub conjugate. Analysis of the ^15^N-^1^H HSQC NMR spectrum of ^15^N-labeled Ub^D^ loaded on UBE2R1(C93K) showed large chemical shift perturbations and signal attenuation, corresponding to clear stabilization of Ub^D^ on the E2, in particular through the canonical hydrophobic patch including residues L8, I44, and V70 (fig. S13). Crucially, we observe that this hydrophobic patch surface is fully buried in contact with UBE2R1 also in our cryo-EM structure, evidencing the UBE2R1~Ub bound in a closed, precatalytic state (Fig. 4B, bottom middle). Several interactions between the E2 and Rbx1 were observed as expected. Nonetheless, the modeled Asp^102^ and Asp^103^ residues on the acidic loop of UBE2R1 were found close to key basic residues on Rbx1 with which they are known to interact, such as Arg^91^ (*64*). Previous work has shown that the UBE2R acidic tail binds a basic “canyon” on Cul1 that is highly conserved throughout all Cullins (*54*). We could not identify volume for the acidic region of UBE2R1 in our cryo-EM structure, hence could not confidently model this. To further validate interactions in this highly dynamic RING-E2-Ub^D^ region of our cryo-EM structure, we designed a ubiquitin-directed photoreactive probe (UDPRP) and used it in cross-linking MS experiments. We synthesized the photocrosslinker *N*-maleimido-diazirine (see Materials and Methods and fig. S15) that would site-specifically react with E34C mutant ubiquitin, chosen based on its proximity to Rbx1 in existing structures (*30*), and formed a stable and active isopeptide-linked Ub-UBE2R1 conjugate via the E2 C93K (see fig. S15, A to E). In the photocrosslinking assay containing neddylated-CRL2^VHL^, the UDPRP successfully photocrosslinked to Rbx1 (fig. S15, F and G). The photocrosslinked product was excised from the gel and analyzed by MS to identify insertion in the ^106^YGH^108^ sequence at the flexible C terminus of Rbx1 (fig. S15, H and I). While density was not visible for the terminal YGH tail of Rbx1 in our structure, the distance of ~18 Å between Ub^D^(E34C) and K105 right upstream of YGH in Rbx1 is consistent with positioning of the cross-linker–YGH (Fig. 4B, bottom middle). Together, our complementary structural NMR and cross-linking studies in solution validate our models of the E2~Ub:Rbx1 components in the cryo-EM structure volume.

Given the Ub^A^ species was absent from the cryo-EM volume, we did not capture the process of ubiquitin chain elongation. Instead, we observed an increased occupancy of the E2 bound to the E3 and a closing of the CRL ring. While UBE2R1 is known to be proficient at ubiquitin chain elongation, our cryo-EM structure revealed potential interactions at the *neo*-substrate:E2-Ub^D^ interface, suggesting a predisposition for UBE2R1 to install a first ubiquitin on Brd4^BD2^. To gain structural insights into preferences for acceptor lysine on the *neo*-substrate, we closely inspected the interface between UBE2R1 and Brd4^BD2^, and measured distances with the ubiquitinable lysine residues of Brd4 (Fig. 4B, bottom right, and fig. S16). While Brd4^BD2^ and UBE2R1 are not in direct contact, nor do they show any protein-protein interactions, we observed that K456, which we had found as preferentially ubiquitinated, points directly toward the E2~Ub electrophilic site (Fig. 4B, bottom right). We measured a distance of 18.4 Å between the N-epsilon of K456 and the carbonyl of the modeled thioester at C93 (Fig. 4B, bottom right, and fig. S16). Albeit not at the shortest distance, it appears that K456 is primed for an optimal geometry to approach the electrophilic thioester on the E2~Ub^D^. As K456 is located at the C-terminal end of the C helix of the bromodomain, distal to the bound MZ1 interface, the shown tilt could help to bridge such a distance. Residues K368 and K445 are both also in close proximity to the active site C93. Together, our structural work exploited cross-linking stabilization to achieve a high-resolution cryo-EM structure for a fully closed (NEDD8)-CRL2^VHL^-PROTAC-target-Ub^A^-E2~Ub^D^ structure that we validate using an in-solution technique and that allowed to gain insights into *neo*-substrate ubiquitination as catalyzed by a PROTAC-bound CRL machinery.

**Fig. 5.**
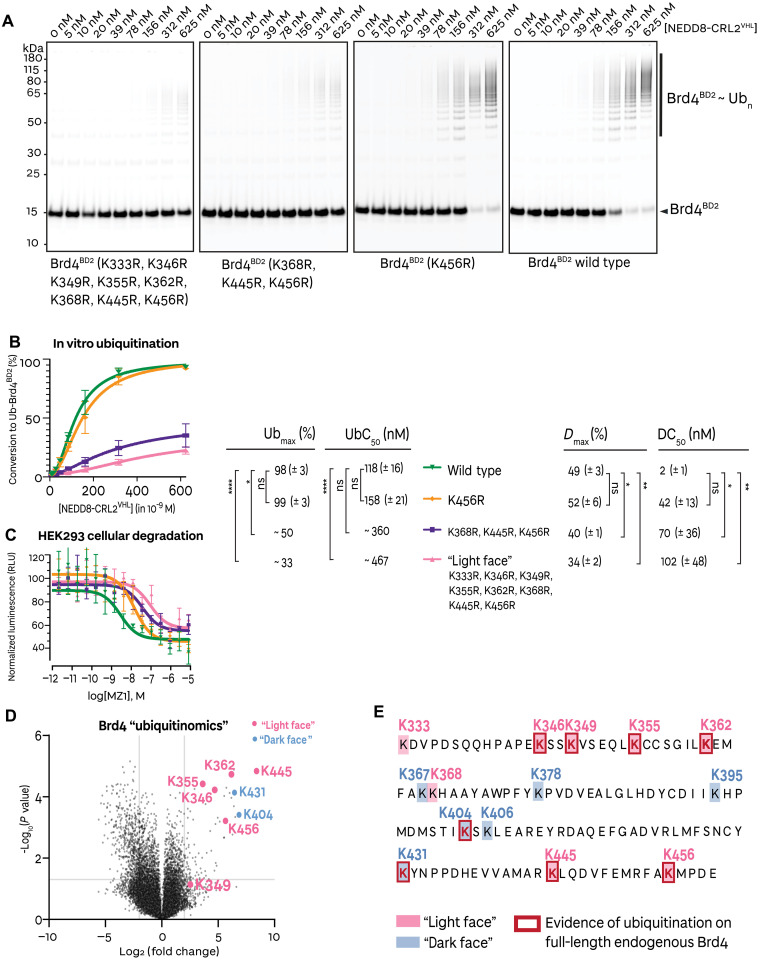
Brd4^BD2^ lysine mutants illustrate specificity in ubiquitinability and degradability. (**A**) Representative in vitro ubiquitination assay with Alexa Fluor 647 (AF647)–labeled recombinantly expressed and purified Brd4^BD2^ mutants, performed in the presence of (NEDD8)-CRL2^VHL^, MZ1, and UBE2R1. The species were resolved by SDS-PAGE and scanned at 647 nm to visualize unmodified Brd4^BD2^ and ubiquitinated Brd4^BD2^ species. (**B**) Quantification of the percent conversion from unmodified Brd4^BD2^ to ubiquitinated Brd4^BD2^ species from the in vitro ubiquitination assay. The error bars represent the mean of *N* = 3 replicates with SEM. UbC_50_ and Ub_max_ values are calculated. (**C**) MZ1 dose-dependent cellular degradation of NanoLuc-Brd4^BD2^ wild-type and lysine mutants. The error bars represent the mean of *N* = 4 replicates with SEM. DC_50_ and *D*_max_ values are calculated. (**D**) Global changes in the cellular “ubiquitinome,” as assessed by di-glycine remnant mass spectrometry profiling on HEK293 cells following treatment with 1 μM MZ1 for 15 min. The depicted volcano plot shows 25,843 ubiquitinated precursors presented as log2 fold change relative to DMSO controls. The positions of light face lysines, which showed evidence of modification in vitro, are highlighted in pink, while dark face lysines, which showed no evidence of modification in vitro but are found ubiquitinated in cells, are highlighted in blue. (**E**) Sequence of Brd4^BD2^ overlaying the results of in vitro ubiquitination and ubiquitinomics from this study. The legend denotes light face lysines (highlighted in pink; evidence of ubiquitination in vitro on recombinant Brd4^BD2^), dark face lysines (highlighted in blue; no evidence of ubiquitination in vitro on recombinant Brd4^BD2^), and lysines that showed evidence of modification from cellular ubiquitinomics experiments (red bold font and red box).

Our MS of the in vitro ubiquitination reaction and structural inspection of our cryo-EM structures together indicated a preference for a specific patch of three lysines (K368, K445, and K456) in close proximity to the E2’s active site cysteine for ubiquitination to occur. To understand the importance of these lysines, we performed in vitro ubiquitination and cellular degradation assays with wild-type and Lys-to-Arg mutant Brd4^BD2^ constructs (Fig. 5, A to C). To assess these results, we establish Ub_max_ as the maximal conversion from nonubiquitinated to ubiquitinated substrate, and UbC_50_ as the half-maximal ubiquitination concentration. The differences in Ub_max_ and UbC_50_ between the wild-type and single K456R mutant were negligible, as often observed in Cullin RING ligase substrate reactions. In contrast, the triple “ubiquitination zone” mutant significantly impaired ubiquitination, to a similar extent as the full light face mutant, both yielding statistically significant (unpaired two-tailed *t* test) differences in Ub_max_ compared to wild type (Fig. 5B and fig. S19B). Similarly, the difference in *D*_max_ and DC_50_ from wild type relative to the single-, triple- and eight-point mutants followed similar trends and significance, showing the impact in ubiquitinability translating directly in impact into degradability (Fig. 5C and fig. S19C). Together, our ubiquitination and cellular degradation data show that the single K456R mutant had comparable ubiquitination and degradation relative to wild-type Brd4^BD2^, likely owing to the longevity of the VHL-MZ1-Brd4^BD2^ combined with the flexibility of the CRL system allowing to buffer against the loss of this acceptor Lys^456^ and allowing for other lysines to be captured. Nonetheless, when coupled with additional mutations of two adjacent lysines as a ubiquitination zone three-point mutant, this was sufficient to impair ubiquitinability and degradability of the Brd4 bromodomain substrate largely to the same extent as an eight-point mutant corresponding to the entire light face on Brd4^BD2^.

Finally, we were curious to assess whether the identified ubiquitinatable lysines could be detected in a cellular context on the endogenous protein. To this end, we set out to perform a cellular “ubiquitinomics” experiment of MZ1-treated cells to analyze ubiquitination on the full endogenous Brd4 (and other BET proteins Brd2 and Brd3). Following the short treatment of human embryonic kidney (HEK) 293 cells with 1 μM MZ1, we carried out quantitative di-glycine remnant profiling by MS and identified 25,843 ubiquitinated precursors corresponding to 5117 proteins (Fig. 5D and fig. S20). From these, we identified several lysine residues of Brd4, as well as Brd2 and Brd3 with notably higher ubiquitination upon MZ1 treatment compared to dimethyl sulfoxide (DMSO) (fig. S22). We identified six of eight lysine residues on the light face of Brd4^BD2^ as ubiquitinated in cell, including both K445 and K456 from the specific ubiquitination zone (Fig. 5E and fig. S21). In agreement with our data, the same two lysines (K445 and K456) were also identified as MZ1-dependent ubiquitination sites from mass spectrometric analysis of endogenously immunoprecipitated Brd4 in a previous study (*65*). In contrast, only 2 (K404 and K431) of the 6 lysine residues of the dark face were identified as ubiquitinatable in cell, yielding a total of 8 of 14 residues (~57%) detected as ubiquitinated on Brd4^BD2^ (Fig. 5, D and E, and figs. S20 to S22). In comparison, the corresponding Brd2^BD2^ and Brd3^BD2^ were found ubiquitinated at 3 of 15 (20%) and 5 of 13 (38%) lysine residues, respectively, hence at fewer sites compared to Brd4^BD2^ (fig. S21). Together, our in vitro ubiquitination data with recombinant bromodomain and the cellular degradation and ubiquitinomics results collectively are consistent with the degrader MZ1 orchestrating specific ubiquitinability of Lys residues on the light face of its preferred substrate Brd4^BD2^, as directed by the specific structural and biophysical characteristics of the ternary complex.

## DISCUSSION

Degrader drugs co-opt the catalytic activity of ubiquitin E3 Cullin RING ligases to drive efficient ubiquitination and degradation of disease-causing proteins (*5*, *6*). Catalytic activity and substrate specificity of CRLs are critically determined by the spatial organization and relative interactions of their subunits and by their ability to work in concert to achieve flexibility (*30*, *56*). These are important requirements not only to bring together a substrate and ubiquitin that would otherwise be too far apart, but also to allow hitting the substrate at multiple positions and to accommodate the building of ubiquitin chains during the catalytic cycles (*58*). Here, our objective was to determine the structural and mechanistic bases of how optimal degraders mediate productive target ubiquitination. This information is required to establish the degradability or PROTACability of targets, for which rules have remained unclear (*66*, *67*). By combining cryo-EM structures, cross-linking trapping strategies, and biochemical identification of ubiquitinated lysines in vitro and in cell, we determine the structure and mechanism of the entire PROTAC-induced Cullin 2 RING ligase VHL catalytic machinery, and how it operates to catalyze ubiquitination on its highly specific *neo*-substrate—the second bromodomain of Brd4—that is recruited to VHL by the PROTAC MZ1. Of note, analogous structures were also recently released by Liwocha *et al.* (*68*) and Li *et al.* (*69*) in their studies of ubiquitin chain initiation and elongation by UBE2R2, using a K368C–cross-linked Brd4^BD2^ species in complex with MZ1 and CRL2^VHL^. In brief, while the overall architecture of these structures was in good agreement with our data, exact comparisons could not be drawn in terms of lysine modification owing to the different E2s used and the rigid cross-linking strategy used for trapping of K368 in proximity to the E2 active-site cysteine (*68*, *69*).

**Fig. 6.**
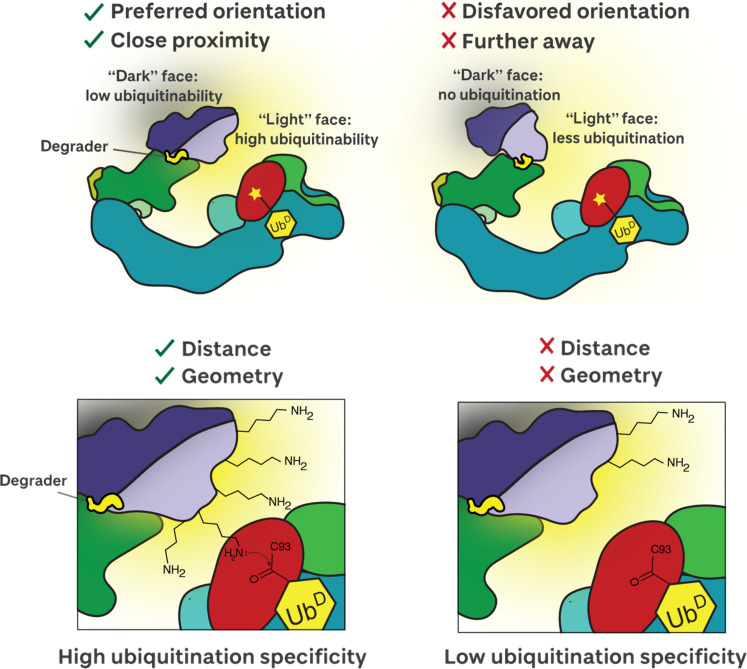
Schematic of structural and mechanistic feature of ubiquitinability of target proteins revealed from this study.

Our results explain the “ubiquitinability” of Brd4^BD2^ and allow us to establish general principles regarding the structural requirements for *neo*-substrate ubiquitination specificity (Fig. 6). We show that Brd4^BD2^, once tightly glued to VHL by MZ1, adopts a preferred orientation projecting toward the RING activated E2-ubiquitin catalytic module of the CRL. This well-defined geometry projects one face of the substrate toward the E2, such that lysine residues on this surface are within range of reacting with the Ub~E2 thioester, i.e., the ubiquitination zone (*33*), and are thus susceptible to ubiquitin modification. Preferentially ubiquitinated residues are on a light face, as though they would be illuminated by a source of light projecting from the E2~Ub^D^ (Fig. 6, top left). This suggests that if a lysine residue is present on the light face of the target protein, it has greater potential for ubiquitin modification. In contrast, lysine residues present on the opposite dark face of the protein are less likely to be ubiquitinated. The arrangement of “light” and “dark” faces on a substrate will be determined by its orientation relative to the E3 ligase as dictated by the degrader. Tight, stably bound ternary complexes are necessary for fast, potent protein degradation (*13*, *29*, *34*–*39*). Here, we show that depending on the geometry of such stable complex, different light faces could arise on the target protein. For a given E3 ligase, varying either of the other two components within the ternary complex could lead to very different orientations of the target protein relative to the E2~Ub, different light faces, and so different degrees of ubiquitinability. Examples of this include when varying chemical structures of PROTACs for the same target, as done in drug discovery optimization projects, as well as for a given degrader against different target mutants, isoforms, or paralogs, as is the case here for MZ1 with the various BET bromodomains (*13*, *29*). Recruiting the substrate in a disfavored orientation, pointing away from the E2~Ub site, would result in less efficient ubiquitination, and hence less effective degraders (Fig. 6, top right).

Our data together are also consistent with the notion that, even within a highly favored system such as VHL-MZ1-Brd4^BD2^, which bears several lysine residues on the light face, not all lysine residues will be equal as their specific position, geometry, and reactivity will all be factors in determining their efficiency for ubiquitination. We find that a single lysine (K456) on the light face of Brd4^BD2^ is optimally positioned for nucleophilic attack on the E2-Ub on our cryo-EM structure, is preferentially ubiquitinated by CRL2^VHL^ in vitro, and is detected as a ubiquitinated site on the endogenous protein in cell. While a single K456R mutation did not markedly affect substrate ubiquitination, it substantially did so when complemented with additional mutations of two adjacent lysines. Thus, to achieve fast, potent, and profound target degradation, the degrader must recruit the target not only in a stable, long-lived complex at low concentration, but also orienting it favorably toward the catalytic site. In addition to this, our work shows that it is also important that one or more lysine residues within the light face are properly positioned with respect to both distance and geometry to drive highly specific and efficient ubiquitination (Fig. 6, bottom). In addition, by cryo-EM, we resolve the inherent conformational heterogeneity of the cullin RING complex. Our data show that on loss of a single lysine (K456R), the longevity of the VHL-MZ1-BET complex coupled with the flexibility of the CRL can buffer against geometric preferences, and allow for suboptimal lysines to be captured, albeit less effectively.

Together, the fresh mechanistic insights unveiled in this work advance our knowledge of degraders’ mode of action, further explain the intra-BET specificity of the archetypical PROTAC MZ1, and reveal guiding principles for understanding and manipulating the exquisite degradation specificity of PROTACs and molecular glues. While this work has focused on CRL2^VHL^ as E3 ligase and Brd4 as target protein of interest, these conclusions are expected to be general and apply to other E3 ligases, e.g., CRL4^CRBN^ and beyond, and to other proteins that are targeted for degradation (*70*). We therefore anticipate that our findings will inspire further in-depth mechanistic studies and will guide more refined structure-based rational optimization of improved degrader drugs.

## MATERIALS AND METHODS

### Protein expression and purification

#### VCB

VCB (VHL residues 54 to 213, EloB residues 1 to 104, and EloC residues 17 to 112) was expressed and purified as previously described. In short, single colonies of cDNA transformed into *Escherichia coli* BL21(DE3) cells were grown overnight in lysogeny broth (LB) supplemented with ampicillin (100 μg ml^−1^) and streptomycin (50 μg ml^−1^) at 37°C with shaking. The overnight culture was diluted 1:100 in LB supplemented with ampicillin (100 μg ml^−1^) and streptomycin (50 μg ml^−1^) and grown to an optical density (OD_600_) of ~0.6. Protein expression was induced overnight at 18°C with 0.3 mM isopropyl β-d-1-thiogalactopyranoside (IPTG). The cells were harvested by centrifugation and frozen at −80°C as pellets until further purification. The bacterial pellets were resuspended in buffer and lysed by cell disruption using One Shot Cell Disruptor (Constant Systems) operating at 4°C. Cellular debris was removed by centrifugation. His_6_-tagged proteins were purified on a HisTrap FF Ni NTA affinity column (Cytiva) and eluted with an imidazole concentration gradient. The protein was dialyzed into a low-concentration imidazole buffer, incubated with Tobacco Etch Virus (TEV) protease, and flowed through a HisTrap FF Ni NTA affinity column. VCB was additionally purified by anion exchange using a HiTrap Q HP column. The protein sample was finally purified by size exclusion chromatography on a HiLoad 16/600 Superdex 75 pg column (Cytiva) in 20 mM Hepes, pH 7.5, 150 mM sodium chloride, and 0.5 mM TCEP, concentrated, flash frozen with liquid nitrogen, and stored at −80°C.

#### NEDD8 (residues 1 to 76)

Single colonies of cDNA transformed into *E. coli* BL21(DE3) cells were grown overnight in LB supplemented with ampicillin (100 μg ml^−1^) at 37°C with shaking. The overnight culture was diluted 1:100 in 9 liters of LB supplemented with ampicillin (100 μg ml^−1^) and grown to an optical density (OD_600_) of ~0.6. Protein expression was induced overnight at 16°C with 0.5 mM IPTG. The cells were harvested by centrifugation and frozen at −80°C as pellets until further purification. The bacterial pellets were resuspended in 30 mM tris, 200 mM NaCl, and 5 mM dithiothreitol (DTT), pH 7.5, supplemented with 5 mM MgCl_2_, DNase I (1 μg/ml), and 1× EDTA-free Roche protease inhibitor cocktail, and lysed by cell disruption using One Shot Cell Disruptor (Constant Systems) operating at 4°C. Cellular debris was removed by centrifugation. The His_6_-tagged protein was purified on a HisTrap FF Ni NTA affinity column (Cytiva) and eluted with an imidazole concentration gradient (20 to 500 mM). The resulting eluate was incubated with 10 ml of glutathione Sepharose 4B resin (Cytiva). The protein-bound resin was washed with 50 mM tris-HCl, 200 mM NaCl, and 5 mM DTT, pH 7.6. TEV was added to the resin, and NEDD8 was cleaved at room temperature for 3 hours. NEDD8 was finally purified by size exclusion chromatography on a HiLoad 16/600 Superdex 75 pg column (Cytiva) in 20 mM Hepes, pH 7.5, 150 mM sodium chloride, and 0.5 mM TCEP, concentrated, flash frozen with liquid nitrogen, and stored at −80°C.

#### APPBP1-UBA3 and UBE2M

Single colonies of cDNA (*71*, *72*) transformed into *E. coli* BL21(DE3) cells were grown overnight in LB supplemented with ampicillin (100 μg ml^−1^) at 37°C with shaking. The overnight culture was diluted 1:100 in LB supplemented with ampicillin (100 μg ml^−1^) and grown to an optical density (OD_600_) of ~0.6. Protein expression was induced overnight at 16°C with 0.5 mM IPTG. The cells were harvested by centrifugation and frozen at −80°C as pellets until further purification. The bacterial pellets were resuspended in 50 mM tris, 200 mM NaCl, and 5 mM DTT, pH 7.5, supplemented with 5 mM MgCl_2_, DNase I (1 μg/ml), and 1× EDTA-free Roche protease inhibitor cocktail, and lysed by cell disruption using One Shot Cell Disruptor (Constant Systems) operating at 4°C. Cellular debris was removed by centrifugation. The lysate was incubated with 10 ml of glutathione Sepharose 4B (Cytiva) resin for 1 hour at room temperature. The resin was washed with 50 mM tris-HCl, 200 mM NaCl, and 5 mM DTT, pH 7.6, and the protein was eluted with 50 mM tris, 200 mM NaCl, 5 mM DTT, pH 8.0, and 10 mM reduced L-glutathione. One unit of thrombin per milligram of total protein was added and the mixture was incubated overnight at −4°C. APPBP1-UBA3 was further purified by size exclusion chromatography on a HiLoad 16/600 Superdex 200 pg column (Cytiva) in 20 mM Hepes, pH 7.5, 150 mM sodium chloride, and 0.5 mM TCEP. UBE2M was further purified by size exclusion chromatography on a HiLoad 16/600 Superdex 75 pg column (Cytiva) in 20 mM Hepes, pH 7.5, 150 mM sodium chloride, and 0.5 mM TCEP. The resulting proteins were flowed over glutathione Sepharose 4B resin to remove any trace amounts of GST-APPBP1-UBA3, GST-UBE2M, and GST. The purified protein samples were each concentrated, flash frozen with liquid nitrogen, and stored at −80°C.

#### NEDD8-CRL2^VHL^

Single colonies of Cul2-Rbx1 cDNA (*73*) transformed into *E. coli* BL21(DE3) cells were grown overnight in LB supplemented with ampicillin (100 μg ml^−1^) at 37°C with shaking. The overnight culture was diluted 1:100 in LB supplemented with ampicillin (100 μg ml^−1^) and grown to an optical density (OD_600_) of ~0.6. Protein expression was induced for 16 hours at 16°C with 0.2 mM IPTG. The cells were harvested by centrifugation and frozen at −80°C as pellets until further purification. The cells were thawed, resuspended in 30 mM tris-HCl, 200 mM NaCl, and 5 mM DTT, pH 7.5, supplemented with 5 mM MgCl_2_, DNase I (1 μg/ml), and 1× EDTA-free Roche protease inhibitor cocktail, and lysed by cell disruption at 30 kpsi. Cellular debris was removed by centrifugation. Cul2-Rbx1 was purified on a 5-ml HisTrap FF Ni NTA affinity column (Cytiva) and eluted with an imidazole concentration gradient from 0 to 300 mM. The protein was desalted into 20 mM Hepes, 150 mM NaCl, 0.5 mM TCEP, and 5% (v/v) glycerol, pH 7.5, with a HiPrep 26/10 desalting column (Cytiva) and incubated with TEV protease and an excess of recombinant VHL-EloB-EloC. VHL-EloB-EloC-Cul2-Rbx1 (CRL2^VHL^) was purified on a 5-ml StrepTrap XT column, washed with 100 mM tris-HCl and 150 mM NaCl, pH 8.0, and eluted in 100 mM tris-HCl, 300 mM NaCl, 50 mM biotin, and 5% (v/v) glycerol, pH 8.0. Purified CRL2^VHL^ (1.7 μM) was incubated with Uba3-APP-BP1 (250 nM), Ube2M (1.2 μM), NEDD8 (20 μM), adenosine triphosphate (ATP) (1 mM), and MgCl_2_ (5 mM) for 10 min at 37°C. NEDD8-CRL2^VHL^ was finally purified by size exclusion chromatography on a HiLoad 16/600 Superdex 200 pg column (Cytiva) in 20 mM Hepes, 150 mM NaCl, 0.5 mM TCEP, and 5% (v/v) glycerol, pH 7.5, concentrated, flash frozen with liquid nitrogen, and stored at −80°C.

#### GACG-ubiquitin

A single colony of SoluBL21(DE3) competent cells (Genlantis) encoding for His_6_-TEV-GACG-ubiquitin (*74*) was grown overnight in LB supplemented with kanamycin (50 μg ml^−1^) at 37°C with shaking. The overnight culture was diluted 1:100 in 6 liters of LB supplemented with kanamycin (50 μg ml^−1^) and grown to an optical density (OD_600_) of ~0.75. Protein expression was induced for 4 hours at 37°C with 1 mM IPTG. The cells were harvested by centrifugation and frozen at −80°C as pellets until further purification. The cells were thawed, resuspended in 20 mM Hepes, 500 mM NaCl, and 0.5 mM TCEP, pH 7.5, supplemented with 5 mM MgCl_2_, DNase I (1 μg/ml), and 1X EDTA-free Roche protease inhibitor cocktail, and lysed by cell disruption at 30 kpsi using One Shot Cell Disruptor (Constant Systems) operating at 4°C. Cellular debris was removed by centrifugation. His_6_-TEV-GACG-ubiquitin was purified on a HisTrap FF Ni NTA affinity column (Cytiva) and eluted with an imidazole concentration gradient from 0 to 500 mM. The protein was dialyzed into 50 mM Hepes, 150 mM NaCl, and 0.5 mM TCEP, pH 7.5, incubated with TEV protease, and flowed through a HisTrap FF Ni NTA affinity column. GACG-ubiquitin was finally purified by size exclusion chromatography on a HiLoad 16/600 Superdex 75 pg column (Cytiva) in 50 mM tris-HCl, 150 mM NaCl, and 0.5 mM TCEP, pH 7.5, concentrated, flash frozen with liquid nitrogen, and stored at −80°C.

#### ^15^N-labeled ubiquitin

^15^N-labeled ubiquitin was prepared using N-terminal 6×His-TEV on human wild-type Ub residues 1 to 76, referred to as 6×His-TEV-Ub. The construct transformed into *E. coli* BL21(DE3) Rosetta II (Novagen) and ^15^N-6×His-TEV-Ub was expressed in autoinducing NPS buffer with ^15^N-ammonium chloride (Cambridge Isotope Laboratories) as the only source of nitrogen following (*75*). ^15^N-NPS autoinducing media (2 liters) was grown at 37°C for 20 hours, and the cells were harvested, resuspended in buffer A (20 mM sodium phosphate, 500 mM NaCl, 50 mM imidazole, and 1 mM TCEP, pH 7.4), and lysed at 20 kpsi using One Shot Cell Disruptor (Constant Systems) operating at 4°C. The lysate was cleared by centrifugation at 19,000 rpm for 30 min, passed through a 0.45-μm syringe filter, loaded onto an equilibrated 5-ml His-Trap HP column, washed for 30 CV, and eluted with 100% buffer B (20 mM sodium phosphate, 500 mM NaCl, 350 mM imidazole, and 1 mM TCEP, pH 7.4). This was followed by dialysis in phosphate-buffered saline (PBS) buffer with TEV protease overnight at 18°C. The reaction was concentrated and loaded on a 16/600 Superdex 75 column (GE Life Sciences) in PBS. The expected peak for ^15^N-Ub was observed around 0.76 Column Volumes (CV), and its purity was confirmed with SDS-PAGE. Fractions containing pure ^15^N-Ub were concentrated using a 3500 Da centrifugal filter unit (Merck-Millipore) and exchanged into NMR buffer (20 mM sodium phosphate, 150 mM NaCl, and 0.5 mM TCEP, pH 7.0).

#### Brd4^BD2^

Brd4^BD2^ (residues 333 to 460), wild type and lysine to arginine mutants, were expressed and purified as previously described (*13*). In short, single colonies of cDNA transformed into *E. coli* BL21(DE3) cells were grown overnight in LB supplemented with kanamycin (50 μg ml^−1^) at 37°C with shaking. The overnight culture was diluted 1:100 in LB supplemented with kanamycin (50 μg ml^−1^) and grown to an optical density (OD_600_) of ~0.6. Protein expression was induced overnight at 18°C with IPTG (0.3 mM). The cells were harvested by centrifugation and frozen at −80°C as pellets until further purification. The bacterial pellets were resuspended in buffer and lysed by cell disruption using One Shot Cell Disruptor (Constant Systems) operating at 4°C. Cellular debris was removed by centrifugation. The His_6_-tagged protein was purified on a HisTrap FF Ni NTA affinity column (Cytiva) and eluted with an imidazole concentration gradient of 0 to 500 mM. The protein was dialyzed into a low-concentration imidazole buffer, incubated with TEV protease, and flowed through a HisTrap FF Ni NTA affinity column. Brd4^BD2^ was finally purified by size exclusion chromatography on a HiLoad 16/600 Superdex 75 pg column (Cytiva) in 20 mM Hepes, pH 7.5, 150 mM sodium chloride, and 0.5 mM TCEP, concentrated, flash frozen with liquid nitrogen, and stored at −80°C.

#### ^15^N-labeled Brd4^BD2^

^15^N-labeled Brd4^BD2^ was obtained as a His-GST-TEV-Brd4(349 to 459) pGEX4T1 construct. Expression and purification were carried as ^15^N-6×His-TEV-Ub (above). Following the 16/60 Superdex 75 column (GE Life Sciences), step pure fractions of ^15^N-Brd4^BD2^ were exchanged into NMR buffer using a 3500 Da centrifugal filter unit (Merck-Millipore).

#### Ub^K48C,G76S^-Brd4^BD2^

Ub^K48C,G76S^-Brd4^BD2^ variant for thiol cross-linking was prepared from an N-terminal 6×His-TEV-Ub^K48C,G76S^-Brd4(349 to 459) “Ub^K48C,G76S^-Brd4^BD2^” construct in pRSF-Duet1 in *E. coli* BL21(DE3) Rosetta II (Novagen). Cultures of LB supplemented with kanamycin (50 μg ml^−1^) were grown at 37°C to an optical density (OD_600_) of ~0.8, the temperature was lowered 16°C and expressed with 0.5 mM IPTG overnight. Cells were harvested by centrifugation, resuspended in IMAC buffer A (20 mM sodium phosphate, 500 mM NaCl, 50 mM imidazole, and 1 mM TCEP, pH 7.4), supplemented with 5 mM MgCl_2_, and DNase I (1 μg/ml). Cells were lysed at 20 kpsi using One Shot Cell Disruptor (Constant Systems) operating at 4°C. The lysate was cleared by centrifugation at 19,000 rpm for 30 min, passed through a 0.45-μm syringe filter, loaded onto an equilibrated 5 ml His-Trap HP column, washed for 25 CV in IMAC Buffer A, and eluted with 100% IMAC buffer B (20 mM sodium phosphate, 500 mM NaCl, 350 mM imidazole, and 1 mM TCEP, pH 7.4). This was followed by dialysis in PBS buffer with TEV protease overnight at 18°C. The reaction passed through a fresh 10-ml His-Trap HP column to remove TEV protease and uncleaved protein. This was concentrated using a 10,000-Da molecular weight cutoff (MWCO) centrifugal filter unit (Merck-Millipore) and loaded on a 16/600 Superdex 75 column (GE Life Sciences) in cross-linking buffer (20 mM Hepes, 150 mM NaCl, and 0.5 mM TCEP, pH 7.0). The purity of Ub^K48C,G76S^-Brd4^BD2^ was confirmed by SDS-PAGE and the protein was stored at −80°C until use.

#### UBE2R1(C93K)

UBE2R1(C93K) was prepared as a His-SUMO fusion of the full-length human UBE2R1 in pET28b. Expression was carried out in *E. coli* BL21(DE3) Rosetta II (Novagen) cells growing in LB supplemented with kanamycin (50 μg ml^−1^). Cells were grown to an optical density (OD_600_) of ~0.8 at 37°C, and the temperature was adjusted to 16°C and induced with 0.35 mM IPTG overnight. Cells were harvested by centrifugation, resuspended in IMAC buffer A (20 mM sodium phosphate, 500 mM NaCl, 50 mM imidazole, and 1 mM TCEP, pH 7.4), and supplemented with 5 mM MgCl_2_ and DNase I (1 μg/ml). Cells were lysed at 20 kpsi using One Shot Cell Disruptor (Constant Systems) operating at 4°C. The lysate was cleared by centrifugation at 19,000 rpm for 30 min, passed through a 0.45-μm syringe filter, loaded onto an equilibrated 5-ml His-Trap HP column, washed for 25 CV in IMAC Buffer A, and eluted with 100% IMAC buffer B (20 mM sodium phosphate, 500 mM NaCl, 350 mM imidazole, and 1 mM TCEP, pH 7.4). This was followed by dialysis in 20 mM Hepes, 150 mM NaCl, and 1 mM TCEP (pH 7.5) buffer with 2 μM ULP1 overnight at 4°C. The reaction passed through a fresh 10-ml His-Trap HP column to remove ULP1 protease and the His-SUMO tag. This was concentrated using a 10,000-Da MWCO centrifugal filter unit (Merck-Millipore) and loaded on a 16/600 Superdex 75 column (GE Life Sciences) in 20 mM Hepes, 150 mM NaCl, and 1 mM TCEP, pH 7.5. The purity of UBE2R1^C93K^ was confirmed by SDS-PAGE, and the protein was concentrated to a 1 mM stock, flash frozen, and stored at −80°C until use. The UBE2R1^C92K,S138C, C191S,C223S^ variant used for chemical cross-linking was prepared following the same method, with the only exception being the buffer for the final 16/600 Superdex 75 column (GE Life Sciences) was 20 mM Hepes, 150 mM NaCl, and 0.5 mM TCEP, pH 7.0.

#### Ubiquitin-activating enzyme 1

Ubiquitin-activating enzyme 1 (UBA1) was expressed following (*76*) to obtain the full-length human UBA1 with C-terminal 6×His in pRSF-Duet1. *E. coli* BL21(DE3) Rosetta II (Novagen) cells were grown at 37°C in LB supplemented with kanamycin (50 μg ml^−1^) and 1 mM MgSO_4_ to an optical density (OD_600_) of ~0.8. The temperature was adjusted to 16°C, IPTG was added to 0.2 mM, and induction proceeded overnight. Cells were harvested by centrifugation, resuspended in IMAC buffer A (20 mM sodium phosphate, 500 mM NaCl, 50 mM imidazole, and 1 mM TCEP, pH 7.4), and supplemented 5 mM MgCl_2_ and DNase I (1 μg/ml). Lysis was performed at 20 kpsi using One Shot Cell Disruptor (Constant Systems) operating at 4°C. The lysate was cleared by centrifugation at 19,000 rpm for 30 min, passed through a 0.45-μm syringe filter, loaded onto an equilibrated 5 ml His-Trap HP column, washed for 25 CV in IMAC Buffer A, and eluted with 100% IMAC Buffer B (20 mM sodium phosphate, 500 mM NaCl, 350 mM imidazole, and 1 mM TCEP, pH 7.4). The elution was concentrated to ~2 ml using a 30,000-Da MWCO centrifugal filter unit (Merck-Millipore) and diluted 1:100 in Anion Buffer A (50 mM tris and 5 mM 2-mercaptoethanol, pH 8.0) and loaded on an equilibrated 5-ml Q-HP column (Cytiva). A 50% gradient over 40 CV with Anion Buffer B (50 mM tris, 1 M NaCl, and 5 mM 2-mercaptoethanol, pH 8.0) was applied and SDS-PAGE showed UBA1 eluted in the first major peak at ~18% buffer B. These fractions were concentrated and loaded onto a final 16/60 Superdex 200 column (Cytiva) equilibrated in 20 mM Hepes, 150 mM NaCl, and 1 mM TCEP, pH 8.0. Pure UBA1 was concentrated to 100 μM and stored at −80°C until use.

#### Ubiquitin(E34C) and UBE2R1(C93K) (His construct)

Both were prepared using an N-terminal His_6_-TEV cleavable tag. Single colonies transformed into *E. coli* BL21 (DE3) were grown overnight in LB supplemented with kanamycin (50 μg/ml) at 37°C with shaking at 220 rpm. The overnight culture was diluted 1:100 in 0.5 liters of LB supplemented with kanamycin (50 μg/ml) and grown to an optical density (OD_600_) between 0.6 and 0.8 at 37°C with shaking at 220 rpm. IPTG was added to a final concentration of 0.1 mM to induce protein expression and cultures were incubated at 18°C with shaking at 220 rpm for 18 hours. Bacterial cells were pelleted by centrifugation (6500 rpm/20 min/4°C) and resuspended in 50 mM tris, 150 mM NaCl, 10 mM imidazole, and complete protease inhibitor cocktail (EDTA-free, Roche), pH 7.5. The cell suspension was either flash frozen in liquid nitrogen and stored at −80°C until further use, or purification was carried out straight away. The bacterial cells were lysed by cell disruption (Avestin) and centrifuged (27,200*g*/45 min/4°C) to remove any insoluble material. The supernatant was filtered through a 0.2 μM filter and loaded onto a Ni-NTA agarose (Qiagen) column (1 ml of beads per 0.5 liters of bacterial culture) preequilibrated with 50 mM tris, 150 mM NaCl, and 10 mM imidazole, pH 7.5. The column was washed successively with 50 mM tris, 150 mM NaCl, and 10 mM imidazole, pH 7.5 (~8 column volumes) and 50 mM tris, 150 mM NaCl, and 30 mM imidazole, pH 7.5 (~6–8 column volumes) and eluted with 50 mM tris, 150 mM NaCl, 150 mM imidazole, and 0.5 mM TCEP, pH 7.5. Fractions containing His_6_-Ub/UBE2R1 C93K were pooled, and TEV protease was added [1 mg TEV:50 mg (Ub/UBE2R1)] and dialyzed for 18 hours at 4°C against 50 mM tris, 150 mM NaCl, and 0.5 mM TCEP, pH 7.5. Once most of the protein was cleaved, imidazole was added to the final concentration of 10 mM and passed through the Ni-NTA agarose column preequilibrated with 50 mM tris, 150 mM NaCl, 10 mM imidazole, and 0.5 mM TCEP, pH 7.5. The flow-through fraction was collected, concentrated to between 5 and 10 mg/ml using a Vivaspin centrifugal concentrator (Sartorius) (3 kDa MWCO, 3000*g*, 4°C), flash frozen in liquid nitrogen, and stored at −80°C.

### Ubiquitin labeling with maleimide Alexa Fluor 488

Alexa Fluor 488 C5 maleimide dye was dissolved to 5 mM in anhydrous DMSO. Ubiquitin cysteine mutant recombinant protein was buffer exchanged into 20 mM Hepes and 150 mM NaCl, pH 7.0, using a 10/300 GL Superdex 75 Increase prepacked column (Cytiva). The fractions containing the protein were combined, concentrated to 100 μM and labeled with a five times molar excess of fluorescent dye for 2 hours at room temperature. The excess dye was removed by gel filtration on a 10/300 GL Superdex 75 Increase prepacked column (Cytiva), eluting in 20 mM Hepes, 150 mM NaCl, and 0.5 mM TCEP, pH 7.5. The dye and subsequently dye-labeled protein were protected from light throughout, flash frozen, and stored at −80°C.

### Brd4^BD2^ (wild type and lysine to arginine mutants) labeling with maleimide Alexa Fluor 647

Alexa Fluor 647 C5 maleimide dye was dissolved to 5 mM in anhydrous DMSO. Brd4^BD2^ recombinant protein was buffer exchanged into 20 mM Hepes and 150 mM NaCl, pH 7.0, using 0.5 ml of 7K MWCO Zeba Spin desalting columns (Thermo Fisher Scientific). The protein was concentrated to 100 μM and labeled with a five times molar excess of fluorescent dye for 2 hours at room temperature. The excess dye was removed using a CentriPure P2 desalting column (Generon), eluting in 20 mM Hepes, 150 mM NaCl, and 0.5 mM TCEP, pH 7.5. The dye and subsequently dye-labeled protein were protected from light throughout, flash frozen, and stored at −80°C.

### Preparative scale ubiquitin loading assay with UBE2R1(C93K) and UBE2R1(C93K, S138C, C191S, C223S)

UBE2R1(C93K) and UBE2R1(C93K,S138C,C191S,C223S) (5 μM) were incubated with 0.5 μM Ub E1 and 50 μM Ub for 18 hours at 37°C in 50 mM tris, pH 9.5, 50 mM NaCl, 5 mM ATP, 10 mM MgCl_2_, and 1 mM 2-mercaptoethanol (BME). The Ub-UBE2R1 conjugates were purified by gel filtration on a Superdex 75 pg 16/600 in 50 mM tris, 150 mM NaCl, and 0.5 mM TCEP, pH 7.5, concentrated, flash frozen with liquid nitrogen, and stored at −80°C. Samples were resolved by SDS-PAGE and unmodified/modified UBE2R1 was visualized by Coomassie staining.

### BMOE cross-linking of UBE2R1(C93K,S138C,C191S,C223S)-Ub with His_6_-TEV-Ub(G76S,K48C)-Brd4^BD2^

UBE2R1(C93K,S138C,C191S,C223S)-Ub (125 nmol) was incubated with 15 mM TCEP for 20 min at ambient temperature and desalted on a HiTrap desalting column (Cytiva) in cross-linking buffer (20 mM Hepes and 150 mM NaCl, pH 7.0). The fractions were pooled and immediately a 15-fold molar excess of bismaleimidoethane (BMOE) was added from a 30 mM DMSO stock. His-TEV-Ub(K48C,G76S)-Brd4^BD2^ (625 nmol) was treated with 15 mM TCEP and desalted in cross-linking buffer. Following a 60-min incubation, BMOE-UBE2R1(C93K,S138C,C191S,C223S)-Ub was directly desalted into the 6×His-TEV-Ub^K48C,G76S^-Brd4^BD2^ sample, and cross-linking was allowed to occur overnight at 4°C. The reaction was quenched with 2-mercaptoethanol (10 mM). The desired cross-linked product, 6×His-TEV-Ub(K48C,G76S)-Brd4^BD2^-BMOE-UBE2R1(C93K,S138C,C191S,C223S)-Ub, was confirmed with SDS-PAGE. The complex was then bound to 1.5 ml of Ni-NTA resin (Cytiva), washed with 20 mM imidazole, and eluted with 500 mM imidazole. The complex was then buffer exchanged into 50 mM tris-HCl and 0.5 mM TCEP, pH 7.5, and further purified by anion exchange chromatography on a 5-ml HiTrap Q HP prepacked column (Cytiva). The complex was eluted on a gradient of buffer A (50 mM tris-HCl and 0.5 mM TCEP, pH 7.5) to buffer B (50 mM tris-HCl, 500 mM NaCl, and 0.5 mM TCEP, pH 7.5). The fractions containing pure cross-linked 6×His-TEV-Ub(K48C,G76S)-Brd4^BD2^-BMOE-UBE2R1(C93K,S138C,C191S,C223S)-Ub were concentrated, flash frozen with liquid nitrogen, and stored at −80°C.

### In vitro ubiquitination assay

The E1 Ube1 (150 nM), the UBE2D2 or UBE2R1 (5 μM), Brd4^BD2^ (5 μM), NEDD8-CRL2^VHL^ (150 nM), and MZ1 (5 μM) were mixed with ubiquitin (100 μM) and incubated in 20 mM Hepes, 150 mM NaCl, 0.5 mM TCEP, and 5 mM MgCl_2_, pH 7.5, for 5 min at room temperature. ATP (3 mM) was added, and the mixture was incubated at room temperature. The reaction was quenched with reducing SDS sample buffer. The protein species were resolved by SDS-PAGE on a 12% bis-tris NuPAGE gel using Mops running buffer, run at 200 V for 40 min. The gel was stained with Coomassie Instant Blue.

### UDPRP production

The cysteine reactive photocrosslinker, *N*-maleimido diazirine, was synthesized as described in (*77*). Stocks of *N*-maleimido diazirine were dissolved to 10 mM using anhydrous DMSO and stored in 50-μl aliquots at −20°C for months. Ubiquitin(E34C) was buffer exchanged into degassed 50 mM tris and 150 mM NaCl, pH 7.0, using a Centri Pure Zetadex-25 gel filtration column (Generon) and labeled with *N*-maleimido diazirine at room temperature for 2 hours using a five times molar excess of *N*-maleimido diazirine. Excess *N*-maleimido diazirine was removed using a Centri Pure Zetadex-25 gel filtration column and 50 mM tris, 150 mM NaCl, and 0.5 mM TCEP, pH 7.5, as running buffer. All buffers were degassed, all dye labeling reactions and dye labeled proteins were protected from light, and all products were analyzed by intact liquid chromatography–mass spectrometry (LC-MS). Photocrosslinker-labeled ubiquitin was conjugated to the active site of UBE2R1(C93K) in a ubiquitin-loading assay (described above). The stable photocrosslinker-labeled Ub(E34C)-UBE2R1(C93K) conjugate was purified by gel filtration on a Superdex 75 pg 16/600 column in 20 mM Hepes, 150 NaCl, and 0.5 mM TCEP, pH 7.5, concentrated, flash frozen with liquid nitrogen, and stored at −80°C.

### Sortase-mediated biotinylation of UBE2R1

His_6_-Sortase was used to conjugate the peptide biotin-LPTGG (synthesized by Peptide2) to the N-terminal glycine residue of UBE2R1. His_6_-Sortase (1 mM), 20 mM UBE2R1, and 200 mM biotin-LPTGG peptide were incubated at 37°C for 15 min in 50 mM tris and 150 mM NaCl, pH 7.0. Imidazole was added to 10 mM and the His_6_-sortase was removed immediately by Ni-NTA chromatography. Excess peptide was removed by Centri Pure Zetadex-25 gel filtration column using 50 mM tris, 150 mM NaCl, and 0.5 mM TCEP, pH 7.5, as running buffer. Biotinylated-UBE2R1 was concentrated to between 5 and 10 mg/ml using a Vivaspin centrifugal concentrator (Sartorius) (10 kDa MWCO, 3000*g*, 4°C), flash frozen in liquid nitrogen, and stored at −80°C.

### UDPRP cross-linking assay

Photocrosslinking reactions (20 μl) were performed with UDPRP (10 μM) and (NEDD8)-CRL2^VHL^ (3 μM) in an 18-well glass-bottom plate (ibidi) in reaction buffer (20 mM Hepes, pH 7.5, 150 mM NaCl, and 1 mM TCEP) for visualization by Western blot. Higher concentrations of both UDPRP (20 μM) and the (NEDD8)-CRL2^VHL^ (5 μM) were used when visualizing the cross-linked product by Coomassie staining. In both cases, samples were divided into two portions. One portion was irradiated at 365 nm, the plate was kept on an ice-cold metal block located 10 cm away from a handheld ultraviolet (UV) lamp (BLE-8T365, Spectroline) for 5 min, and the other portion was preserved in the dark on ice. Samples were resolved by SDS-PAGE and photocrosslinked products were visualized by Coomassie staining or immunoblotting.

### Immunoblotting

Primary antibodies: Rbx1 anti-rabbit, 1:10,000 (Cell Signaling, no. 11922). Secondary antibodies and dyes: IRDye 680LT Donkey anti-Rabbit and IRDye 800CW Streptavidin, both 1:5000 (Licor). Samples were diluted 1:3 in 2× reducing LDS sample buffer (NuPage) and proteins were separated by SDS-PAGE on a 12% polyacrylamide bis-tris gel (NuPage) in 1% MES buffer. Proteins were transferred to nitrocellulose membrane using iBlot 2 Gel Transfer Device (Invitrogen). Membranes were blocked for 1 hour in 5% milk in PBS-T and incubated overnight with primary antibodies, then for 1 hour with secondary antibodies and dyes (1:5000) before imaging on the Licor (Odyssey DLx).

### In vitro stUbl-mediated ubiquitination assay to verify the activity of the photocrosslinker-labeled ubiquitin E34C

The activity of the photocrosslinker-labeled ubiquitin E34C was compared to wild-type ubiquitin in a stUbl assay containing the SUMO-targeted E3 ligase, RNF4, UBE2D1, and UBA1. The assay and protein purification methods are described in detail in (*74*).

### Preparation of samples for cryo-EM

Frozen stocks of recombinant protein and PROTAC were thawed and kept at 4°C throughout the sample preparation process. For the open non–cross-linked Brd4^BD2^-MZ1-(NEDD8)-CRL2^VHL^-UBE2R1-Ub structure, 6 μM NEDD8-CRL2^VHL^ (1 equiv), MZ1 (1.5 equiv), Brd4^BD2^ (1.5 equiv), and UBE2R1(C93K)-Ub conjugate (1.5 equiv) were incubated for 10 min at 4°C. For the closed cross-linked (NEDD8)-CRL2^VHL^-MZ1-Brd4^BD2^-Ub(G76S,K48C)-UBE2R1(C93K, S138C, C191S, C223S)-Ub structure, 6 μM NEDD8-CRL2^VHL^ (1 equiv), MZ1 (1.5 equiv), and Brd4^BD2^-Ub(G76S,K48C)-UBE2R1(C93K, S138C, C191S, C223S)-Ub (1.5 equiv) were incubated for 10 min at 4°C. The complexes were desalted on a 0.5-ml 7K MWCO Zeba Spin Desalting Column in 20 mM Hepes, 150 mM NaCl, and 0.5 mM TCEP, pH 7.5. Quantifoil R1.2/1.3 holey carbon copper 400 mesh grids were glow discharged for 60 s at 35 mA using a Quorum SC7620. Protein (3.5 μl) at 4 μM was applied to the cryo-EM grids and was vitrified in liquid ethane on a Vitrobot Mark IV (Thermo Fisher Scientific) at 4°C and 100% humidity (wait time = 10 s, blot force = 4, blot time = 3.5 s, blot total = 1, drain time = 0 s).

### Cryo-EM data acquisition

For the open non–cross-linked Brd4^BD2^-MZ1-(NEDD8)-CRL2^VHL^-UBE2R1-Ub structure, cryo-EM data were collected on Glacios transmission electron microscope (Thermo Fisher Scientific) operating at 200 keV. Micrographs were acquired using a Falcon4i direct electron detector (Thermo Fisher Scientific), operated in electron counting mode. A total electron exposure of 26 e^−^/Å^2^ was applied. EPU (Thermo Fisher Scientific, version 3.0) was used to collect micrographs at 190,000x nominal magnification (0.74 Å/pixel at the specimen level) with a nominal defocus range of −1.7 to −3.2 μm. Stage shifts with aberration-free image shift (AFIS) mode was used to center multiple foil holes and image shift was used to acquire high-magnification images in the center of each targeted hole. A total of 4961 movies were collected in Electron Event Representation (EER) format.

For the closed cross-linked (NEDD8)-CRL2^VHL^-MZ1-Brd4^BD2^-Ub(G76S,K48C)-UBE2R1(C93K, S138C, C191S, C223S)-Ub structure, cryo-EM data were collected on a Krios transmission electron microscope (Thermo Fisher Scientific) operating at 300 keV. Micrographs were acquired using a K3 direct electron detector (Gatan), operated in electron counting mode. A total electron exposure of 38 e^−^/Å^2^ was applied. EPU (Thermo Fisher Scientific, version 3.0) was used to collect micrographs at 105,000x nominal magnification (0.825 Å/pixel at the specimen level) with a nominal defocus range of −1.2 to −3.0 μm. Stage shifts with AFIS mode was used to center multiple foil holes and image shift was used to acquire eight high-magnification images following a template around the edge of each targeted hole. A total of 14,047 movies were collected in Tag Image File Format (TIFF) format.

### Cryo-EM image analysis and model building

Image processing pipelines are described in figs. S3 and S10. Cryo-EM movies were imported into CryoSPARC v.4.4.0-v4.4.1 (*78*) for patch motion correction, patch CTF estimation, and manual curation. For the open non–cross-linked Brd4^BD2^-MZ1-(NEDD8)-CRL2^VHL^-UBE2R1-Ub, manual picking was performed on 84 micrographs that were used for template picking and 2D classification. Good templates were used for Topaz training and particle picking (*79*). A total of 405,567 particles were extracted with a 512 pixel box size (2× binning). Classification was achieved using ab initio reconstruction into four classes. Particles for the best class were submitted to refinement, re-extracted with the full 512 pixel box size, and submitted to nonuniform refinement in CryoSPARC (*80*). For the closed cross-linked (NEDD8)-CRL2^VHL^-MZ1-Brd4^BD2^-Ub(G76S,K48C)-UBE2R1(C93K, S138C, C191S, C223S)-Ub structure, particle picking was performed on 4458 movies with crYOLO using a general model for low-pass–filtered images (*81*). Particles were submitted to 2D classification and good templates were used for Topaz training and particle picking (*79*). A total of 748,020 particles were extracted with a 432 pixel box size (3× binning). Classification was achieved by 2D classification followed by ab initio reconstruction and heterogeneous refinement into three classes. The best class was submitted to 3D classification into three classes. The particles from the best classes were combined, re-extracted to 480 pixels, and submitted to nonuniform refinement CryoSPARC (*80*). Representative 2D classes, orientation diagnostics, local resolution estimations, and gold standard Fourier shell correlation curves were generated with CryoSPARC (*80*) and are shown in figs. S3 and S10. Model building was achieved using atomic models from AlphaFold and Protein Data Bank (PDB) entries 5T35, 5N4W, 4AP4, and 6TTU, which were docked into the cryo-EM maps using rigid-body fitting with UCSF ChimeraX (*82*). The model was refined using ISOLDE (*83*) until reasonable agreement between the model and data was achieved.

### Nuclear magnetic resonance

Solution NMR data were acquired at 298 K on a Bruker Avance III 600 MHz spectrometer and cryogenic TCI probe. Each protein sample was prepared at a concentration of 150 μM and recorded in buffer 20 mM sodium phosphate, 150 mM NaCl, 0.5 mM TCEP, and 5% D_2_O, pH 7.0. ^15^N-^1^H-HSQC spectra were recorded with 128 points in the ^15^N dimension and processed with 256 points using Bruker TopSpin version 3.6. Previous assignment for Ub (*75*) matched well for Ub(^15^N) and signals from Ub in UBE2R1(C93K)~Ub(^15^N) were able to be assigned. Spectra were analyzed and printed using CARA. The chemical shift perturbation (CSP) was calculated according to CSP = {(δ_HA_–δ_HB_)^2^ + [(δ_NA_–δ_NB_)/5]^2^}^1/2^.

### Mass photometry

Interferometric scattering microscopy (*84*) was carried out using the commercially available oneMP (Refeyn). The cryo-EM buffer, 20 mM Hepes, 150 mM NaCl, and 0.5 mM TCEP, pH 7.5, was filtered through a 0.22-μm syringe filter, and gasket wells (Grace Bio-labs CW-50R-1.0) along with high-precision 24 × 50 mm coverslips (Marienfeld) were prepared at measurement according to Wu and Piszczek (*85*). Buffer (10 μl) was used for focusing in regular mode (128 × 34 binned pixels 18.0 μm^2^ detection area) and all data were recorded for 60 s using AquireMP software (Refeyn). Calibration standards for mass calibration consisting of conalbumin (*M*_r_ = 75 000), aldolase (*M*_r_ = 158 000), ferritin (*M*_r_ = 440 000), and thyroglobulin (*M*_r_ = 669 000) were prepared from the gel filtration HMW calibration kit (Cytiva). The various complexes containing CRL2^VHL^ were measured in the same buffer in a concentration range of 20 to 30 nM. All data were processed and analyzed in DiscoverMP (Refeyn).

### Intact LC-MS

For all intact LC-MS measurements, 0.5 μg of protein was diluted in the appropriate buffer and injected in a 20-μl volume. LC-MS was carried out with an Agilent 1200 LC-MS system fitted with a Max-Light Cartridge flow cell coupled to a 6130 Quadrupole spectrometer. An Agilent ZORBAX 300SB-C3 5 μm, 2.1 × 150 mm column was used unless otherwise stated. Protein UV absorbance was monitored at 214 and 280 nm. MS acquisition was carried out in positive ion mode and total protein masses were calculated by deconvolution within the MS Chemstation software (Agilent Technologies).

### MS sample preparation and data acquisition of in vitro samples

#### Bromodomain lysine ubiquitination sites

Proteins from the regions of the gel relating to ubiquitin-modified Brd4^BD2^ forms were in-gel trypsin digested (*86*) and alkylated with chloroacetamide (CAA), and final peptides were resuspended in 0.1% trifluoroacetic acid (TFA) and 0.5% acetic acid before analysis by LC-MS/MS. Samples were analyzed on a Thermo Fisher Scientific Lumos Tribrid mass spectrometer coupled with a Thermo Dionex Ultimate 3000 RSLC HPLC. The buffers used for HPLC were 0.1% formic acid as buffer A and 80% acetonitrile (ACN) with 0.08% formic acid as buffer B. Trap column Acclaim pepmap 100 (C5, 100 μM × 2 cm) was used before the main column for sample concentration and cleanup. The peptide samples were loaded onto the trap column using a loading pump with 3% ACN and 0.1% TFA at a flow rate of 5 μl/min. The main column used was EASY-Spray column (C18, 2 μm, 75 μm × 50 cm) with a nano electrospray emitter built in. The flow rate of 300 nl/min was maintained throughout the run. Peptides were separated with a 55-min segmented gradient starting from 3 to 35% buffer B over 45 min, 35 to 95% buffer B over 47 min, and held for 5 min. The separated peptides were then analyzed on a Lumos Mass spectrometer. Spray voltage was set to 2 kV, RF lens level was set at 30%, and ion transfer tube temperature was set to 275°C. The mass spectrometer was operated in data-dependent settings with 3 s cycle time. The full scan was performed in the range of 375 to 1500 mass/charge ratio (*m*/*z*) at a nominal resolution of 120,000 at 200 *m*/*z* and automatic gain control (AGC) was set to 400,000 with a custom maximum injection time of 50 ms. This was followed by the selection of the most intense ions above an intensity threshold of 5000 for higher-energy collision dissociation (HCD) fragmentation, with normalized collision energy set to 30. MS2 scans were acquired for charge states 2 to 7 using an isolation width of 1.6 *m*/*z* and a 30-s dynamic exclusion duration. MS2 scans were done using an AGC target set to 50,000 and a maximum fill time of 50 ms.

#### Ubiquitin loading with UBE2R1(C93K) and UDPRP cross-linking assays

Proteins from the regions of the gel relating to modified and unmodified Ube2R1 or UDPRP photocrosslinked products were in-gel trypsin digested (*86*) and alkylated with CAA, and final peptides were resuspended in 0.1% TFA and 0.5% acetic acid before analysis by LC-MS/MS. This was performed using a Q Exactive mass spectrometer (Thermo Fisher Scientific) coupled to an EASY-nLC 1000 liquid chromatography system (Thermo Fisher Scientific), using an EASY-Spray ion source (Thermo Fisher Scientific) running a 75 μm × 500 mm EASY-Spray column at 45°C. Two MS runs (of 60 and 150 min) were prepared using approximately 15% total peptide sample each. To boost sensitivity, a top three data-dependent method was applied using a full scan (*m*/*z* 300 to 1800) with resolution *R* = 70,000 at *m*/*z* 200 (after accumulation to a target value of 1,000,000 ions with a maximum injection time of 20 ms). For the 60-min gradient, the three most intense ions were fragmented by HCD and measured with a resolution of *R* = 70,000 (60 min run) or 35,000 (150 min run) at *m*/*z* 200 (target value of 1,000,000 ions and maximum injection time of 500 ms) and an intensity threshold of 2.1 × 10^4^. Peptide match was set to “preferred.” Ions were ignored if they had unassigned charge state 1, 8, or >8 and a 10-s (60 min run) or 25-s (150 min run) dynamic exclusion list was applied.

### MS data analysis of in vitro samples

#### Bromodomain lysine ubiquitination sites

Data analysis used MaxQuant version 2.4.0.0 (*87*). Default settings were used with a few exceptions: A database of all the recombinant proteins included in the in vitro ubiquitination assay was used. Digestion was set to trypsin with a maximum of three missed cleavages. Match between runs was not enabled. Oxidation (M), Acetyl (Protein N-term), and GlyGly (K) were included as variable modifications, with a maximum of three per peptide allowed. Carbamidomethyl (C) was included as a fixed modification. Only peptides of maximum mass 8000 Da were considered. Protein and peptide level FDR was set to 1% but no FDR filtering was applied to the identified sites. Manual MS/MS sequence validation was used to verify GlyGly (K) peptide identifications and only peptides with an Andromeda score >100, a localization probability >0.75, and a mass error <1 part per million (ppm) were considered.

#### Verification of the ubiquitin loading assay with UBE2R1(C93K)

Data analysis used MaxQuant version 1.6.1.0 (*87*). Default settings were used with a few exceptions: A database of all the recombinant proteins included in the ubiquitin-loading assay was used. Digestion was set to Trypsin/P (ignoring lysines and arginines N-terminal to prolines) with a maximum of three missed cleavages. Match between runs was not enabled. Oxidation (M), Acetyl (Protein N-term), and GlyGly (K) were included as variable modifications, with a maximum of four per peptide allowed. Carbamidomethyl (C) was included as a fixed modification. Only peptides of maximum mass 8000 Da were considered. Protein and peptide level FDR was set to 1% but no FDR filtering was applied to the identified sites. Manual MS/MS sequence validation was used to verify GlyGly (K) peptide identifications and only peptides with an Andromeda score >100, a localization probability >0.75, and a mass error <1 ppm were considered.

#### Identification of UDPRP cross-links

Data analysis was performed with MaxQuant version 2.4.0.0 (*87*). Default settings were used with a few exceptions. A database of all the recombinant proteins included in the UDPRP cross-linking assay was used. Digestion was set to Trypsin/P (ignoring lysines and arginines N-terminal to prolines) with a maximum of eight missed cleavages. Match between runs was enabled. Before running the search, an additional cross-linker was added to MaxQuant configurations describing *N*-maleimido-diazirine and was named “NMD.” Linked composition H(9)O(2)C(8)N, mass 151.0633285383 Da. Hydrolyzed composition H(9)O(2)C(8)N(3), mass 179.0694765487. Specificity 1 was C, position in peptide 1 was set to anywhere. Protein N-term 1 and C-term 1 were selected. Specificity 2 set for any amino acid (ACDEFGHIKLMNPQRSTVWY), position in peptide 2 was anywhere, and protein N-term 2 and protein C-term 2 were selected. To search for cross-linked peptides, the cross-linker NMD (noncleavable) was selected, minimum length for a paired sequence was set to 3, and the maximum peptide mass was 12,000 Da. The minimum peptide length for unspecified peptide search was 8 and the maximum was 25. The search included both intraprotein and interprotein cross-linked peptides. Oxidation (M), Acetyl (Protein N-term), and Carbamidomethyl (C) were included as variable modifications, with a maximum of five per peptide allowed. First search was performed with Oxidation (M) and Acetyl (Protein N-term). Protein and cross-linked peptide level FDR was set to 1% but no FDR filtering was applied to the identified sites. Manual MS/MS sequence validation was used to verify interprotein NMD cross-linked peptide identifications and only peptides with an Andromeda score >95, a localization probability >0.75, and a mass error <1 ppm were considered.

### Multiple sequence alignments

Coding sequences for human BRD2 (P25440), BRD3 (Q15059), BRD4 (O60885), and BRDT (Q58F21) were taken from UniProt. Individual FASTA files were generated using the boundaries of the BD1 and BD2. Multiple sequence alignments were carried out in Jalview (*88*) using MUSCLE (*89*). Alignments for figures were exported directly from Jalview.

### Synthesis of 1-(2-(3-methyl-3H-diazirin-3-yl)ethyl)-1H-pyrrole-2,5-dione

All chemicals, unless otherwise stated, were commercially available and used without further purification. Commercially available dry solvents were used. Flash column chromatography was performed using a Teledyne Isco Combiflash Rf with prepacked Redisep RF Normal phase disposable columns. NMR spectra were recorded on a Bruker 400 MHz or 500 MHz as specified. ^13^C spectra were ^1^H decoupled. Chemical shifts (δ) are reported in ppm and referenced to the residual solvent signals: ^1^H NMR δ (ppm) = 7.26 (CDCl_3_), ^13^C NMR δ (ppm) = 77 (CDCl_3_). Signal splitting patterns are described as singlet (s) and triplet (t). Coupling constants (*J*) are measured in hertz (Hz).

Diisopropyl azodicarboxylate (360 μl, 1.8 mmol) was added dropwise to a solution of 2-(3-methyldiazirin-3-yl)ethanol (150 mg, 1.5 mmol), triphenylphosphine (432 mg, 1.65 mmol) and maleimide (160 mg, 1.65 mmol) in THF (3 ml) at 0°C and the reaction mixture was stirred overnight at room temperature. After solvent evaporation, the residue was purified by column chromatography, with an elution gradient of 0 to 50% of EtOAc in heptane, to afford 1-(2-(3-methyl-3H-diazirin-3-yl)ethyl)-1H-pyrrole-2,5-dione as a colorless oil (139 mg, 52%).^1^H NMR (500 MHz, CDCl_3_): 6.74 (2H, s), 3.59 (2H, t, *J* = 7.1 Hz), 1.62 (2H, t, *J* = 7.1 Hz), 1.08 (3H, s). ^13^C NMR (100 MHz, CDCl_3_): 170.4, 134.3, 33.4, 33.2, 23.9, 19.1. NMR spectra (fig. S14) were in agreement with the published data (*77*).

### Cellular degradation assay

#### Construct design

The wild-type, 1-point, 3-point, and 8-point lysine-to-arginine mutant NanoLuc-Brd4^BD2^ constructs were originally designed in Snapgene (V 6.2.1) and were subsequently synthesized and integrated into a pCDNA3.1(+) (containing a CMV promoter) backbone using Invitrogen’s GeneArt services.

#### Cell culture

HEK293 cells were obtained from the American Type Culture Collection (ATCC). HEK293 was cultured in DMEM supplemented with 10% (v/v) fetal bovine serum (FBS), 1% L-glutamate, and 1% (v/v) penicillin/streptomycin (pen/strep) at 37°C, 5% CO_2_, and 95% humidity.

#### Transient transfection of the different lysine mutant Brd4^BD2^ constructs into HEK293

Twenty hours before initiation of the MZ1 titration, HEK293 cells were transiently transfected with one of the four Brd4^BD2^ mutant constructs described previously. To do this, 10 ng of the intended construct along with 1 μg of carrier DNA was added to 100 μl of Optimem media along with 4 μl of FUGENE HD transfection reagent in an Eppendorf. The transfection mixture was then left for 20 min, before its addition to 2 × 10^6^ HEK293 cells in 10 ml of fresh DMEM supplemented with 10% (v/v) fetal bovine serum (FBS), 1% L-glutamate, and 1% (v/v) pen/strep. After thorough mixing, 100 μl of the DMEM, cells, and transfection mixture was then added to each well of a 96-well white-walled plate and left overnight to adhere at 37°C, 5% CO_2_, and 95% humidity.

#### MZ1 dose-response degradation assay

MZ1 dose-response degradation assay was performed using the previously described transiently transfected HEK293 cells. MZ1 was dissolved into a 10 mM concentrated stock solution in DMSO from which MZ1 was further diluted to a working concentration range of 10 μM to 6 pM in Optimem and was subsequently added to cells at the start of experimentation. Additional controls including a vehicle control were set at a comparable concentration to the DMSO present in the highest MZ1 treatment. Cells were left to incubate with compound for 4 hours at 37°C, 5% CO_2_, and 95% humidity. Nano-Glo Vivazine live cell substrate was then added to all wells of the 96-well plate before luminescent measurements were taken using a PHERAstar *FSX* microplate reader. Luminescent plate reads from these data were then plotted and analyzed using Prism (v. 10.2.2, GraphPad).

### MS sample preparation and data acquisition of cellular samples

HEK293 cells were cultured in DMEM with 10% FBS, 2 mM l-glutamine, and 1% streptomycin/penicillin. Cells were maintained at 37°C under 5% CO_2_ in a humidified tissue culture incubator. Cells at 70% confluence were treated with 1 μM MZ1 dissolved in DMSO, while control cells were treated with an equivalent volume of DMSO for 15 min. Cells were harvested by washing twice with phosphate-buffered saline (PBS) and scraping on ice in lysis buffer (50 mM TEABC, pH 7.5, and 8 M urea) supplemented with 1× Complete protease inhibitor and phosphatase inhibitor cocktail tablet. Cells were lysed by sonication using 40% amplitude with a 10-s on/off cycle for 3 min on ice. Lysates were clarified by centrifugation for 20 min at 13,000*g* at 4°C. Protein concentration was estimated using the Pierce bicinchoninic acid method. Five milligrams of protein was used for in-solution digestion from each sample. Proteins were reduced with 5 mM DTT at room temperature for 30 min and alkylated with 10 mM CAA in the dark at room temperature for 15 min. Samples were then digested with Lys-C (1:1000) at room temperature for 4 hours. The samples were then diluted with 50 mM triethylammonium bicarbonate buffer (TEAB) to a urea concentration of 1.5 M and further digested with trypsin (1:20) at room temperature for 16 hours. The digestion was quenched by adding TFA to a final concentration of 1% (v/v) TFA, and the samples were desalted using 200 mg of Sep-Pak C_18_ cartridges (Waters). For Sep-Pak cleanup, the following solvents were prepared fresh: activation solvent [100% (v/v) ACN], solvent 1 [0.1% (v/v) TFA], solvent 2 [0.1% (v/v) formic acid (FA)], and solvent 3 [50% (v/v) ACN in 0.1% (v/v) FA]. Sep-Pak cartridges were equilibrated with 5 ml of 100% ACN, followed by 5 ml of 50% ACN and 0.1% FA, and finally 5 ml of 0.1% TFA twice. Samples were then loaded onto the equilibrated C_18_ cartridges, washed four times with 5 ml of 0.1% TFA, followed by a wash with 5 ml of 0.1% FA. Samples were then eluted with 3 ml of 50% ACN and 0.1% FA. Desalted samples were dried to completeness in a SpeedVac concentrator. These digested peptides were dissolved in 1.5 ml of 1× HS IAP bind buffer. The dissolved peptides were centrifuged for 5 min at 10,000*g* at 4°C. The antibody-bead slurry (PTMScan HS K-ε-GG Remnant Magnetic Immunoaffinity Beads no. 34608) was pipetted to the desired volume (5 μl for each enrichment). These beads were washed three times with 1 ml of ice-cold 1× PBS. The soluble peptides were transferred into the antibody-bead slurry tube. Tighten the cap and seal the top of the tube to avoid leakage. This antibody-peptide mixture was incubated on an end-over-end rotator for 2 hours at 4°C. To bring down the beads, the tubes were spun at 2000*g* for 2 to 5 s. These microfuge tubes were kept in the magnetic separation rack for 10 s. The unbound peptide solution was discarded. Chilled HS IAP wash buffer (1×; 1 ml) was added to the beads and mixed by inverting the tube five times. Tubes were centrifuged and placed in the magnetic stand for 10 s and all wash buffers were removed. This was repeated three more times for a total of four washes with HS IAP wash buffer. The beads were also washed two times with 1× ice-cold PBS. The diGly modified peptides were eluted from the beads by adding 50 μl of IAP Elution Buffer (0.15% TFA) to the beads. These were incubated on a Thermomixer set at 200 rpm at room temperature for 10 min. The tubes were centrifuged and placed in the magnetic rack. The eluted peptides were transferred to a different microcentrifuge tube. Another 50 μl of IAP Elution Buffer (0.15% TFA) was added to the beads to repeat the elution step. Both eluents were combined in the same microcentrifuge tube. These eluted peptides were cleaned using desalting on C_18_. First, C_18_ tips were washed with 50 μl of ACN. This was followed by equilibrating the tips twice with 50 μl of 0.1% formic acid. The eluted DiGly peptides were loaded onto the column, followed by washing twice with 50 μl of 0.1% formic acid. The peptides were eluted twice using 15 μl of 50% ACN in 0.1% formic acid. The eluted peptides were dried using a SpeedVac and analyzed on a mass spectrometer. The dried peptides were reconstituted in 0.1% formic acid and one-third of the sample was analyzed on an Orbitrap Astral mass spectrometer coupled to a Thermo Fisher Scientific Vanquish Neo UHPLC. The peptides were enriched on a trap column and resolved on an analytical column (Easy-Spray PepMap Neo 2 μm C18 75 μm × 150 mm) with 800 nl/min. The gradient for separation was used as 22.5% B at 14 min and 35% at 21 min followed by 9%. Total run time used was 22.6 min. The data were acquired in data-independent acquisition mode in an astral analyzer. The MS data acquired in Orbitrap were operated with a fixed cycle time of 5 ms and with a full scan range of 380 to 980 *m*/*z* at a resolution of 240,000. The AGC was set to 500% and ion injection time is custom. Precursor ion selection width was kept at 4-Th and peptide fragmentation was achieved by HCD (normalized collision energy 25%). For DIA mode, scan range used 150 to 2000, AGC target was 500%, ion injection was custom, and detector was Astral.

### MS data analysis of cellular samples

Acquired raw files were processed using DIA-NN, version 1.8 (https://github.com/vdemichev/DIaNN). Human UniProt was used as the protein sequence database. Two missed cleavage and a maximum of two variable modifications per peptide were allowed (acetylation of protein N-termini and oxidation of methionine). Carbamidomethylation of cysteines was set as fixed modification and K-GG was added. This data analysis was carried out using library-free analysis mode in DIA-NN with “deep learning-based spectra and RTs prediction” enabled. MBR was enabled. The search results were further processed in Perseus (*90*). The data were loaded in Perseus in txt format. The replicates were grouped based on their annotation to DMSO and MZ1. Furthermore, the data were transformed in log2 scale, followed by imputation of missing values from a normal distribution with “Processing → Imputation → Replace missing values from normal distribution.” Then, Perseus will shrink the distributions to a factor of “0.3” (width), shift it down by “1.8” (down shift) SDs, and simulate some random values that make up values to fill up the missing values. In addition, we used the whole matrix (mode). These analyzed data were used to generate volcano plots using GraphPad Prism software (Version 8).
