# Supplementary material for: Mechanism of degrader-targeted protein ubiquitinability
Source: Sci Adv. 2024 Oct 11;10(41):eado6492. doi: 10.1126/sciadv.ado6492 (PMC11468923; doi:10.1126/sciadv.ado6492)
Supplement: Supplementary file 1 — Figs. S1 to S22 Legends for movies S1 and S2 Table S1 [file sciadv.ado6492_sm.pdf]

Science Advances
AAAS

Supplementary Materials for

Mechanism of degrader-targeted protein ubiquitinability

Charlotte Crowe *et al.*

Corresponding author: Alessio Ciulli, a.ciulli@dundee.ac.uk

Sci. Adv. **10**, eado6492 (2024)
DOI: 10.1126/sciadv.ado6492

The PDF file includes:

Figs. S1 to S22
Legends for movies S1 and S2
Table S1

Other Supplementary Material for this manuscript includes the following:

Movies S1 and S2

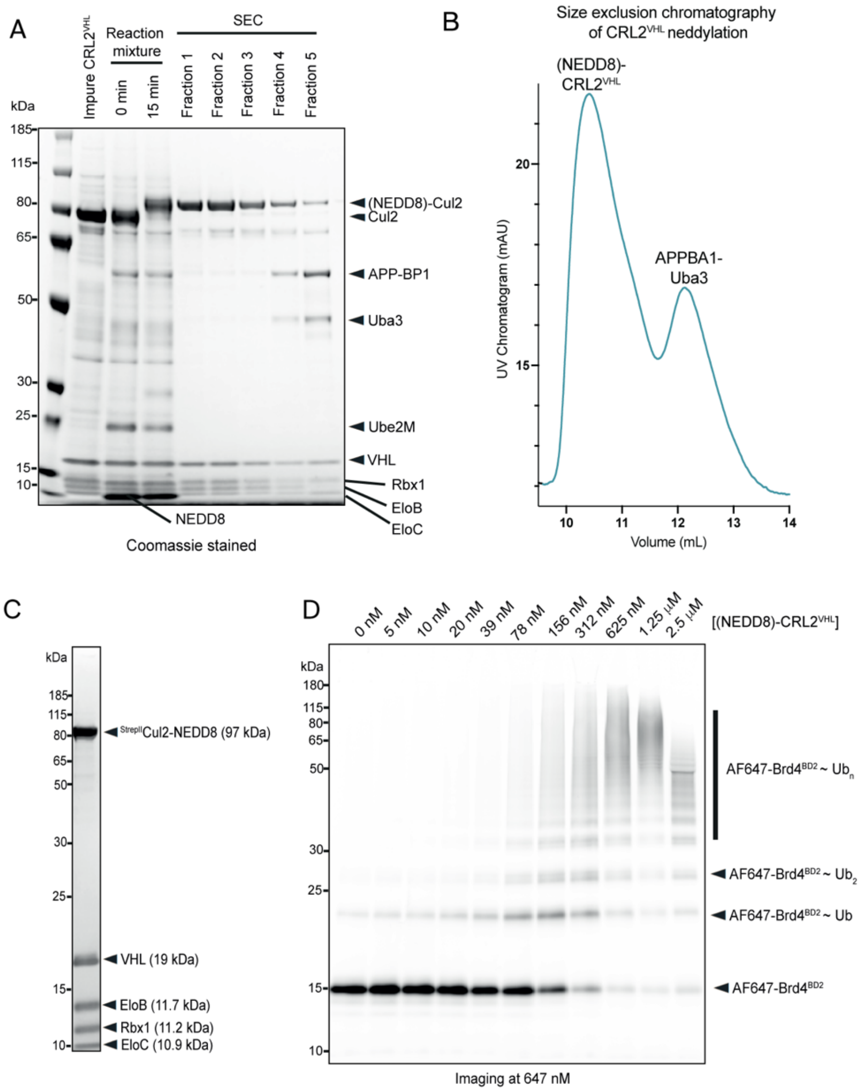

Fig. S1. (NEDD8)-CRL2^{VHL} purification and *in vitro* ubiquitination assay. (**A**) SDS-PAGE of CRL2^{VHL} neddylation reaction and (NEDD8)-CRL2^{VHL} purification fractions by size exclusion chromatography. (**B**) Size exclusion chromatography purification UV trace showing the elution of (NEDD8)-CRL2^{VHL} and APPBP1-Uba3 (cropped to elution volumes of 9-14 mL). (**C**) SDS-PAGE of (NEDD8)-CRL2^{VHL} used for cryo-EM analyses and assays. (**D**) SDS-PAGE (imaged at 647 nm) of Alexa Fluor 647-labelled Brd4^{BD2} *in vitro* ubiquitination, demonstrating the enzymatic activity of recombinant (NEDD8)-CRL2^{VHL}. The reactant species were mixed to final concentrations of Ube1 (150 nM), UBE2D2 (5 µM), Brd4^{BD2} (4 µM), Alexa Fluor 647 maleimide-labelled Brd4^{BD2} (1 µM), NEDD8-CRL2^{VHL} (0-2.5 mM), MZ1 (5 µM) and ubiquitin (100 µM) and incubated in 20 mM HEPES, 150 mM NaCl, 0.5 mM TCEP, 5 mM $MgCl_2$, pH = 7.5 for 5 minutes at room temperature. The reaction was launched with ATP (3 mM) and the samples were quenched after 30 minutes with SDS sample buffer. The protein species were resolved by SDS-PAGE on a 12% Bis-Tris NuPAGE gel using MOPS running buffer, run at 200 V for 40 minutes. The gel was fluorescently imaged at 647 nm to identify Alexa Fluor 647 maleimide-labelled Brd4^{BD2}.

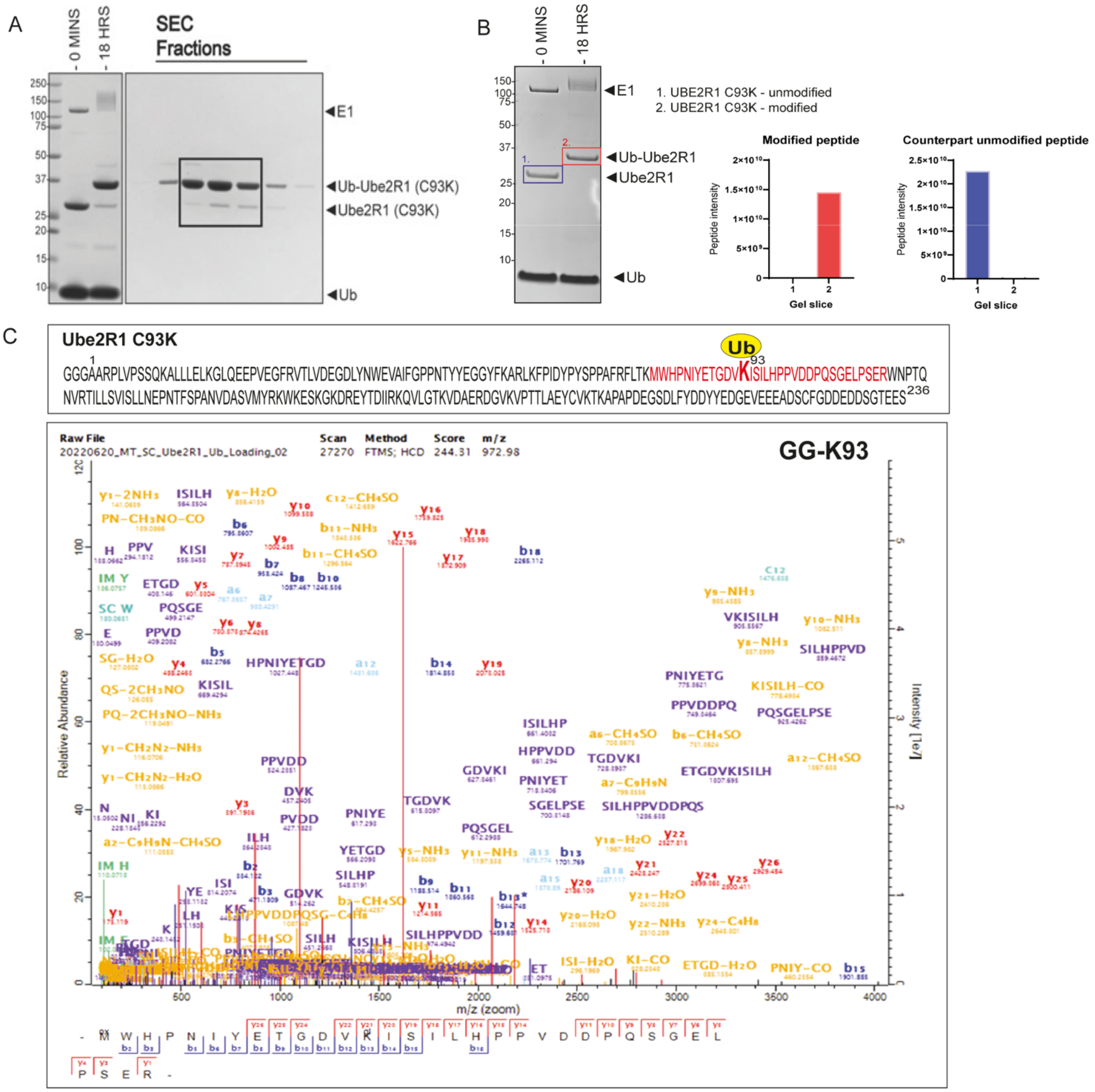

Fig. S2. Preparation and assembly of UBE2R1(C93K)-Ub for cryo-EM. (**A**) Coomassie stained SDS-PAGE of preparative scale ubiquitin loading on UBE2R1(C93K), and size exclusion chromatography fractions. The fractions containing Ub-UBE2R1(C93K), indicated by the black box, were pooled. (**B**) Left: Coomassie stained SDS-PAGE of ubiquitin loading on UBE2R1(C93K) for mass spectrometry analysis (the boxes indicate the bands which were excised for mass spectrometry analysis). Right: Peptide intensity for the GlyGly(K) modified and counterpart unmodified active site peptide (depicted in **C**) from the unmodified band (blue, gel slice 1) and the unmodified band (red, gel slice 2). The active site (C93K) residue is almost fully occupied with ubiquitin. (**C**) Mass spectrometry analysis of ubiquitin loading onto the active site of UBE2R1(C93K). Schematic of the amino acid sequence of UBE2R1(C93K) with ubiquitin modification (yellow, Ub) on the active site C93K, the GlyGly(K) modified peptide sequence is coloured in red and the spectrum for this peptide is shown below. Andromeda score: 244.31.

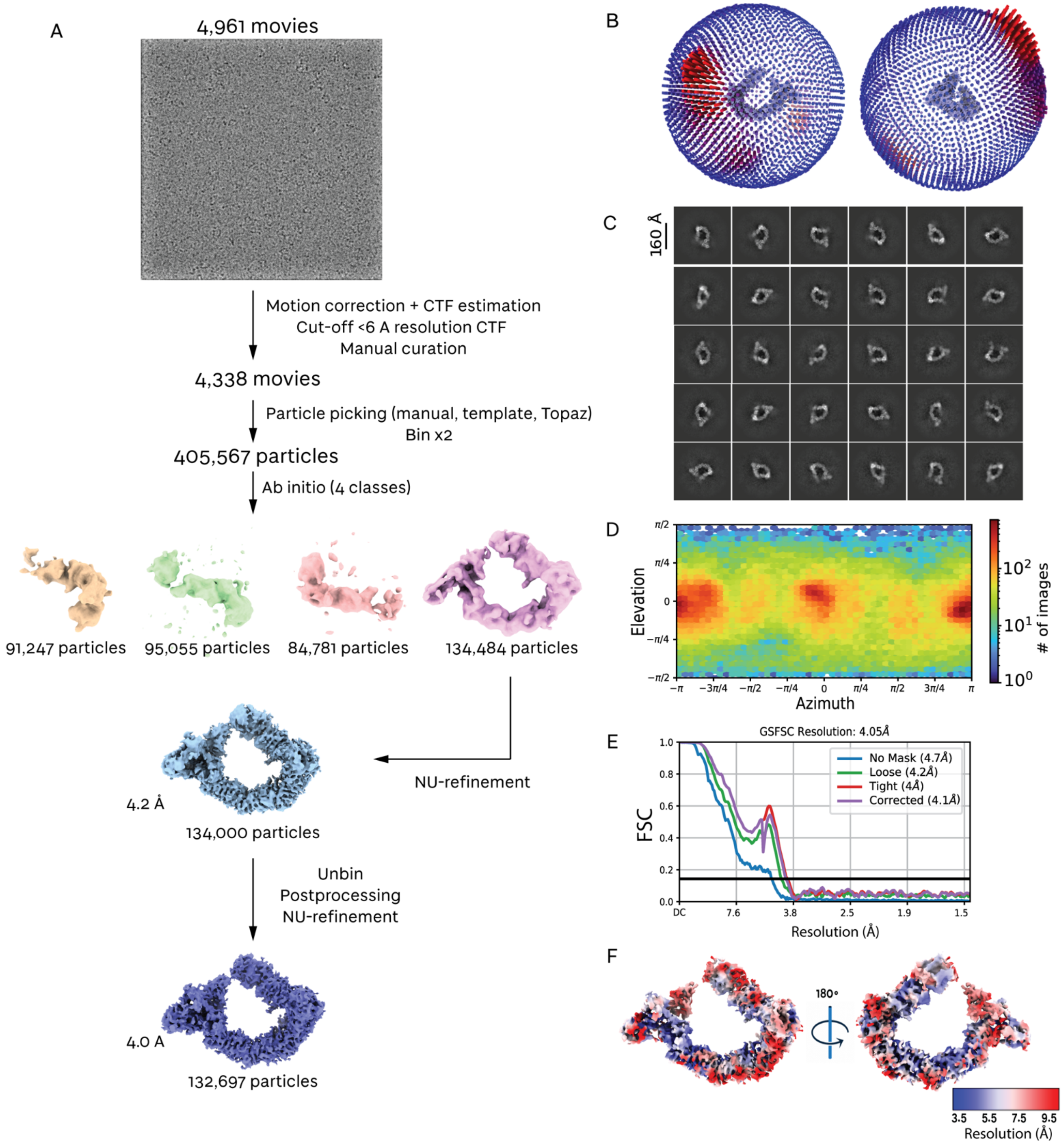

Fig. S3. Cryo-EM image analysis for Brd4^{BD2}-MZ1-(NEDD8)-CRL2^{VHL}-UBE2R1-Ub. (**A**) A schematic for the image processing workflow for the 'open' non-crosslinked Brd4^{BD2}-MZ1-(NEDD8)-CRL2^{VHL}-UBE2R1-Ub complex used to generate cryo-EM maps. (**B**) 3D viewing direction distribution. (**C**) Selected 2D classes. (**D**) 2D viewing direction distribution. (**E**) Gold-standard Fourier shell correlation plot at 0.143. (**F**) Local resolution estimation.

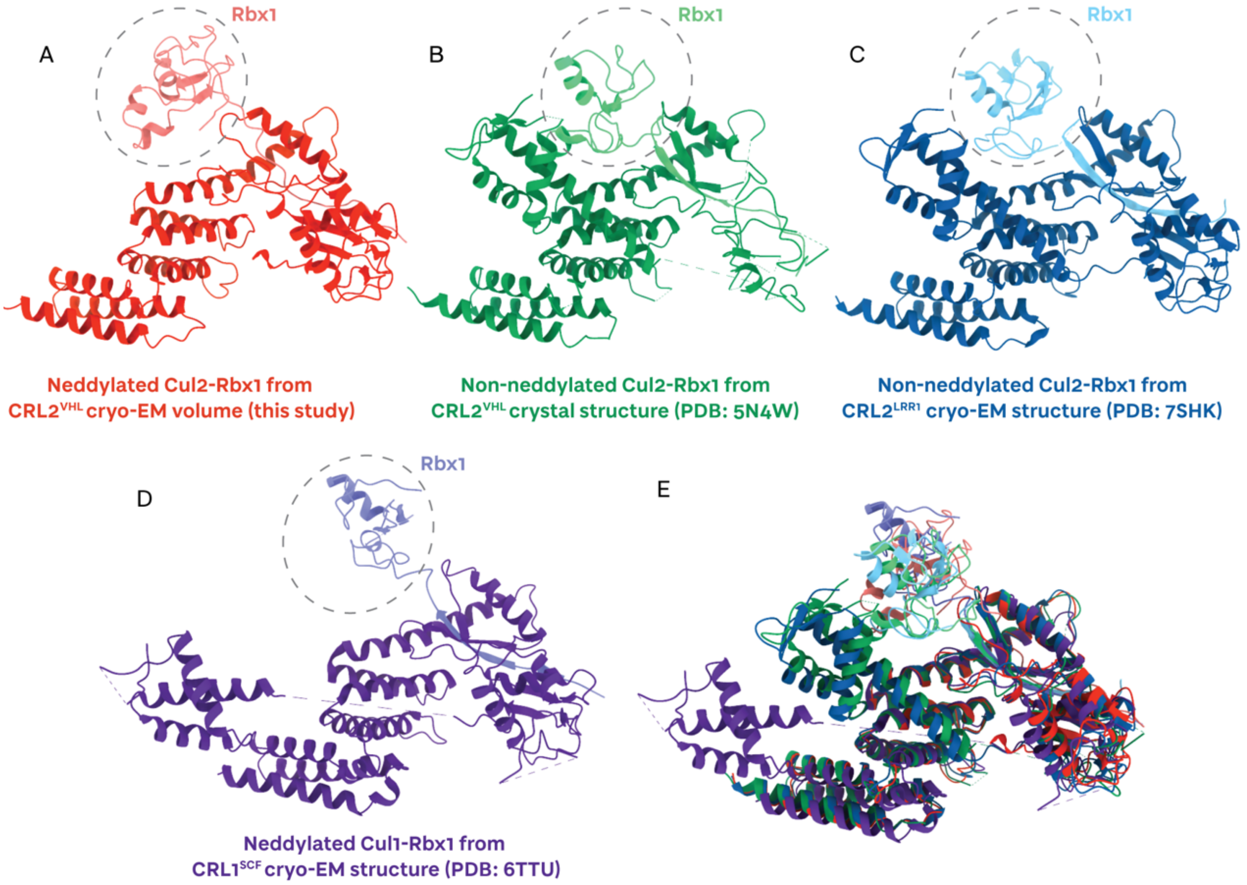

Fig. S4. Structural alignments of CRL2 C-terminal domains (CTDs). (**A**) CTD of Cullin 2 (red) and Rbx1 (pink) in the atomic model built from the 'open' cryo-EM structure in this study. The WHB domain of Cullin 2 is not modelled. The complex is neddylated, and Rbx1 appears to be in a mobile state. (**B**) CTD of Cullin 2 (dark green) and Rbx1 (light green) in the atomic model of the crystal structure of full-length Cul2-Rbx1 in complex with VHL-EloB-EloC (PDB: 5N4W). The complex is unneddylated and the WHB domain of Cullin 2 and Rbx1 are closely packed. (**C**) CTD of Cullin 2 (dark blue) and Rbx1 (light blue) in the atomic model of the cryo-EM structure of full-length Cul2-Rbx1 in complex with LRR1 (PDB: 7SHK, EMDB-25127). (**D**) CTD of Cullin 1 (dark purple) and Rbx1 (light purple) in the atomic model of the cryo-EM structure of full-length Cul1-Rbx1 in complex with SCF (PDB: 6TTU, EMDB-10585). (**E**) Overlay all A, B, C and D atomic models. Rbx1 adopts a range of different orientations.

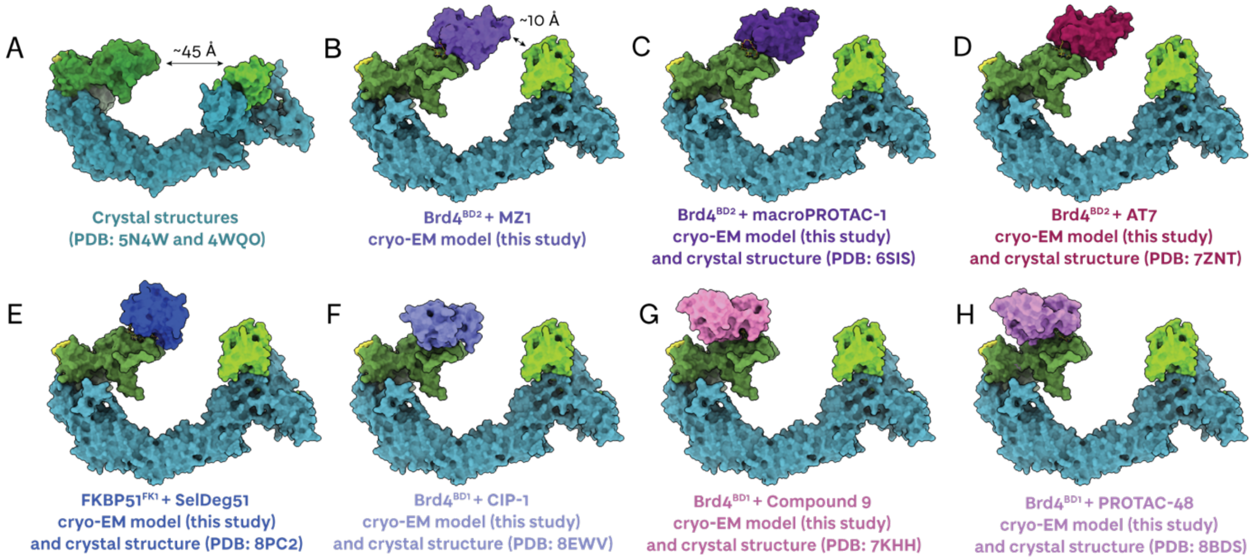

Fig. S5. Comparison of degrader ternary complex crystal structures aligned with the cryo-EM volume from this study. (**A**) Surface representation of the aligned crystal structures of VHL-EloB-EloC-Cul2(NTD) (PDB: 4WQO) with VHL-EloB-EloC-Cul2-Rbx1 (PDB: 5N4W). (**B**) Surface representations of the atomic model of the Brd4^{BD2}-MZ1-(NEDD8)-CRL2^{VHL} complex generated from this study. (**C**) Surface representations of the atomic model for the Brd4^{BD2}-macroPROTAC1-(NEDD8)-CRL2^{VHL} complex (built from the cryo-EM volume from this study aligned with the crystal structure PDB: 6SIS); (**D**) Surface representations of the atomic model of the Brd4^{BD2}-AT7-(NEDD8)-CRL2^{VHL} complex (built from the cryo-EM volume from this study aligned with the crystal structure PDB: 7ZNT); (**E**) Surface representations of the atomic model of the FKBP51^{FK1}-SelDeg51-(NEDD8)-CRL2^{VHL} complex (built from the cryo-EM volume from this study aligned with the crystal structure PDB: 8PC2); (**F**) Surface representations of the atomic model of the Brd4^{BD1}-CIP1-(NEDD8)-CRL2^{VHL} complex (built from the cryo-EM volume from this study aligned with the crystal structure PDB: 8EWV); (**G**) Surface representations of the atomic model of the Brd4^{BD1}-Compound9-(NEDD8)-CRL2^{VHL} complex (built from the cryo-EM volume from this study aligned with the crystal structure PDB: 7KHH); (**H**) Surface representations of the atomic model of the Brd4^{BD1}-PROTAC-48-(NEDD8)-CRL2^{VHL} complex (built from the cryo-EM volume from this study aligned with the crystal structure PDB: 8BDS).

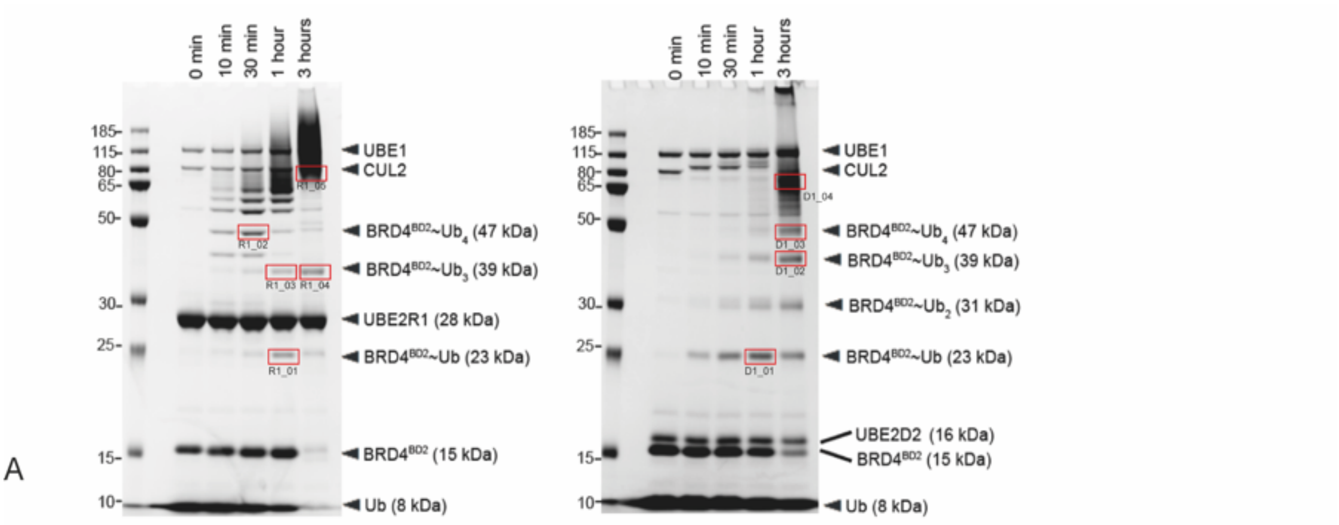

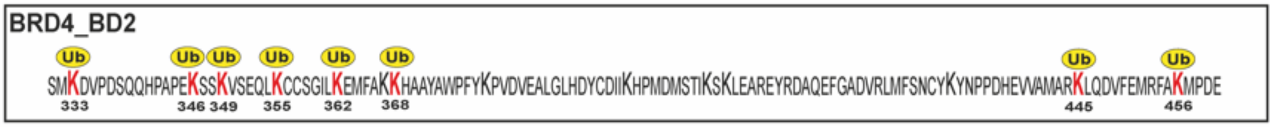

C

	Modified by UBE2R1					Modified by UBE2D2			
Gel slice	R1_01	R1_02	R1_03	R1_04	R1_05	D1_01	D1_02	D1_03	D1_04
BRD4_BD2 peptide sequence coverage (%)	83.1	95.4	50.8	50.0	95.4	95.4	74.6	74.6	95.4

Fig. S6. Identification of Brd4^{BD2} ubiquitination sites by mass spectrometry. (**A**) Coomassie-stained SDS-PAGE of *in vitro* Brd4^{BD2} ubiquitination assays performed in the presence of UBE2R1 or UBE2D2. The red boxes show the regions of the gel that were excised for MS analysis. Below the red box is the name of each slice matching to the raw MS data file and spectra below. (**B**) Linear sequence-based representation of the ubiquitination sites identified on Brd4^{BD2} ('Ub' in yellow circle above lysine residue represents ubiquitin-modification). (**C**) Peptide sequence coverage (%) of Brd4^{BD2} by gel slice.

D

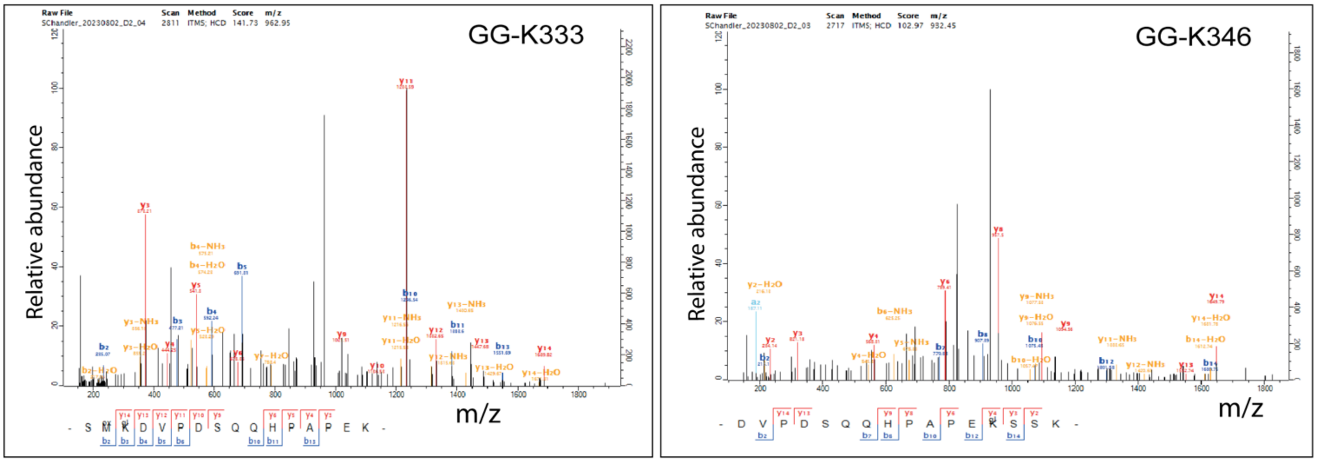

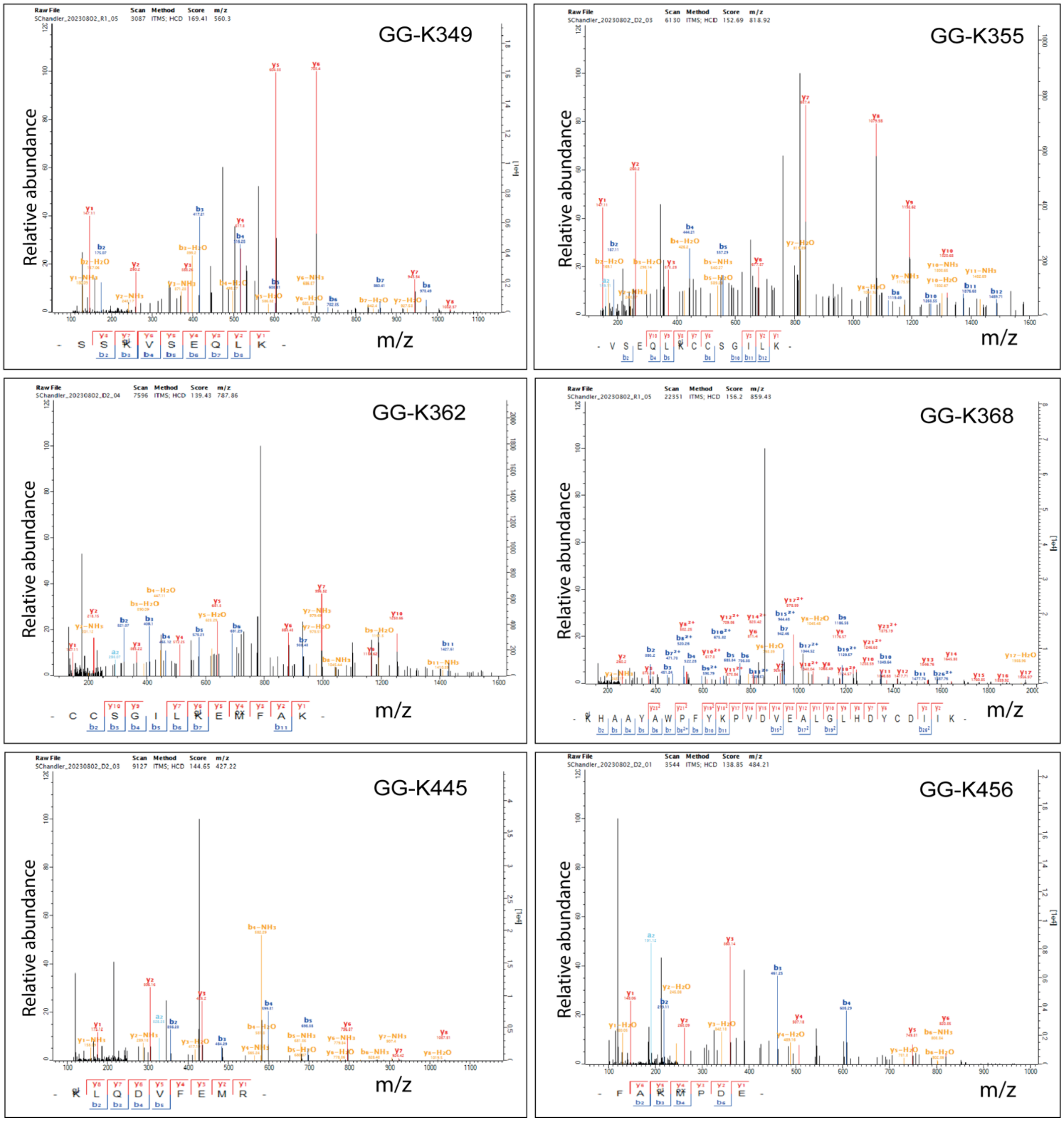

(**D**) Best identified spectra for the Brd4^{BD2} GlyGly(K) modified peptides across all gel slices. Beneath each spectrum is the y (red) and b (blue) series ions that were detected for each peptide. In the top right corner is the GG-K residue, numbering based on the Brd4^{BD2} construct used in the assay, depicted in B. Andromeda score; GG-K333 (141.73), GG-K346 (102.97), GG-K349(169.41), GG-K355(152.69), GG-K362(139.43), GG-K368(156.2), GG-K445(144.65), GG-K456(138.85).

		Modified by UBE2R1										Modified by UBE2D2							
**Time (hrs)**		0.5	1	1	3	3	0.5	1	1	3	3	1	3	3	3	1	3	3	3
**Molecular weight of excised band (kDa)**		47	23	39	39	65	47	23	39	39	79	23	39	47	63	23	39	47	79
**Proteins**	**GG-K Position**	**Andromeda Score**					**Log10 peptide intensity**					**Andromeda Score**				**Log10 peptide intensity**			
BRD4_BD2	333					103					6.26	87	106	115	142	6.05	6.86	7.25	7.47
BRD4_BD2	346					62					6.02	82	88	103	98	6.12	6.69	7.16	7.31
BRD4_BD2	349					169					6.74	128	109	150	126	7.39	7.16	7.45	7.74
BRD4_BD2	355					113					6.77	127	101	153	131	6.78	7.28	7.66	8.14
BRD4_BD2	362											64	91	78	139	6.23	6.51	6.83	7.13
BRD4_BD2	368					156					8.88				137				7.96
BRD4_BD2	445					86					7.18	79	114	145	98	6.33	7.00	7.17	7.28
BRD4_BD2	456	91				107	7.44				8.10	139	128	128	121	8.27	8.58	8.89	9.04
UBIQUITIN	6	122	111		71	159	6.76	6.63		6.48	7.53		176	158	201		7.39	7.75	8.55
UBIQUITIN	11	141	182	127	136	331	7.47	7.55	6.37	7.63	9.13	135	267	273	362	7.19	9.08	9.53	10.08
UBIQUITIN	27	163	175			188	6.67	6.47			7.44		71	135	93		5.79	6.10	6.71
UBIQUITIN	33													63	99				6.25
UBIQUITIN	48	199	172	116	163	320	10.22	10.26	7.97	9.16	10.74	112	159	178	179	7.11	7.40	9.02	9.99
UBIQUITIN	63	108	139		126	285	7.30	7.44		7.23	8.79	59	223	236	239	6.07	8.64	9.56	10.19

Fig. S7. Ubiquitination sites on Brd4^{BD2} were mapped by mass spectrometry following an *in vitro* ubiquitination assay containing neddylated-CRL2^{VHL}, Brd4^{BD2}, MZ1, Uba1 and either UBE2R1 or UBE2D2. Time points were taken at 0 minutes, 10 minutes, 0.5 hours, 1 hour and 3 hours. Proteins were separated by SDS-page analysis. Gel slices containing ubiquitin modified Brd4^{BD2} across timepoints and increasing molecular weight were excised from the gel and were analyzed by mass spectrometry. The Log10 peptide intensities and Andromeda scores of the identified Brd4^{BD2} and ubiquitin peptides are listed.

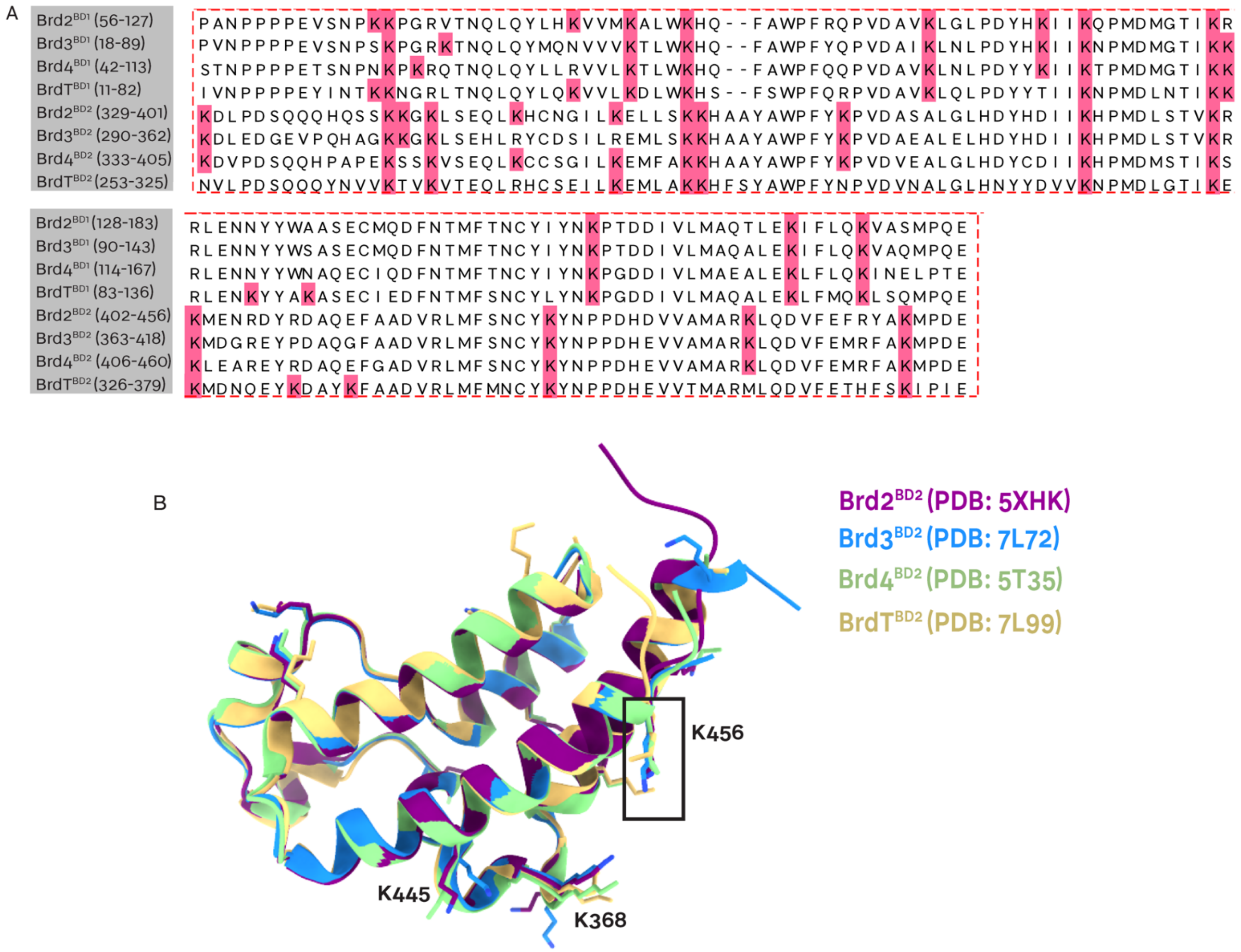

Fig. S8. Sequence and structural alignments of BET bromodomains, highlighting lysine residues available for ubiquitination. (**A**) Multiple sequence alignment of bromodomain-1 and bromodomain-2 for Brd2, Brd3, Brd4, and BrdT. The position of all lysines residues is highlighted in pink. (**B**) Structural alignment of bromodomain-2 of Brd2 (purple), Brd3 (blue), Brd4 (green), and BrdT (yellow) from the crystal structures PDB: 5XHK, 7L72, 5T35 and 7L99. The lysine residues are shown as sticks with colouring according to the bottom legend. K456 of Brd4^{BD2} is annotated, along with K445 and K368.

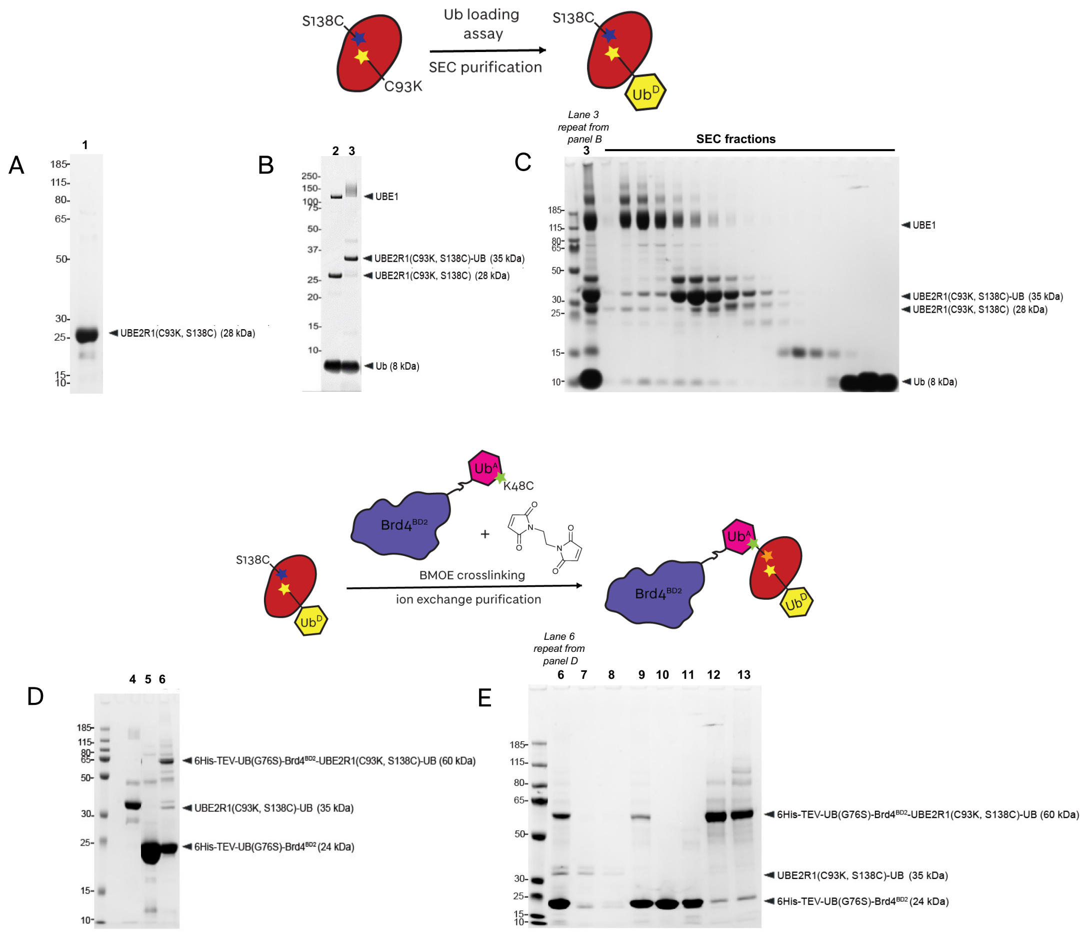

Fig. S9. Preparation of the 6His-TEV-Ub(G76S)-Brd4^{BD2}-BMOE-UBE2R1(C93K,S138C, C191S,C223S)-Ub species for the 'closed' crosslinked cryo-EM structure, resolved by SDS-PAGE and Coomassie staining. (**A**) Lane 1: UBE2R1(C93K,S138C,C191S,C223S) is expressed and purified. (**B**) Lane 2: Ube1 and ubiquitin are added to UBE2R1(C93K,S138C,C191S,C223S), the reaction is at the 0 minute timepoint ; Lane 3: ATP has been added, the reaction is at the 18 hour timepoint and UBE2R1(C93K,S138C,C191S,C223S) is loaded with ubiquitin forming UBE2R1(C93K,S138C,C191S,C223S)-Ub. (**C**) Lane 3: the reaction mixture where UBE2R1(C93K,S138C,C191S,C223S)-Ub is formed is purified by SEC shown in Fractions 16 to 32; (**D**) Lane 4: purified UBE2R1(C93K,S138C,C191S,C223S)-Ub is reacted with BMOE; Lane 5: 6His-TEV-Ub(G76S,K48C)-Brd4^{BD2} is expressed, purified and desalted; Lane 6: BMOE-UBE2R1(C93K,S138C,C191S,C223S)-Ub is reacted with 6His-TEV-Ub(G76S,K48C)-Brd4^{BD2}, forming 6His-TEV-Ub(G76S,K48C)-Brd4^{BD2}-BMOE-UBE2R1(C93K,S138C,C191S,C223S)-Ub; (**E**) Lane 6: the crude reaction mixture where 6His-TEV-Ub(G76S,K48C)-Brd4^{BD2}-BMOE-UBE2R1(C93K,S138C,C191S,C223S)-Ub is formed; Lane 7: Ni NTA flow-through (20 mM imidazole); Lane 8: Ni NTA wash (20 mM imidazole); Lane 9: Ni NTA elution (500 mM imidazole); Lanes 10-11: ion exchange chromatography elution fractions containing excess 6His-TEV-Ub(G76S,K48C)-Brd4^{BD2}; Lanes 12-13: ion exchange chromatography elution fractions containing the purified product 6His-TEV-Ub(G76S,K48C)-Brd4^{BD2}-BMOE-UBE2R1(C93K,S138C,C191S,C223S)-Ub

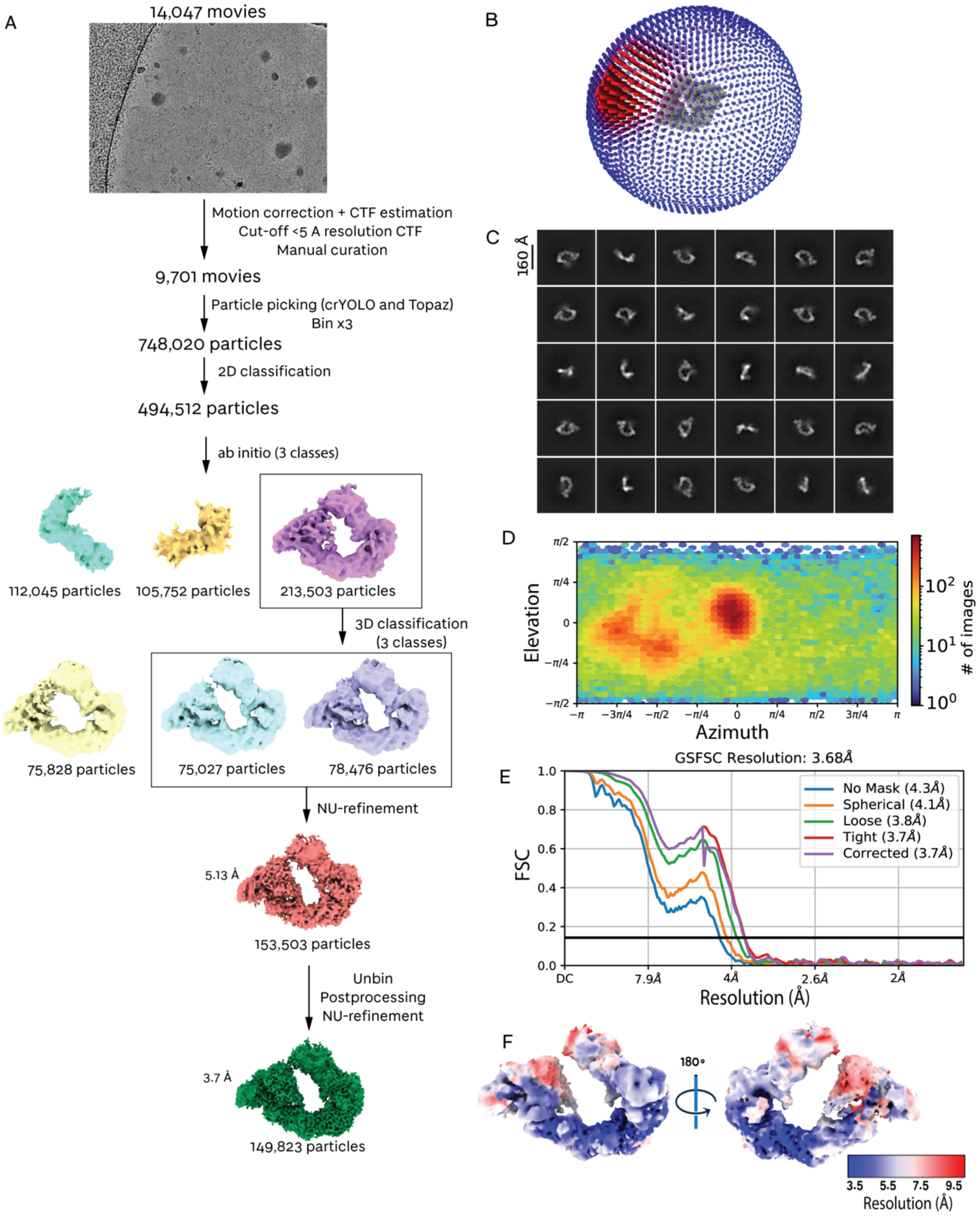

Fig. S10. Cryo-EM image analysis for the 'closed' crosslinked (NEDD8)-CRL2^{VHL}-MZ1-Brd4^{BD2}-Ub(G76S, K48C)-UBE2R1(C93K, S138C, C191S, C223S)-Ub complex. (**A**) A schematic for the processing workflow for the 'closed' crosslinked (NEDD8)-CRL2^{VHL}-MZ1-Brd4^{BD2}-Ub(G76S, K48C)-UBE2R1(C93K, S138C, C191S, C223S)-Ub complex used to generate cryo-EM maps. (**B**) 3D viewing direction distribution. (**C**) Selected 2D classes. (**D**) 2D viewing direction distribution. (**E**) Gold-standard Fourier shell correlation plot at 0.143. (**F**) Local resolution estimation.

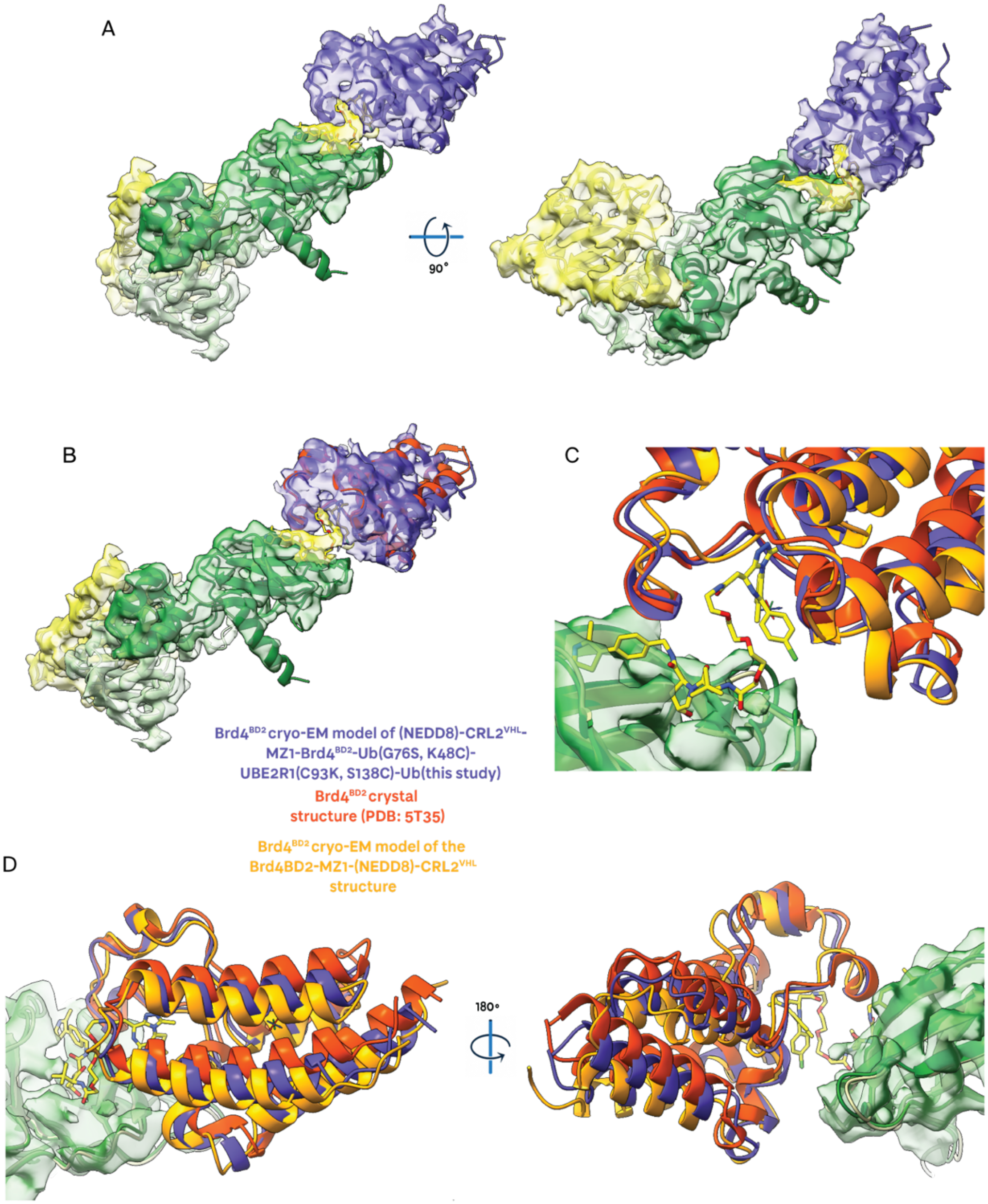

Fig. S11. Cryo-EM volume and fitted atomic models from the 'closed' crosslinked structure of (NEDD8)-CRL2^{VHL}-MZ1-Brd4^{BD2}-Ub(G76S, K48C)-UBE2R1(C93K, S138C, C191S, C223S)-Ub. (**A**) Cryo-EM map (transparent volume) and atomic model for the (NEDD8)-CRL2^{VHL}-MZ1-Brd4^{BD2}-Ub(G76S, K48C)-UBE2R1(C93K, S138C, C191S, C223S)-Ub structure. (**B**) Cryo-EM map (transparent volume) and atomic model for the (NEDD8)-CRL2^{VHL}-MZ1-Brd4^{BD2}-Ub(G76S, K48C)-UBE2R1(C93K, S138C, C191S, C223S)-Ub structure (purple). The atomic model for the crystal structure 5T35 overlays exactly with the atomic model from cryo-EM (alignment against VHL), with deviation observed for Brd4^{BD2} (red). (**C**) The VHL-MZ1-Brd4^{BD2} interface with Brd4^{BD2} from (NEDD8)-CRL2^{VHL}-MZ1-Brd4^{BD2}-Ub(G76S,

K48C)-UBE2R1(C93K, S138C, C191S, C223S)-Ub shown in purple (this study), Brd4^{BD2} from the crystal structure (PDB: 5T35) shown in red, and the Brd4^{BD2} from Brd4^{BD2}-MZ1-(NEDD8)-CRL2^{VHL}-UBE2R1(C93K, S138C, C191S, C223S)-Ub shown in orange (this study). (**D**) Visualisation of the re-orientation of Brd4^{BD2} relative to the interface VHL-MZ1-Brd4^{BD2} interface, with Brd4^{BD2} from (NEDD8)-CRL2^{VHL}-MZ1-Brd4^{BD2}-Ub(G76S, K48C)-UBE2R1(C93K, S138C, C191S, C223S)-Ub shown in purple (this study), Brd4^{BD2} from the crystal structure (5T35) shown in red, and the Brd4^{BD2} from Brd4^{BD2}-MZ1-(NEDD8)-CRL2^{VHL}-UBE2R1(C93K, S138C, C191S, C223S)-Ub shown in orange (this study).

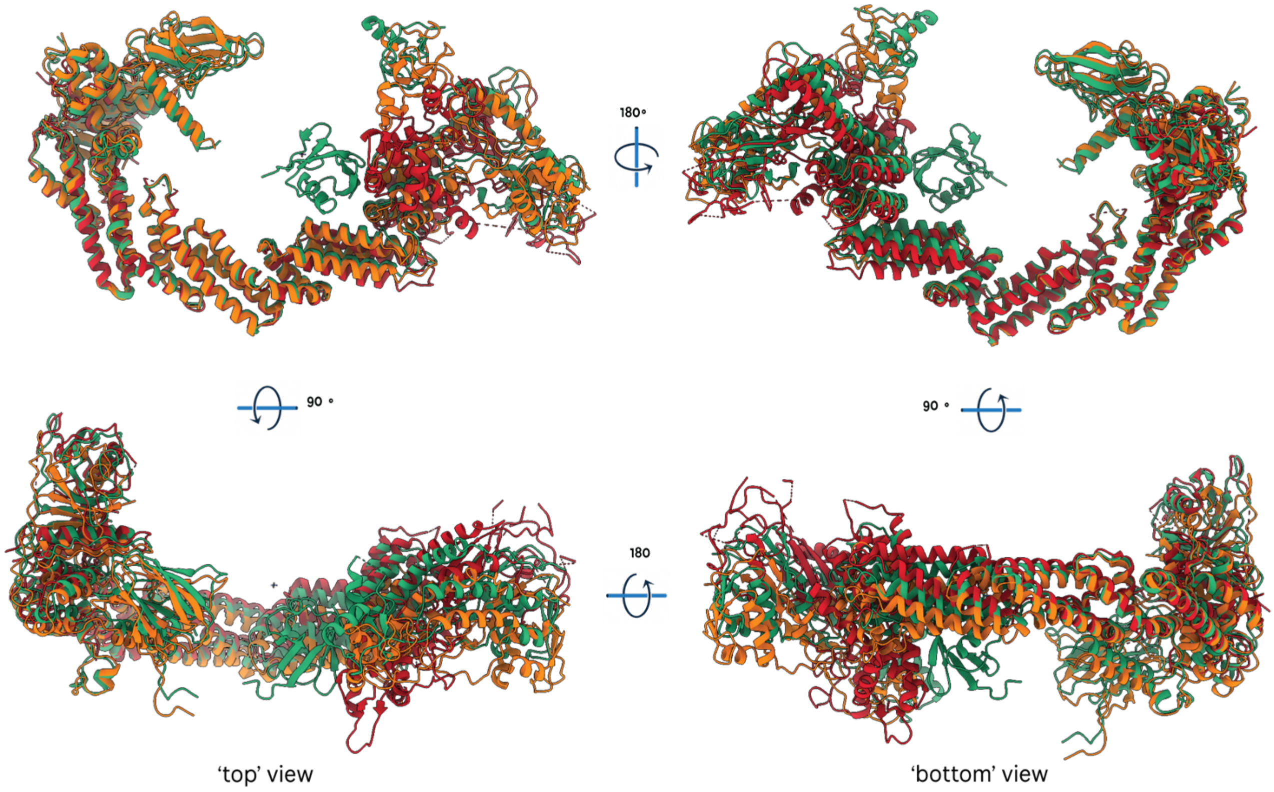

Fig. S12. Superimposition of CRL2^{VHL} atomic models. Atomic models include: the atomic model built into the cryo-EM map of the (NEDD8)-CRL2^{VHL}-MZ1-Brd4^{BD2}-Ub(G76S, K48C)-UBE2R1(C93K, S138C, C191S, C223S)-Ub structure (this study, green); the atomic model built into the cryo-EM map of the Brd4^{BD2}-MZ1-(NEDD8)-CRL2^{VHL}-UBE2R1(C93K)-Ub structure (this study, orange); the atomic model of the crystal structure (PDB: 5N4W) of the unneddylated CRL2^{VHL} (red). The atomic models were aligned again the first 170 residues of Cullin 2. Hinging of Cullin 2 was observed at the C-terminus of the helical bundle 2, leading to a range of conformations being sampled for the Cullin C-terminal domain. Rbx1 adopted different conformations dependent on whether the complex was neddylated, crosslinked with UBE2R1 or non-crosslinked.

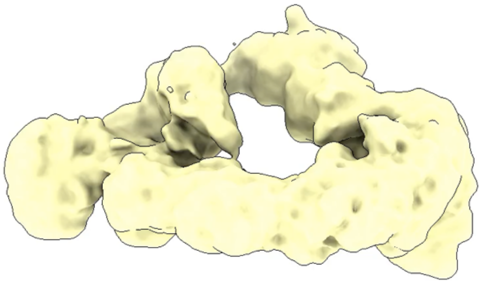 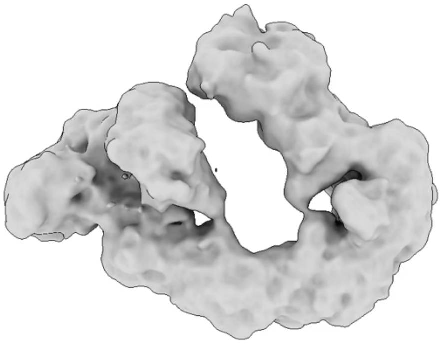

Movies S1 and S2. Results of 3D Variability analysis of the 'closed' crosslinked structure (NEDD8)-CRL2^{VHL}-MZ1-Brd4^{BD2}-Ub(G76S, K48C)-UBE2R1(C93K, S138C, C191S, C223S)-Ub. The analysis was performed on 149,823 particles in CryoSPARC v4.4.1 (*61, 78*). The output was clustered into groups of 20 frames and combined as linear 'movies' of volumes. The trajectories displayed highlight the extent to which the complex is dynamic and outline the range of motion available.

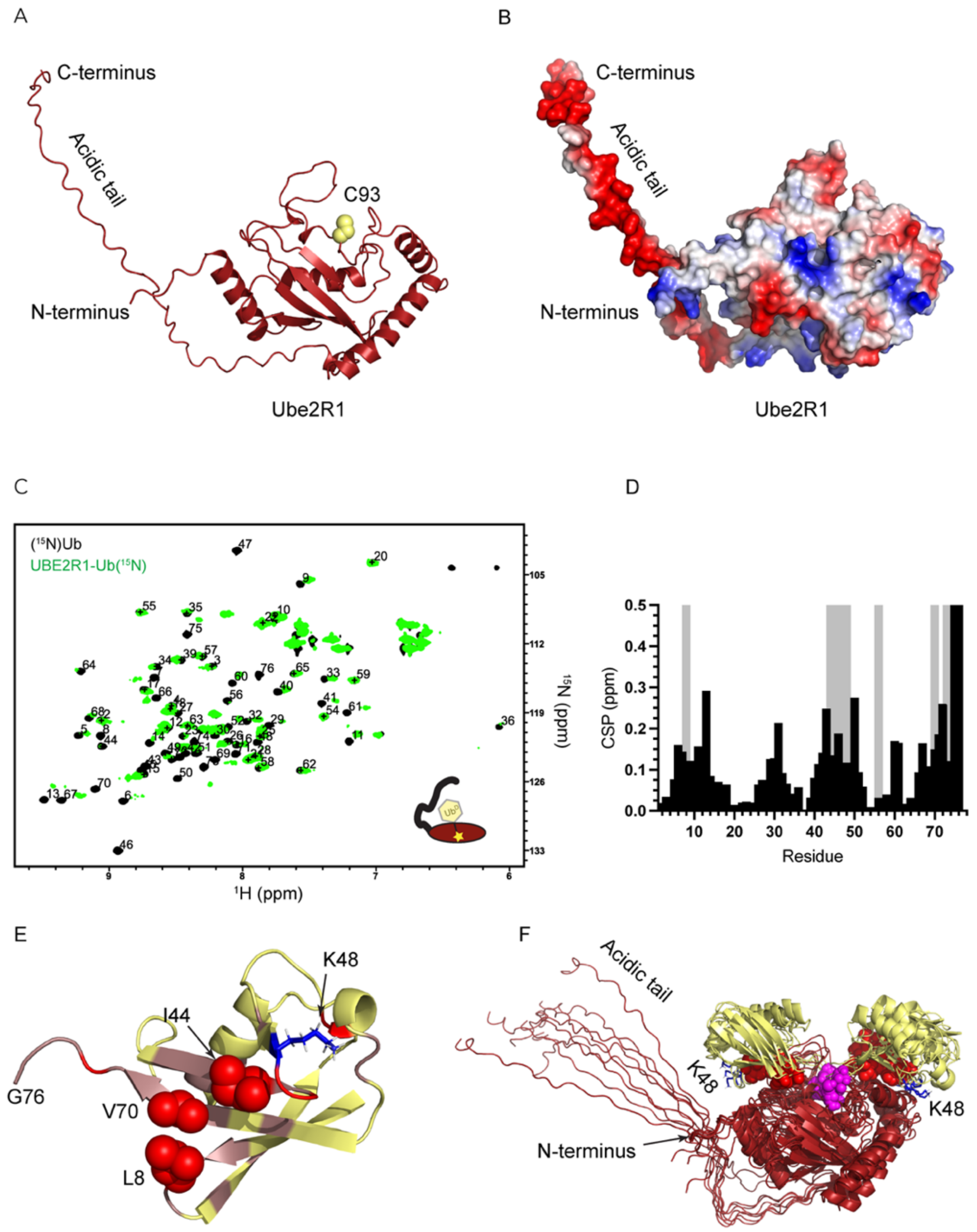

Fig. S13. NMR analysis of Ub^{D} on UBE2R1. The stable UBE2R1(C93K)-Ub(^{15}N) conjugate was made on full length human UBE2R1 with (**A**) AlphaFold2 model and (**B**) surface representation with electrostatic potential highlighting the acidic tail and regulator loops on the active site C93. (**C**) Overlay of ^{1}H-^{15}N-HSQC spectra of unconjugated Ub(^{15}N) (black) and UBE2R1(C93K)-Ub(^{15}N) (green). (**D**) Residue specific CSPs (black bars) and signal attenuations (grey) for residues. (**E**) Signal attenuations (red) and above average CSPs (salmon) are mapped on Ub highlighting the interaction with the L8, I44, V70 hydrophobic patch and K48 (red stick). (**F**) Poses from top clusters of modeling Ub^{D} on the active site of UBE2R1 with HADDOCK (*90*), restrained with C93 of UBE2R1 and G76 of Ub^{D} (magenta spheres). Residues from Ub^{D} model the interaction through hydrophobic patch (L8, I44, V70) green sticks and K48 as blue sticks.

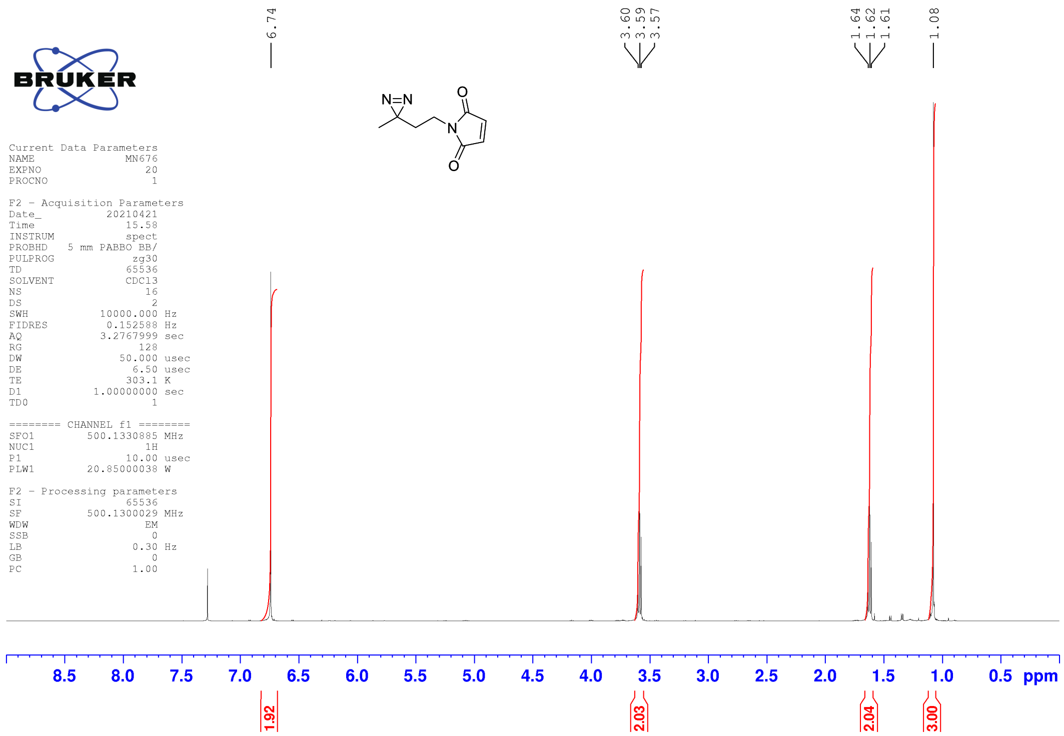

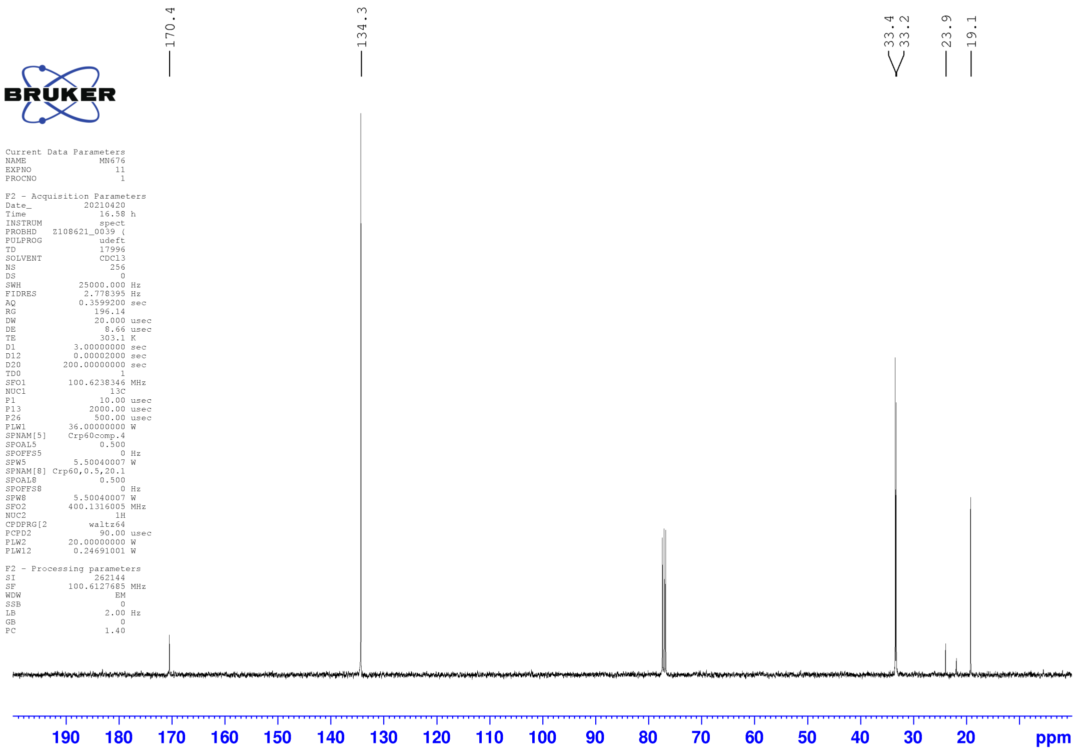

Fig. S14. NMR spectra of 1-(2-(3-methyl-3H-diazirin-3-yl)ethyl)-1H-pyrrole-2,5-dione.

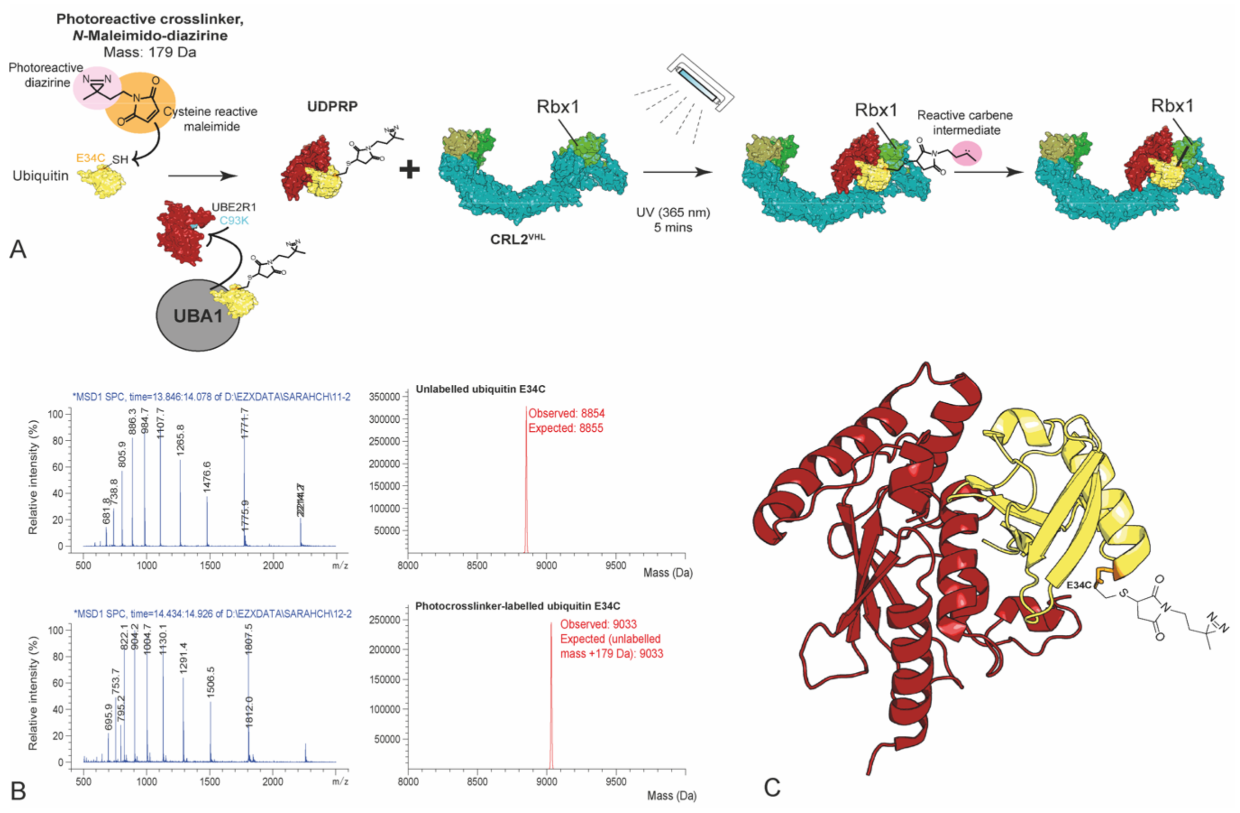

Fig. S15. Design of ubiquitin-directed photoreactive probe (UDPRP) to capture CRL2^{VHL} in-solution. The production of the probe starts with the site-specific labelling of Ub(E34C) with the photoreactive crosslinker *N*-maleimido-diazirine. The UDPRP is generated by conjugating the photocrosslinker-labelled ubiquitin to UBE2R1(C93K) using recombinant human E1 (UBA1) forming a stable isopeptide linked E2-Ub conjugate. The UDPRP is then purified by size exclusion chromatography. The modified UBE2R1(C93K)-Ub(E34C)-crosslinker UDPRP species now displays the photoreactive crosslinker *N*-maleimido diazirine which when exposed to UV light (365 nm) forms a reactive carbene intermediate which can rapidly insert into X-H bonds (including O-H, N-H, S-H and C-H bonds) and can therefore trap proteins in proximity (*77*). This forms a crosslinked product (MW 47 kDa) that is resistant to separation by SDS-PAGE analysis and can therefore be visualized by Coomassie staining or by immunoblotting for components of the photo-crosslinked product, or the photo-crosslinked peptide can be detected by mass-spectrometry. (**A**) Schematic representation of the design and application of the UDPRP. Upon reaction with (NEDD8)-CRL2^{VHL} and exposure to UV light (365 nm), the carbene can react with X-H bonds in Rbx1. The models were generated using the PDB codes: ubiquitin (1UBQ), UBA1 in complex with ubiquitin (6DC6), structure of UBE2R2 (3RZ3) was used for UBE2R1, CRL2^{VHL} (5N4W). (**B**) Site specific labelling of Ub(E34C) with the photocrosslinker *N*-maleimido-diazirine. Raw ESI-MS spectrum of samples containing unlabeled and labelled Ub(E34C). Observed mass shift was equal to the mass of *N*-maleimido-diazirine (179 Da). (**C**) Structural representation of the UDPRP, the model was generated using the crystal structure of a stable isopeptide-linked UBE2R2-Ub conjugate (PDB: 6NYO). The presented crystal structure contains wild-type ubiquitin however in the UDPRP an E48C mutant ubiquitin was used to enable the site-specific labelling of *N*-maleimido-diazirine.

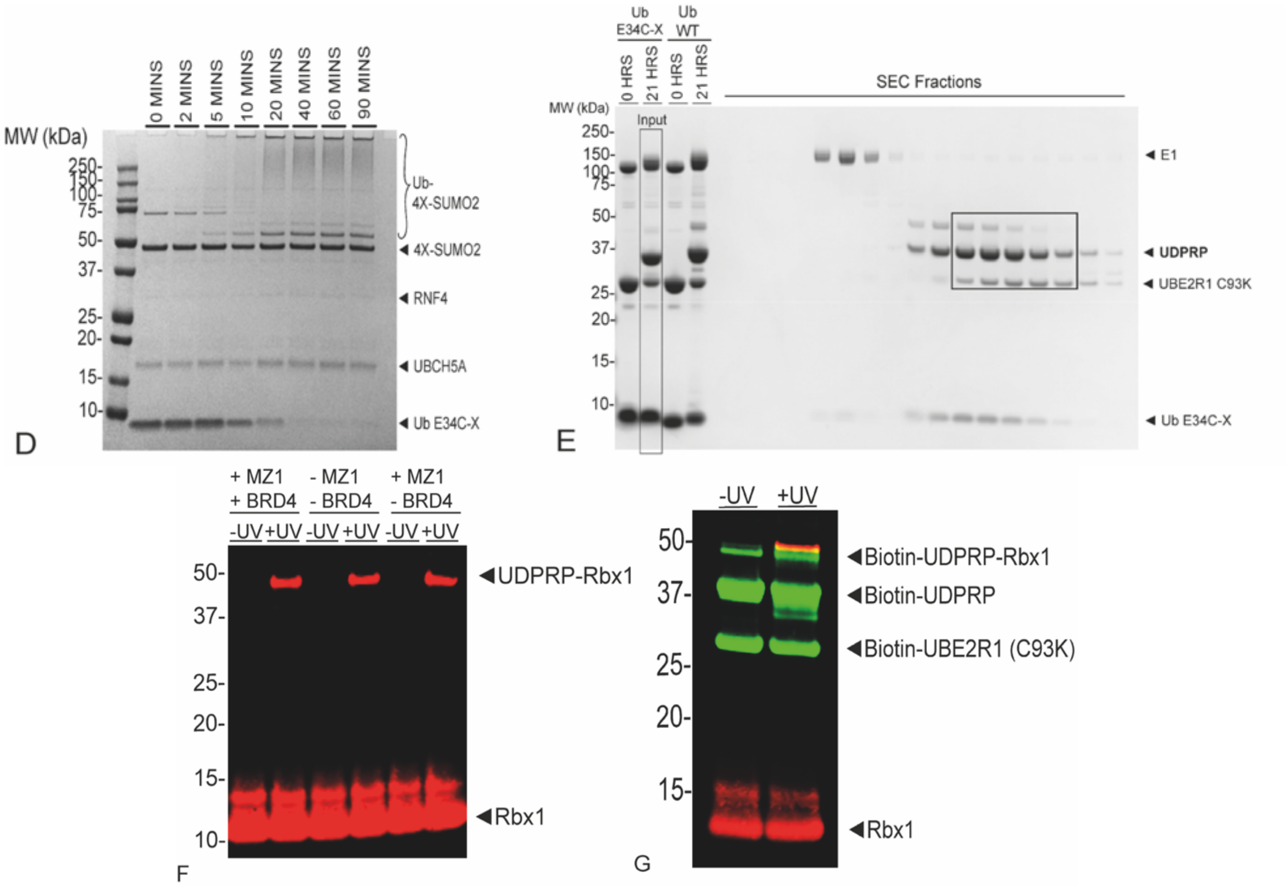

(**D**) Coomassie stained gel to demonstrate that the photocrosslinker-labelled ubiquitin E34C (Ub E34C-X) is active in an *in vitro* ubiquitination assay with the SUMO-targeted E3 ligase, the E2 UBCH5A and UBA1. (**E**) Coomassie stained gel of the preparative loading of photocrosslinker-labelled Ub(E34C) to UBE2R1(C93K) and purification of the ubiquitin-directed photoreactive probe (UDPRP). The loading efficiency of photocrosslinker-labelled ubiquitin (Ub E34C-X) to the active site of UBE2R1 is comparable to wild-type ubiquitin. A 0-hour timepoint taken prior to the addition of ATP, after 21 hours the reaction containing the Ub E34C-X (input) was separated by size-exclusion chromatography. The black box indicates the fractions which were pooled. (**F**) Immunoblot against Rbx1 shows the recruitment of the UDPRP to Rbx1 occurs independently of BRD4^{BD2} and MZ1. UDPRP photo-crosslinking assay containing (NEDD8)-CRL2^{VHL} (3 μM), UDPRP (10 μM), +/- BRD4^{BD2} (17 μM), +/- MZ1 (4.5 μM). (**G**) Immunoblot for Rbx1 (red) and biotinylated-UBE2R1 (green) indicates that the photo-crosslinked product (orange) contains biotinylated UDPRP (UBE2R1-Ub) and Rbx1. UDPRP photo-crosslinking assay containing (NEDD8)-CRL2^{VHL} (3 μM) and biotinylated-UDPRP (10 μM). The biotinylated-UDPRP contained some unmodified UBE2R1(C93K), a remnant of the ubiquitin loading assay.

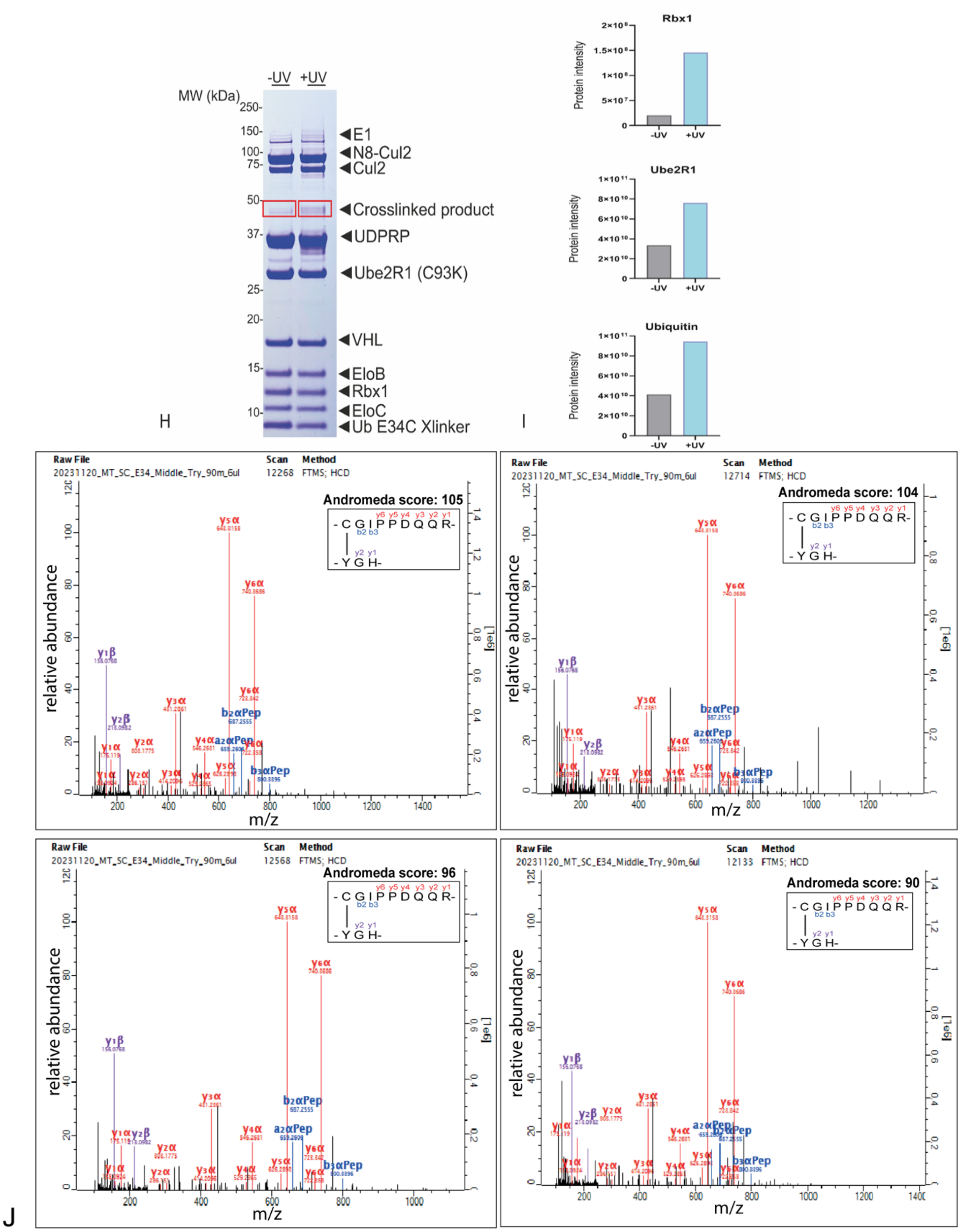

(**H**) Coomassie stained gel of the photocrosslinking UDPRP assay for MS analysis. The red boxes show the regions of the gel that were excised. (**I**) Protein intensity determined by mass spectrometry verifies the presence of Rbx1 in the photo-crosslinked product. Intensities for Rbx1, UBE2R1, and Ub from the two bands shown in red in (**H**)**,** left hand side (-UV) and right-hand side (+UV). (**J**) Ubiquitin-Rbx1 inter-protein crosslinked peptides detected linking Ub(E34C) to the C-terminus of Rbx1. Spectra for crosslinked peptides identified in the +UV gel

slice (shown in (**H**)). The crosslinked peptide is shown in the top right-hand corner with the y (purple and red) and b (blue) series ions detected.

A

	UBE2R1 atom	Brd4BD2 atom	Distance (Å)
Cα-atom to Cα-atom	**C93 Cα-atom**	**K456 Cα-atom**	**24.08**
	C93 Cα-atom	K445 Cα-atom	19.58
	C93 Cα-atom	K368 Cα-atom	17.62
	C93 Cα-atom	K362 Cα-atom	26.58
	C93 Cα-atom	K355 Cα-atom	32.64
Thioester carbonyl C-atom to N-atom	**C93 thioester carbonyl C-atom**	**K456 N-atom**	**18.41**
	C93 thioester carbonyl C-atom	K445 N-atom	19.22
	C93 thioester carbonyl C-atom	K368 N-atom	15.15
	C93 thioester carbonyl C-atom	K362 N-atom	27.92
	C93 thioester carbonyl C-atom	K355 N-atom	35.81

B

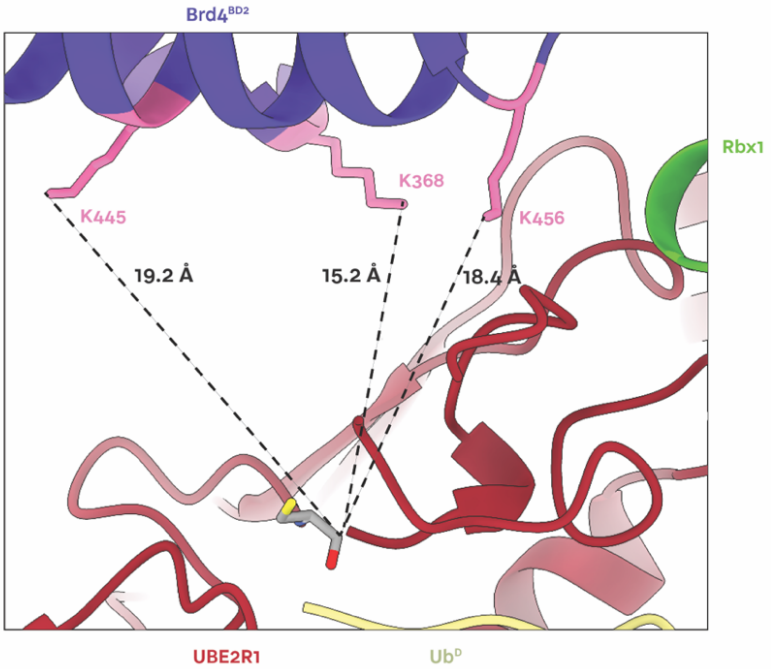

Fig. S16. Measurements between ubiquitinated lysine residues on Brd4^{BD2} (as identified by mass spectrometry) and the catalytic cysteine site of UBE2R1. (**A**) Summary of the lysine residues on Brd4^{BD2} identified by mass spectrometry as being ubiquitinated by UBE2R1 *in vitro*. The measurements were performed between the Cα carbons of the ubiquitinated Lys and the Cα carbon of the Cys93 residue of UBE2R1. Measurements were also performed between the nucleophilic nitrogen atom of the ubiquitinated Lys side-chain, and the Cys93 thioester carbonyl C-atom of UBE2R1, corresponding to electrophilic carboxyl targeted during ubiquitination. Measurements were performed using UCSF ChimeraX (*82*) based on the cryo-EM model in this study. (**B**) Schematic representation of the distances measured between the three closest lysine residues of Brd4^{BD2} to the active site cysteine site of UBE2R1. All three lysines appear to be positioned at a similar distance but adopt different geometries for the nucleophilic attack of the active site cysteine. Modelling was performed using UCSF ChimeraX (*82*) based on the cryo-EM model in this study.

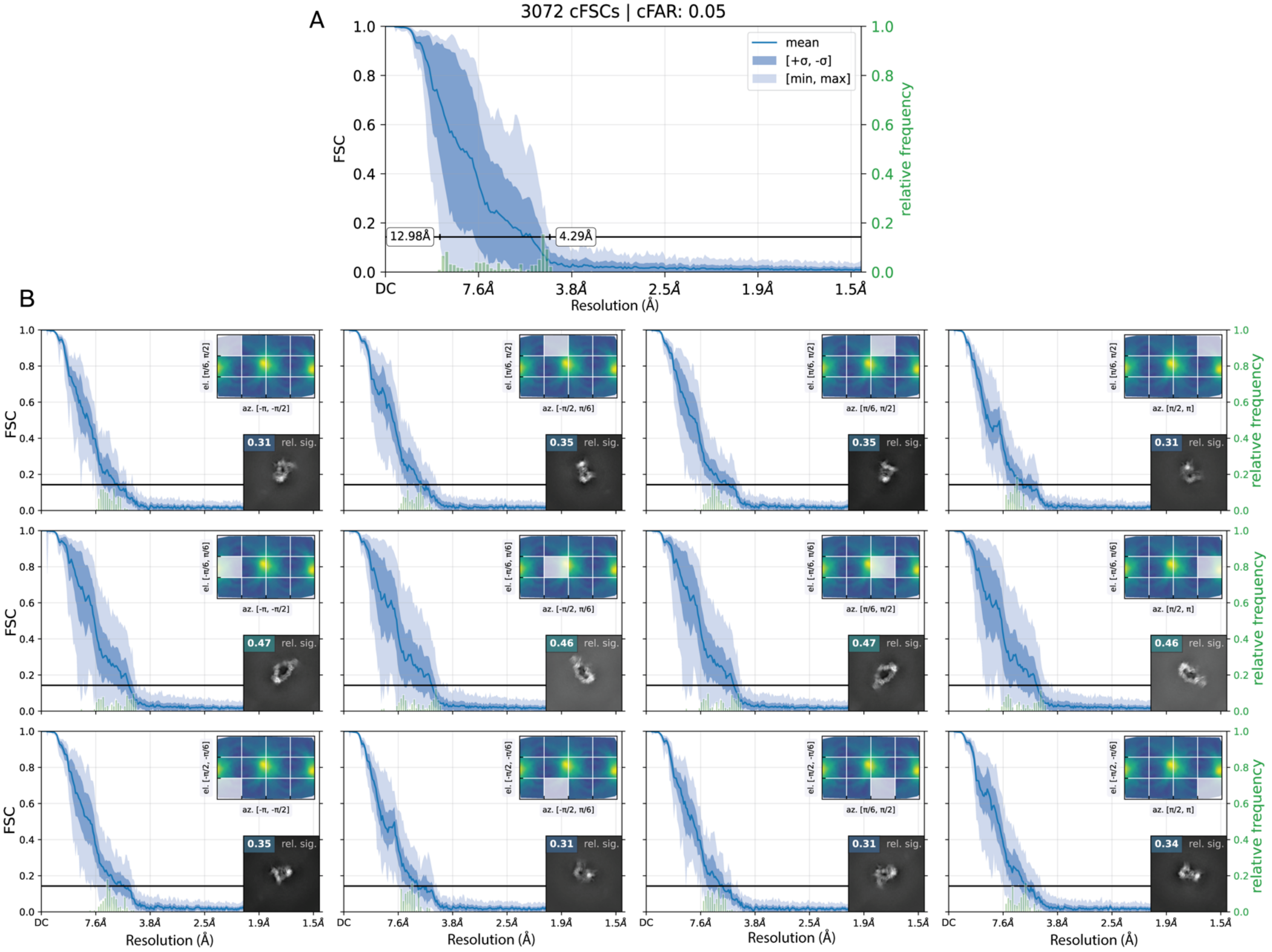

Fig. S17. Cryo-EM image analysis for Brd4^{BD2}-MZ1-(NEDD8)-CRL2^{VHL}-UBE2R1-Ub. (**A**) Conical FSC curve of the cryo-EM volume. **(B)** Relative signal of twelve regions of the viewing sphere, with cryo-EM volume 2D projection and conical FSC.

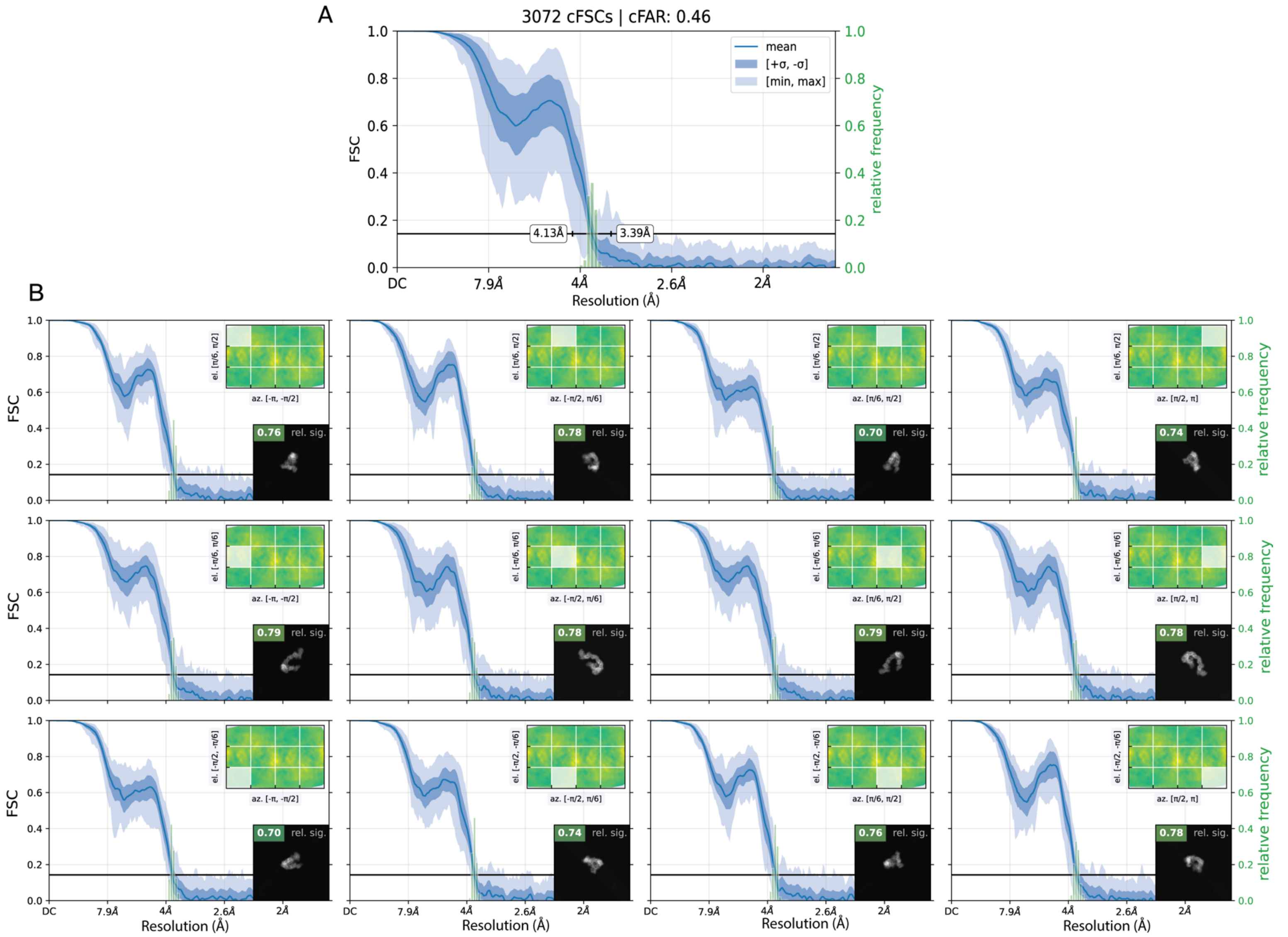

Fig. S18. Directional cryo-EM volume analysis for (NEDD8)-CRL2^{VHL}-MZ1-Brd4^{BD2}-Ub(G76S, K48C)-UBE2R1(C93K, S138C, C191S, C223S)-Ub complex. (**A**) Conical FSC curve of the cryo-EM volume. **(B)** Relative signal of twelve regions of the viewing sphere, with cryo-EM volume 2D projection and conical FSC.

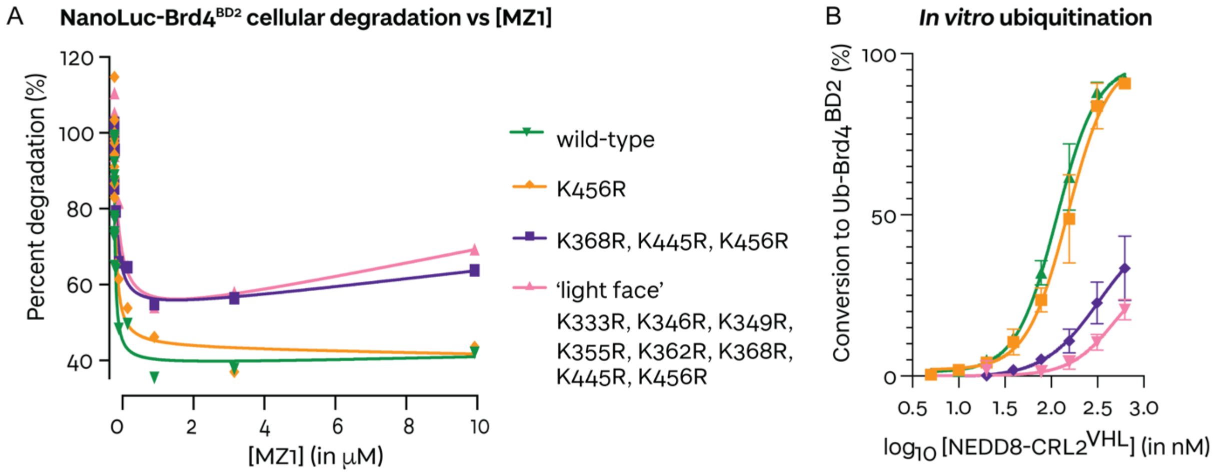

Fig. S19. *In vitro* ubiquitination and cellular degradation with lysine mutant Brd4 bromodomain 2 constructs. (**A**) Cellular degradation of NanoLuc-Brd4^{BD2} wild-type and mutant constructs in HEK293 cells after 4-hour treatment with MZ1, as a function MZ1 concentration. (**B**) *In vitro* ubiquitination of Brd4^{BD2} wild-type and mutant constructs as a function of NEDD8-CRL2^{VHL}.

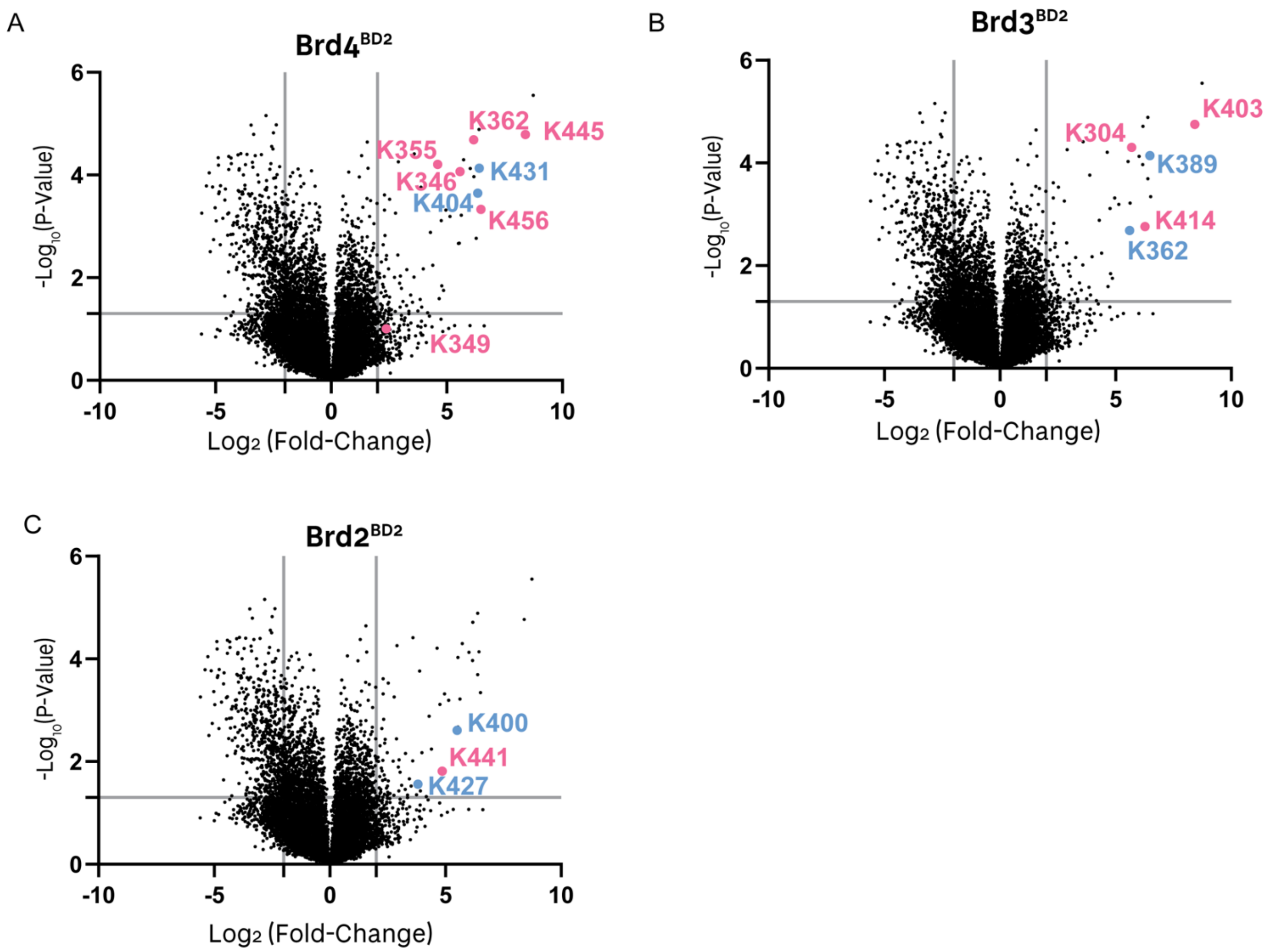

Fig. S20. Global ubiquitylome changes, as assessed by di-glycine remnant mass spectrometry profiling, for (**A**) Brd4^{BD2}, (**B**) Brd3^{BD2} and (**C**) Brd2^{BD2}. The depicted volcano plots show ubiquitinated precursors presented as log2 fold-change observed in HEK293 cells following treatment with 1 μM MZ1 for 15 minutes relative to DMSO controls. Positions of ubiquitinated lysines in peptides that uniquely map to Brd4^{BD2} designated 'light face' are highlighted in pink, while 'dark face' lysines are highlighted in blue, respectively. Assignment of lysine residues to 'light face' vs 'dark face' for the BD2s of Brd2 and Brd3 are based on a structural superposition of those domains onto the Brd4^{BD2} structure.

A

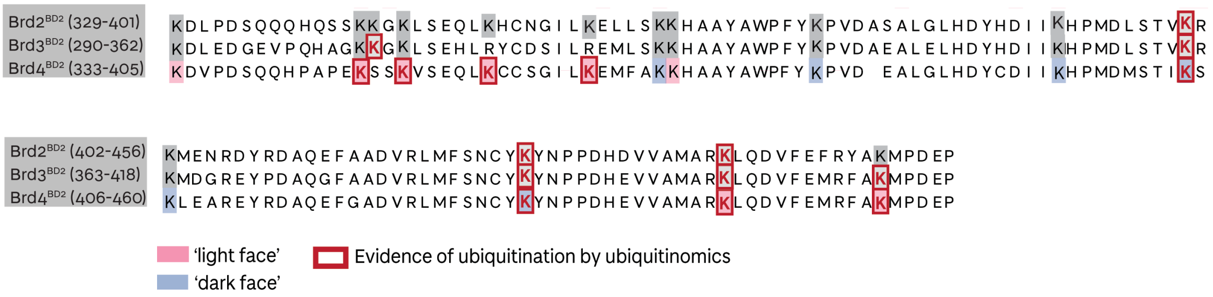

B

Bromodomain	Total number of ubiquitinated lysines by ubiquitinomics	Number and fraction of ubiquitinated lysines mapping to Brd4^{BD2} 'light face'	Number and fraction ubiquitinated lysines mapping to Brd4BD2 'dark face'
Brd4^{BD2}	8	**6/8 (75%)**	**2/6 (33%)**
Brd3^{BD2}	5	**5/13 (38% of all Lys across the whole domain)**	
Brd2^{BD2}	3	**3/15 (20% of all Lys across the whole domain)**	

Fig. S21. Summary of ubiquitinated lysine residues. (**A**) Sequence alignments of the second bromodomains (BD2s) of Brd2, Brd3 and Brd4. (**B**) Total number and fraction of lysine residues on Brd2^{BD2}, Brd3^{BD2} and Brd4^{BD2}, identified as ubiquitinated in the cellular ubiquitinomics data, and analysis of those which map to the assigned 'light face' and 'dark face' of Brd4^{BD2} based on *in vitro* ubiquitination data.

A

Site	-Log10(P-Value)	Log2(Fold-change)	Gene	Modified sequence
K400	2.665578439	5.510783513	BRD2	HPMDLSTVK(UniMod:121)R
K118	0.964241452	1.899916013	BRD2	IIK(UniMod:121)QPMDMGTIK
K441	1.843267101	4.781194687	BRD2	K(UniMod:121)LQDVFEFR
K115	0.704112067	2.799409866	BRD2	LGLPDYHK(UniMod:121)IIK
K427	1.348501317	3.359655062	BRD2	LMFSNC(UniMod:4)YK(UniMod:121)YNPPDHDVVAMAR
K127	1.216832959	3.194902738	BRD2	QPMDMGTIK(UniMod:121)R
K107	4.129718971	6.014557838	BRD2	QPVDAVK(UniMod:121)LGLPDYHK

B

Site	-Log10(P-Value)	Log2(Fold-change)	Gene	Modified sequence
K304	4.298872776	5.727699598	BRD3	DLEDGEVPQHAGK(UniMod:121)K
K414	2.768244363	6.271925608	BRD3	FAK(UniMod:121)MPDEPVEAPALPAPAAPMVSK
K75	1.428377996	3.91220665	BRD3	LNLPDYHK(UniMod:121)IIK
K362	2.665578439	5.510783513	BRD3	HPMDLSTVK(UniMod:121)R
K403	4.766326242	8.410702387	BRD3	K(UniMod:121)LQDVFEMR
K389	4.139471123	6.445173899	BRD3	LMFSNC(UniMod:4)YK(UniMod:121)YNPPDHEVVAMAR

C

Site	-Log10(P-Value)	Log2(Fold-change)	Gene	Modified sequence
K362	4.71483924	6.18745931	BRD4	C(UniMod:4)C(UniMod:4)SGILK(UniMod:121)EMFAK
K346	4.207512706	4.635379155	BRD4	DVPDSQQHPAPEK(UniMod:121)SSK
K456	3.216229215	5.631207466	BRD4	FAK(UniMod:121)MPDEPEEPVVAVSSPAVPPPTK
K404	3.340370074	6.516310692	BRD4	HPMDMSTIK(UniMod:121)SK
K445	4.766326242	8.410702387	BRD4	K(UniMod:121)LQDVFEMR
K431	4.139471123	6.445173899	BRD4	LMFSNC(UniMod:4)YK(UniMod:121)YNPPDHEVVAMAR
K99	1.754078661	4.865210851	BRD4	LNLPDYYK(UniMod:121)IIK
K349	0.764106503	2.251405716	BRD4	SSK(UniMod:121)VSEQLK
K111	3.969752296	6.177751859	BRD4	TPMDMGTIK(UniMod:121)K
K355	4.41354375	3.592231115	BRD4	VSEQLK(UniMod:121)C(UniMod:4)C(UniMod:4)SGILK
K72	0.637233604	1.2785031	BRD4	VVLK(UniMod:121)TLWK

Fig. S22. Peptide sequences of Brd2, Brd3 and Brd4 bromodomains which showed evidence of modification in the cellular ‘ubiquitinomics’ mass spectrometry experiment. (**A**) Peptides from Brd2. (**B**) Peptides from Brd3. (**C**) Peptides from Brd4.

	'Open' non-crosslinked structure: Brd4^{BD2}-MZ1-(NEDD8)-CRL2^{VHL}-UBE2R1(C93K)-Ub (EMDB-19569) (PDB 8RWZ)	*'Closed' crosslinked structure: (NEDD8)-CRL2^{VHL}-MZ1-Brd4^{BD2}-Ub(G76S, K48C)-UBE2R1(C93K, S138C, C191S, C223S)-Ub (EMDB-19567) (PDB 8RX0)*
Data collection		
Microscope	Glacios	Krios
Detector	Falcon 4i (counting)	Gatan K3 (counting)
Voltage (kV)	200	300
Magnification (nominal)	190,000x	105,000x
Number of frames collected	640	134
Total electron exposure ($e^-/Å^2$)	26	38
Exposure rate (e^-/pix/s)	7.1	15.1
Defocus range (µm)	-1.7 to -3.2 µm	-1.2 to -3.0 µm
Pixel size at detector (Å/pixel)	0.74	0.825
Total exposure (s)	2.0	1.8
Automation software	EPU	EPU
Energy filter slit width (eV)	N/A	20
Movies collected (#)	4,961	14,047
Movies used (#)	4,338	9,701
Reconstruction		
Image processing package	CryoSPARC	CryoSPARC
Symmetry imposed	C1	C1
Initial particle images (#)	405,567	748,020
Final particle images (#)	132,697	149,823
Map resolution (Å) at FSC 0.143	4.0	3.7
B Factor ($Å^2$)	170.7	103.7
Map resolution range (Å)	4.29 to 12.98	3.39 to 4.13
*SCF**	0.881	0.962
cFAR	0.05	0.49
3DFSC sphericity (%)	71.8	87.8
Model composition		
Proteins	Brd4^{BD2}, NEDD8, Cul2, Rbx1, EloB, EloC, VHL	Brd4^{BD2}, NEDD8, Cul2, Rbx1, EloB, EloC, VHL, UBE2R1(C93K, S138C, S191C, S223C), ubiquitin, ubiquitin (K48C, G76S)
Ligands	MZ1	MZ1
Model refinement		
Atomic modelling packages	iSOLDE, Phenix	iSOLDE, Phenix
Initial model(s) used	5T35, 5N4W, AlphaFold	5T35, 5N4W, 4AP4, 6TTU, AlphaFold
Cross-correlation (map/mask)	0.12/0.12	0.53/0.55
R.m.s deviations from ideal values:		
- Bond lengths (Å)	0.002	0.005
- Bond angles (°)	0.863	1.115
Protein residues B Factor(Å)	10.26	12.64
Ligand B Factor (Å)	11.12	36.14
Validation		
MolProbity score	1.91	1.74
Clashscore (all atoms)	6.85	10.94
Poor rotamers (%)	0.00	0.00
Ramachandran - favoured (%)	90.53	96.90
Ramachandran outliers (%)	0.09	0.00
EMRinger score	N.D.	1.12
CaBLAM outliers (%)	3.84	1.33

Table S1. Cryo-EM data collection, image analysis, atomic modeling, refinement, and validation statistics.
